# SARS-CoV-2 variant with the spike protein mutation F306L in the southern border provinces of Thailand

**DOI:** 10.1038/s41598-024-56646-6

**Published:** 2024-04-02

**Authors:** Thanit Sila, Smonrapat Surasombatpattana, Songyos Rajborirug, Wison Laochareonsuk, Pongsakorn Choochuen, Chanon Kongkamol, Thammasin Ingviya, Napat Prompat, Surakameth Mahasirimongkol, Surasak Sangkhathat, Pakorn Aiewsakun

**Affiliations:** 1 Department of Pathology, Faculty of Medicine, Prince of Songkla University, Hat Yai, Songkhla, 90110 Thailand; 2 Department of Epidemiology, Faculty of Medicine, Prince of Songkla University, Hat Yai, Songkhla, 90110 Thailand; 3 Division of Surgery, Faculty of Medicine, Prince of Songkla University, Hat Yai, Songkhla, 90110 Thailand; 4 Translational Medicine Research Center, Faculty of Medicine, Prince of Songkla University, Hat Yai, Songkhla, 90110 Thailand; 5 Department of Biomedical Sciences and Biomedical Engineering, Faculty of Medicine, Prince of Songkla University, Hat Yai, Songkhla, 90110 Thailand; 6 Department of Family Medicine and Preventive Medicine, Prince of Songkla University, Hat Yai, Songkhla, 90110 Thailand; 7 Faculty of Medical Technology, Medical of Technology Service Center, Prince of Songkla University, Songkhla, 90110 Thailand; 8 Department of Medical Sciences, Genetics Center, Medical Life Sciences Institute, Ministry of Public Health, Nonthaburi, 11000 Thailand; 9 Pornchai Matangkasombut Center for Microbial Genomics, Department of Microbiology, Faculty of Science, Mahidol University, Bangkok, 10400 Thailand; 10 Department of Microbiology, Faculty of Science, Mahidol University, Bangkok, 10400 Thailand

**Keywords:** SARS-CoV-2, Spike protein, Delta variant, AY.85, F306L, Pathogens, Virology, SARS-CoV-2

## Abstract

The southernmost part of Thailand is a unique and culturally diverse region that has been greatly affected by the severe acute respiratory syndrome coronavirus-2 (SARS-CoV-2) outbreak during the coronavirus disease-2019 pandemic. To gain insights into this situation, we analyzed 1942 whole-genome sequences of SARS-CoV-2 obtained from the five southernmost provinces of Thailand between April 2021 and March 2022, together with those publicly available in the Global Initiative on Sharing All Influenza Data database. Our analysis revealed evidence for transboundary transmissions of the virus in and out of the five southernmost provinces during the study period, from both domestic and international sources. The most prevalent viral variant in our sequence dataset was the Delta B.1.617.2.85 variant, also known as the Delta AY.85 variant, with many samples carrying a non-synonymous mutation F306L in their spike protein. Protein–protein docking and binding interface analyses suggested that the mutation may enhance the binding between the spike protein and host cell receptor protein angiotensin-converting enzyme 2, and we found that the mutation was significantly associated with an increased fatality rate. This mutation has also been observed in other SARS-CoV-2 variants, suggesting that it is of particular interest and should be monitored.

## Introduction

The continual emergence of new variants of severe acute respiratory syndrome coronavirus-2 (SARS-CoV-2), a novel human viral pathogen that causes the coronavirus disease-2019 (COVID-19), has brought unprecedented challenges to global public health, and Thailand is no exception. While Thailand successfully controlled the spread of the virus in the first year of the pandemic in 2020^1,2^, the country saw nationwide outbreaks of the virus in 2021, predominantly caused by the B.1.617.2 and AY (Delta) variants^2,3^; and in 2022, predominantly caused by the B.1.1.529 and BA (Omicron) variants^2,4^. During these outbreaks, these viruses infected millions of Thai people and spread to virtually every part of the country^2^, including southern Thailand.

**Figure 1:**
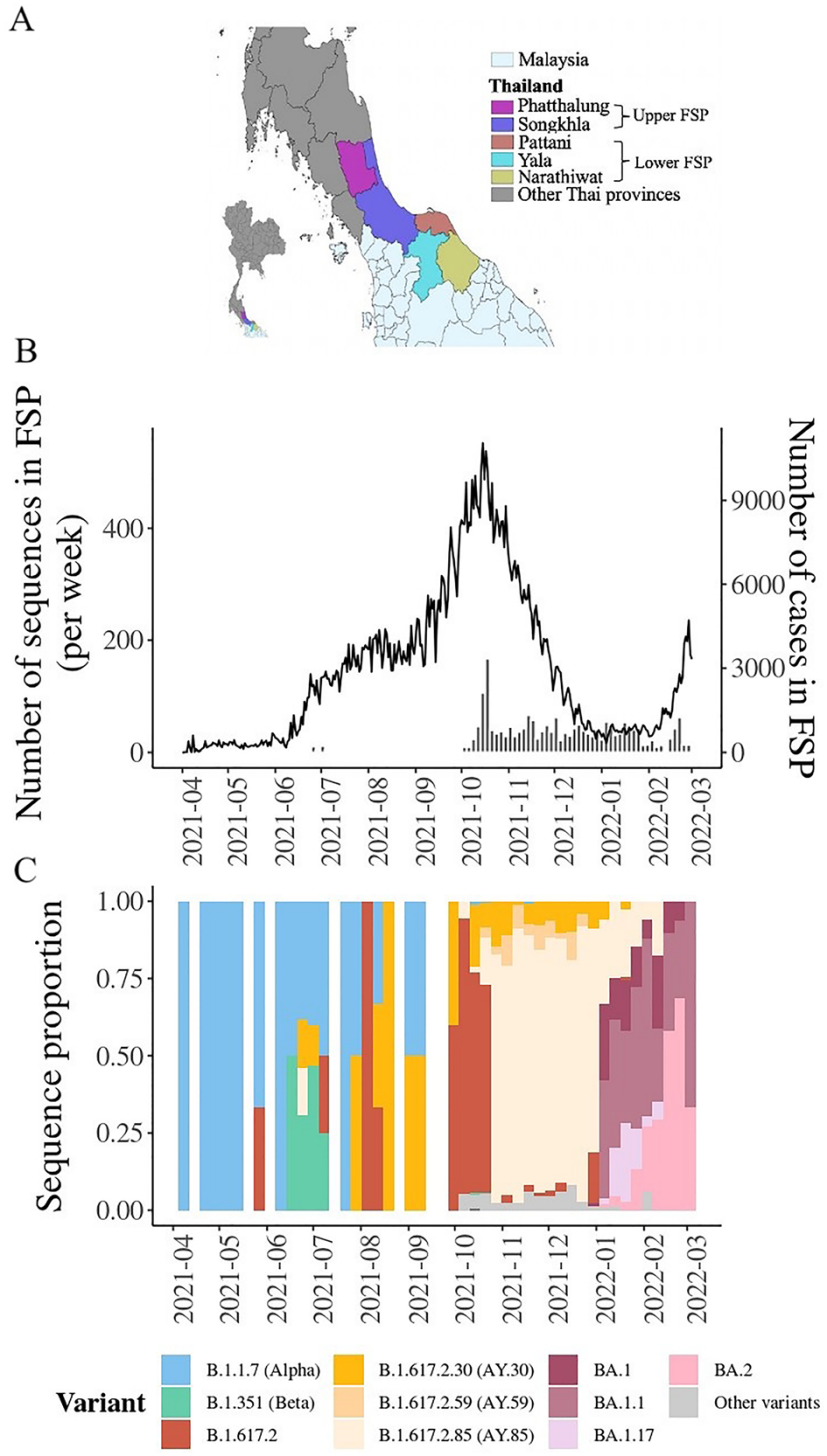
Overview of the 1942 SARS-CoV-2 whole-genome sequences generated in this study. (**A**) All virus samples were collected from the FSPs of Thailand. This area consists of Phatthalung (purple), Songkhla (blue), Yala (brown), Pattani (cyan), and Narathiwat (yellow). In this study, we tentatively grouped these provinces into two regions according to their geographical locations and population demographics: the upper (Phatthalung and Songkhla), and lower (Yala, Pattani, and Narathiwat) FSP regions. (**B**) The number of confirmed COVID-19 cases as reported by The Ministry of Public Health of Thailand^6^ (line, right axis) and number of SARS-CoV-2 samples sequenced in this study (bars, left axis) over the study period. (**C**) Proportion of the sequences of various SARS-CoV-2 variants detected in this study over time. FSPs, five southernmost provinces; COVID-19, coronavirus disease-2019; SARS-CoV-2, severe acute respiratory syndrome coronavirus-2.

In this study, we aimed to explore the genetic diversity of SARS-CoV-2 during its highly active period from April 2021 to March 2022 in the five southernmost provinces (FSPs) of Thailand: Phatthalung, Songkhla, Yala, Pattani, and Narathiwat (Fig. 1A). This region forms part of the border between Thailand and Malaysia and is a popular tourist destination and culturally diverse area of the country. Based on their geographical location and population demographics, the area can be tentatively divided into the upper FSP region, which includes Phatthalung and Songkhla, mainly inhabited by Thai people of Chinese ancestry (Chinese-Thai), and the lower FSP region, which includes Yala, Pattani, and Narathiwat, predominantly inhabited by Muslim-Thai people. Despite the border checkpoints being closed during the outbreak, cross-border travel between the lower FSP and neighboring Malaysia remained common due to ethnic, cultural, and economic ties^5^. Therefore, this area was of particular interest, as it had the potential to receive viruses from both within the country and Malaysia.

Our analysis revealed evidence for transboundary virus transmissions in and out of the FSP region during the study period. Most of the collected samples were found to be of the B.1.617.2 and AY (Delta) variants, with the AY.85 (B.1.617.2.85) variant being the most abundant in our dataset. Spike protein mutations harbored by the AY.85 variant were characterized, and the mutation F306L was found to be common in our setting, which has not been previously associated with this variant. We conducted in silico structural analysis to examine the potential impact of the mutation on the properties of the spike protein. The potential impacts of the mutation on various aspects of the biological features of the virus, including viral load and fatality rate, were also investigated. Other potential co-variates, including patient age, sex, and geographical location, were examined and considered in the investigation.

## Results

### SARS-CoV-2 genome data

This study generated 1942 high-quality whole-genome sequences of SARS-CoV-2 samples obtained from the FSP area between April 2021 and March 2022, with most samples collected after October 2021 (Fig. 1B). Information regarding patient age, sex, geographical location, cycle threshold (Ct) values (viral load), and death status was also collected (Supplementary Table S1). The analysis revealed that our sequence collection was composed of sequences of B.1.1.7 (Alpha), B.1.351 (Beta), B.1.617.2 (classic Delta), and several AY variants, which are subvariants of the B.1.617.2 variant, as well as several BA (Omicron) variants. Figure 1C shows the relative frequency of the viral variants present in our sequence dataset over time.

To provide a more detailed account, of the 1942 sequences generated in this study, 1419 (73%) were identified to belong to the B.1.617.2 and AY (Delta) variants. Overall, we found that the AY.85 variant was the most predominant variant in our dataset (*n* = 840, 59.20% of the total Delta sequences detected), followed by the classic Delta B.1.617.2 variant (*n* = 323, 22.76%), AY.30 (*n* = 141, 9.94%), and AY.59 (*n* = 59, 4.16%) variants. Other Delta AY sublineages were also present in our sequence dataset; however, they represented less than 1% of the total samples and did not produce any large clusters. Most Delta sequences in our dataset were obtained between October and December 2021. During October 2021, the classic Delta B.1.617.2 sequences showed the highest frequency among all the detected Delta variants, but it was then rapidly surpassed by those of the variants AY.30, AY.59, and AY.85, starting from November 2021. The sequence frequencies of these three variants remained high until early January 2022, when they were subsequently superseded by those of the Omicron variants.

Our sequence dataset contained 446 Omicron sequences (23% of the total sequences), the majority of which were the BA.1 (*n* = 83, 18.61% of the total Omicron sequences), BA.1.1 (*n* = 185, 41.48%), BA.1.17 (*n* = 46, 10.31%), and BA.2 (*n* = 105, 23.54%) variants. Other BA variants constituted only 6.05% of the total Omicron sequences (*n* = 27). The frequencies of Omicron variants in our dataset increased rapidly in January 2022 and remained high until the end of the study in March 2022.

In addition, sequences of the Alpha (B.1.1.7) and Beta (B.1.351) variants were detected in our dataset most frequently between April 2021 and August 2021, prior to the Delta outbreaks. However, the absolute number of sequences in our dataset was low (B.1.1.7, *n* = 41; 2.10% of the total number of sequences in the dataset; B.1.351, *n* = 17; 0.87%). Therefore, we were unable to provide a comprehensive description of the diversity of these viral variants. All of these observed sequence temporal patterns were in line with official government reports investigating SARS-CoV-2 prevalence based on real-time polymerase chain reaction (RT-PCR) sequence detection analysis^7^.

### Phylogenetic analysis of SARS-CoV-2 in the FSP area

**Figure 2:**
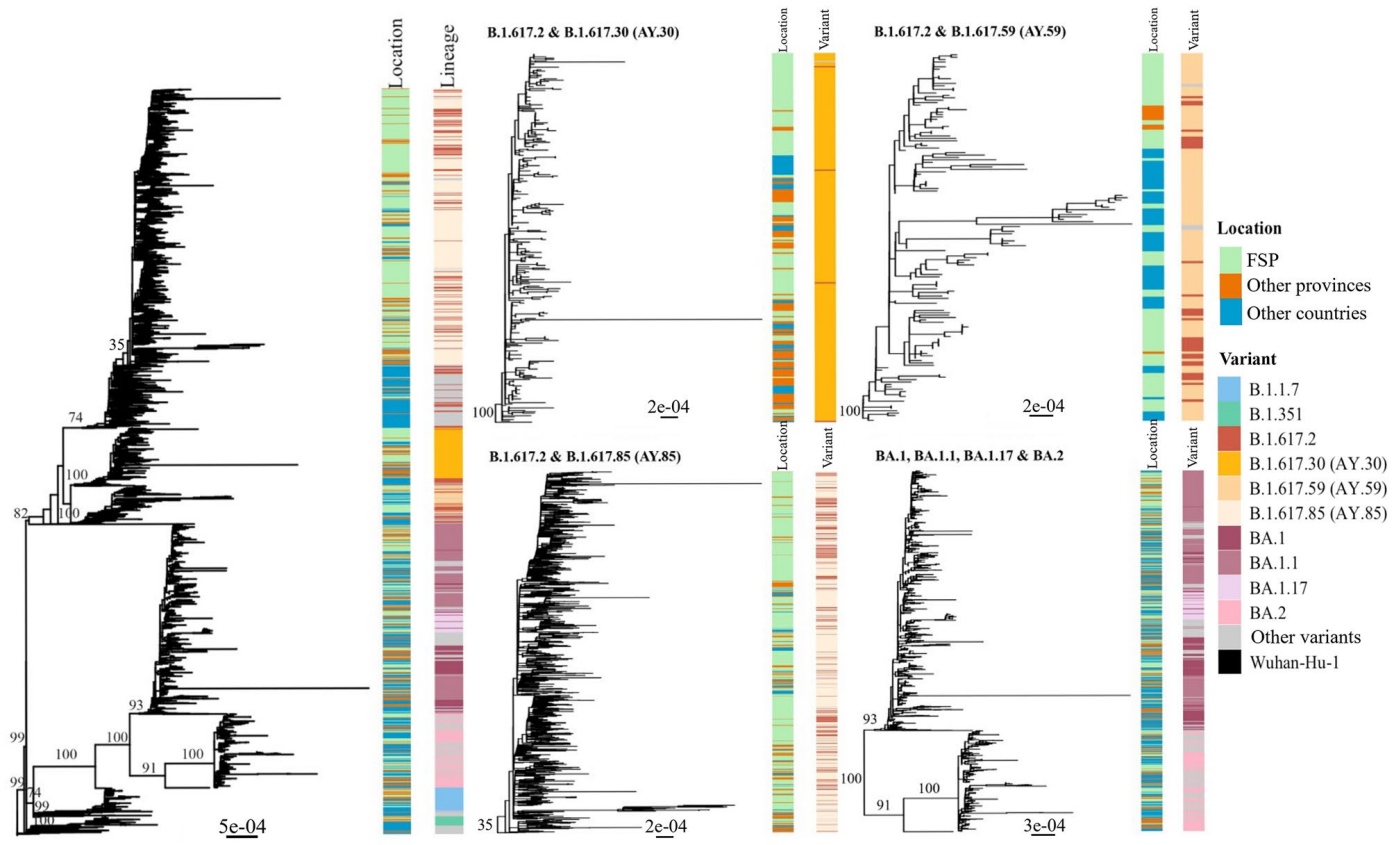
Maximum likelihood phylogenetic analysis of SARS-CoV-2 circulating in the FSP region. The analysis was carried out using IQ-TREE2 v.2.1.2^11^, with the nucleotide substitution model GTR + I + R5, which was determined as the best-fit model under the Bayesian information criterion. The analysis was supplemented with SARS-CoV-2 sequences from other regions in Thailand (*n* = 828) and other countries (*n* = 1391) that showed high similarity to those in the area (*n* = 1942). The tree was rooted with the Wuhan-Hu-1 isolate (NC_045612.2), one of the earliest SARS-CoV-2 samples isolated from a Chinese patient in December 2019. The scale bars are in the units of substitutions per site. The numbers on branches are bootstrap branch support values, computed based on 1000 pseudoreplicate datasets. Geographical locations and Pango lineage assignments of the viruses are shown by means of vertical color bars on the right of the tree (see keys). Individual phylogenetic clusters of the AY.30, AY.59, AY.85, and BA variants are depicted on the right-hand side.

Maximum likelihood phylogenetic analysis was performed to investigate the phylogenetic structures of SARS-CoV-2 within the FSP region (Fig. 2). The data were supplemented with sequences from Thailand but outside the FSP region (*n* = 828) and from outside the country (*n* = 1391), obtained from the Global Initiative on Sharing All Influenza Data (GISAID) database (See “Methods”).

Our analysis showed that the B.1.617.2/AY, BA, B.1.1.7, and B.1.351 variants formed their own distinct, well-supported clades (bootstrap support: B.1.617.2/AY, 82; BA, 100; B.1.1.7, 99; and B.1.351, 100). The BA variants showed a sister taxon relationship with the B.1.1.7 variant (bootstrap support: 74), as previously reported^8^. The three major AY Delta variants detected in our study formed distinct phylogenetic clusters with the classic Delta variant B.1.617.2 scattered across them (bootstrap support: AY.30, 100; AY.59, 100; and AY.85, 35). Similarly, the Omicron BA.1 and BA.2 variants also formed their own well-supported phylogenetic clusters (bootstrap support: BA.1, 93; and BA.2, 100), but all BA.1 subvariants (BA.1, BA.1.1, and BA.1.17) clustered together without showing a clear phylogenetic distinction.

Examination of the Delta and Omicron variant phylogenetic clusters revealed that the FSP sequences were grouped together with those collected from outside the area, including both Thailand and outside the country, without showing strong spatial phylogenetic structures (Fig. 2). This was consistent with that multiple virus transmissions occurred both in and out of the FSP region during the outbreaks. For example, we found that the AY.59 sequences from the FSP area clustered together with a considerable number of Malaysian sequences (Supplementary Fig. S1), thereby providing positive evidence for cross-country transmission of the virus during Delta outbreaks in the FSP region, despite strict international travel controls^9^. In addition, there were more non-Thai sequences in the Omicron variant cluster than in the Delta variant cluster, indicating a higher degree of cross-country transmission. This observation, however, may have been expected; since just before the global Omicron outbreaks began in early 2022, Thailand lifted its cross-country travel restrictions in November 2021^2,10^, thus offering some explanation for the observed pattern.

### F306L spike protein mutation in the AY.85 variant

Our analysis revealed that the AY.85 variant was the most frequently found variant in our dataset. This is in line with a previous report that Thailand was among the primary locations where this variant was detected worldwide^2,12^. Therefore, we analyzed their spike protein sequences in detail to better characterize and gain a better understanding of this virus.

**Figure 3:**
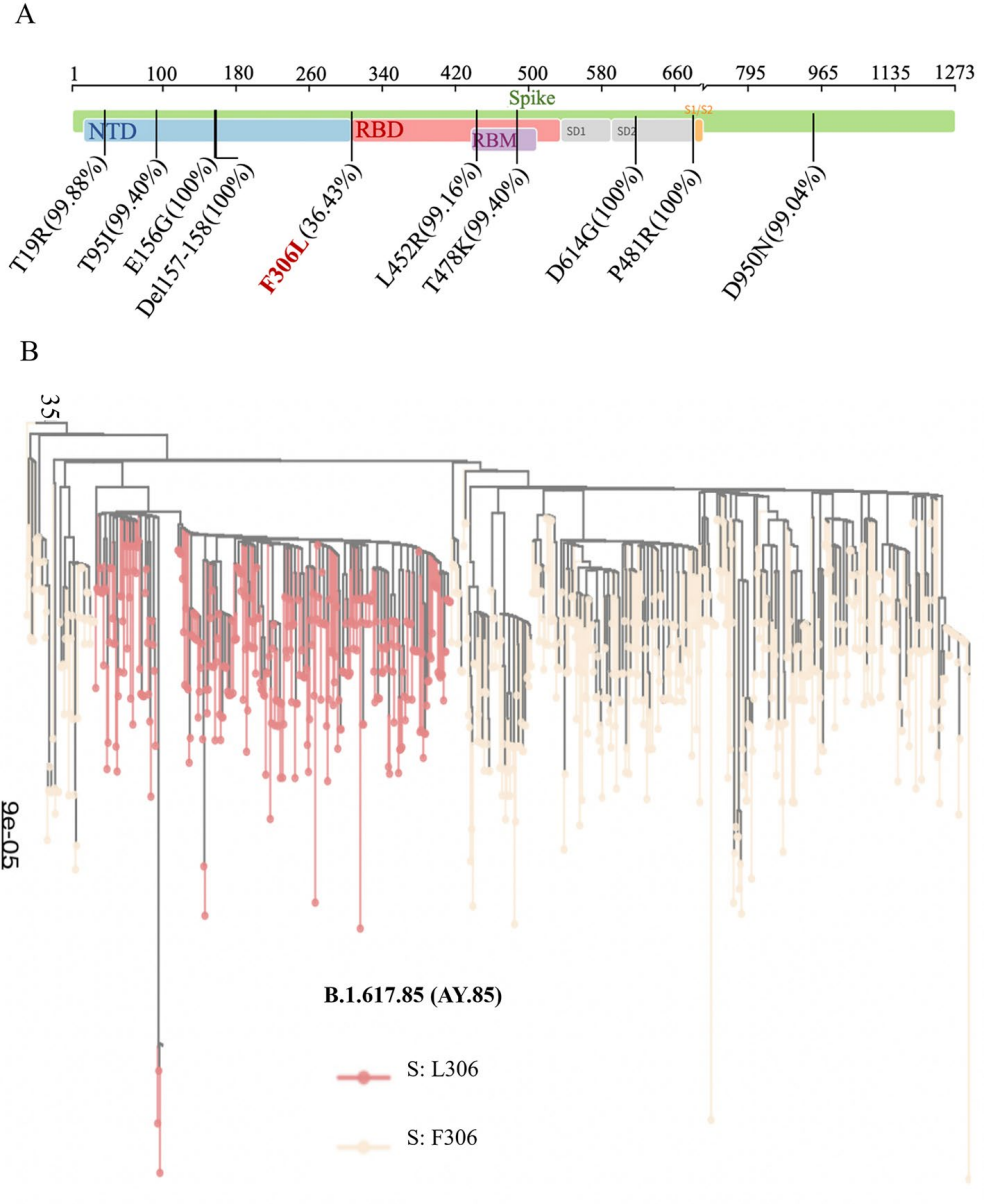
Mutations on the spike protein of the AY.85 variant in the FSP area. The mutations were identified by using the sequence of the Wuhun-Hu-1 isolate as the reference. (**A**) Our analysis detected a total of 10 mutations on the spike protein of the AY.85 variant at a frequency of over 90%, including T19R, T95I, E156G, del157/158, L452R, T478K, D614G, P681R, and D950N (written in black); all are known mutational markers of the AY.85 variant. Interestingly, our analysis detected the mutation F306L in 36% of our AY.85 sequences. The percentages in the figure indicate the frequency of those mutations found in our AY.85 sequences. (**B**) Phylogenetic analysis showed that AY.85-S: L306 forms a clade embedded within a larger phylogenetic cluster of AY.85-S: F306 and showed only sequences from the FSP (excluding those from other provinces and other countries).

Our analysis detected several mutations in the spike protein of the AY.85 variant circulating in the FSP area that occurred at a frequency of over 90% in comparison to the reference Wuhun-Hu-1 SARS-CoV-2 isolate (Fig. 3A). These mutations included T19R (*n* = 839, 99.88%), T95I (*n* = 835, 99.40%), E156G (*n* = 840, 100%), del157/158 (*n* = 840, 100%), L452R (*n* = 833, 99.16%), T478K (*n* = 835, 99.40%), D614G (*n* = 840, 100%), P681R (*n* = 840, 100%), and D950N (*n* = 832, 99.04%). All of these are known mutational markers of the AY.85 variant^12^. Interestingly, our analysis identified a spike protein mutation F306L in 36.43% (*n* = 306) of the AY.85 sequences examined in this study. This mutation is located in the boundary region between the N-terminal and receptor-binding domains of the protein (Fig. 3A) and has not previously been associated with this variant. Further analysis showed that AY.85 isolates with the F306L mutation (AY.85-S: L306) formed their own distinct phylogenetic cluster, and the cluster was inferred to be within a larger cluster of those without the mutation (AY.85-S: F306) (Fig. 3B). This was consistent with that the L306 variant is a mutant AY.85 variant that was able to successfully spread further in the human host population.

### Binding affinity of the spike proteins of AY.85-S: L306 and AY.85-S: F306 to the human cell receptor angiotensin-converting enzyme 2 (ACE2)

**Figure 4:**
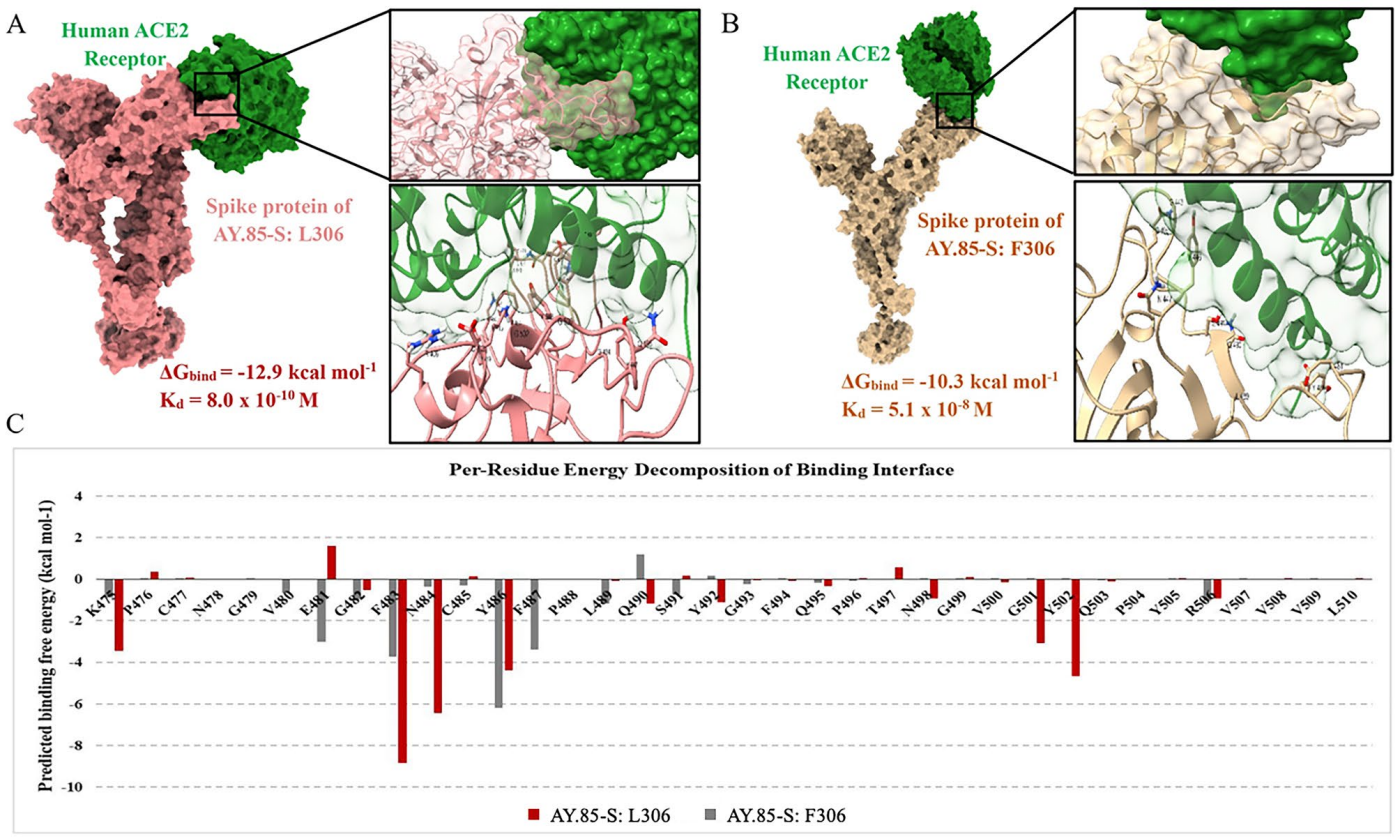
In silico protein–protein docking and binding interface analysis. (**A**) Predicted binding affinity and binding interaction between AY.85-S: L306’s spike protein (pink) and human ACE2 protein (green). (**B**) Predicted binding affinity and binding interaction between AY.85-S: F306L’s spike protein (deep yellow) and human ACE2 protein (green). (**C**) Predicted binding affinity of individual amino acid residues at the binding interface of the spike proteins of the two virus variants.

The F306L mutation is located next to the receptor-binding domain of the spike protein (Fig. 3A), which directly binds to the human host ACE2 cell receptor, which plays a crucial role in determining virus-cell specificity and the virus ability to enter host cells^13^. To investigate the potential impact of this mutation on the spike protein, we predicted the tertiary structures of the spike proteins with and without the mutation and performed in silico protein–protein docking analysis to assess whether the mutation alters the binding affinity of the protein to the human ACE2 cell receptor protein (Fig. 4). The Wuhan-Hu-1 spike protein was also analyzed for comparison.

The results suggested that both AY.85-S: F306 and AY.85-S: L306 spike proteins show a greater binding affinity to the human ACE2 protein than the Wuhan-Hu-1 spike protein, consistent a previous report that mutations in the spike proteins of variants of concern tend to increase the protein binding affinity to the human ACE2 receptor^14^. We also found that the spike protein of the AY.85-S: L306 variant has a greater binding affinity than that of the AY.85-S: F306 variant. Specifically, we found that the predicted binding free energy (∆G_bind_) value for the AY.85-S: L306 variant (− 12.9 kcal·mol^−1^) was lower than that for the AY.85-S: F306 variant (− 10.3 kcal·mol^−1^), and that of the Wuhan-Hu-1 variant was highest among the three (− 7.3 kcal·mol^−1^). Consistently, the predicted dissociation constant (K_d_) value for the AY.85-S: L306 variant (8 × 10^−10^ M) was lower than that for the AY.85-S: F306 variant (5.1 × 10^−8^ M), and that of the Wuhan-Hu-1 variant was the highest one (7.0 × 10^−6^ M). Our analysis also suggested that the F306L mutation allows for more amino acid residues of the host ACE2 protein to interact with the virus spike protein (16 residues: F484, S475, K476, N485, G474, A473, Y487, K456, F454, Y419, K415, Y447, G494, Y503, Q496, G500, G502, D403, and R406), as compared to that without (9 residues: G443, Q495, Y446, N447, S491, Q490, L489, Y486, and E481) (Fig. 4A–B). Additionally, the analysis revealed that the binding residues of the AY.85-S: L306 variant appeared to have more energetically favorable binding free energies than those of AY.85-S: F306 (Fig. 4C), in agreement with the predicted lower overall ∆G_bind_ obtained from the docking analysis. Taken together, our findings suggested that the spike protein of AY.85-S: L306 may induce tighter binding to the host receptor ACE2 protein.

### Viral loads did not differ significantly among patients infected with different Delta variants

We performed linear model regression analysis to investigate whether the viral loads differed significantly among patients infected with the AY.85-S: L306 and AY.85-S: F306 variants as well as the other two predominant Delta variants encountered in our dataset, AY.30 and AY.59. Other factors potentially affecting viral loads were also considered in the calculation, including patient sex (female *vs.* male), age (≤ 59 *vs.* > 59 years old), and geographical location (upper FSP *vs.* lower FSP). RT-PCR Ct values were used as a proxy for viral load, with lower Ct values indicating higher viral loads.

**Figure 5:**
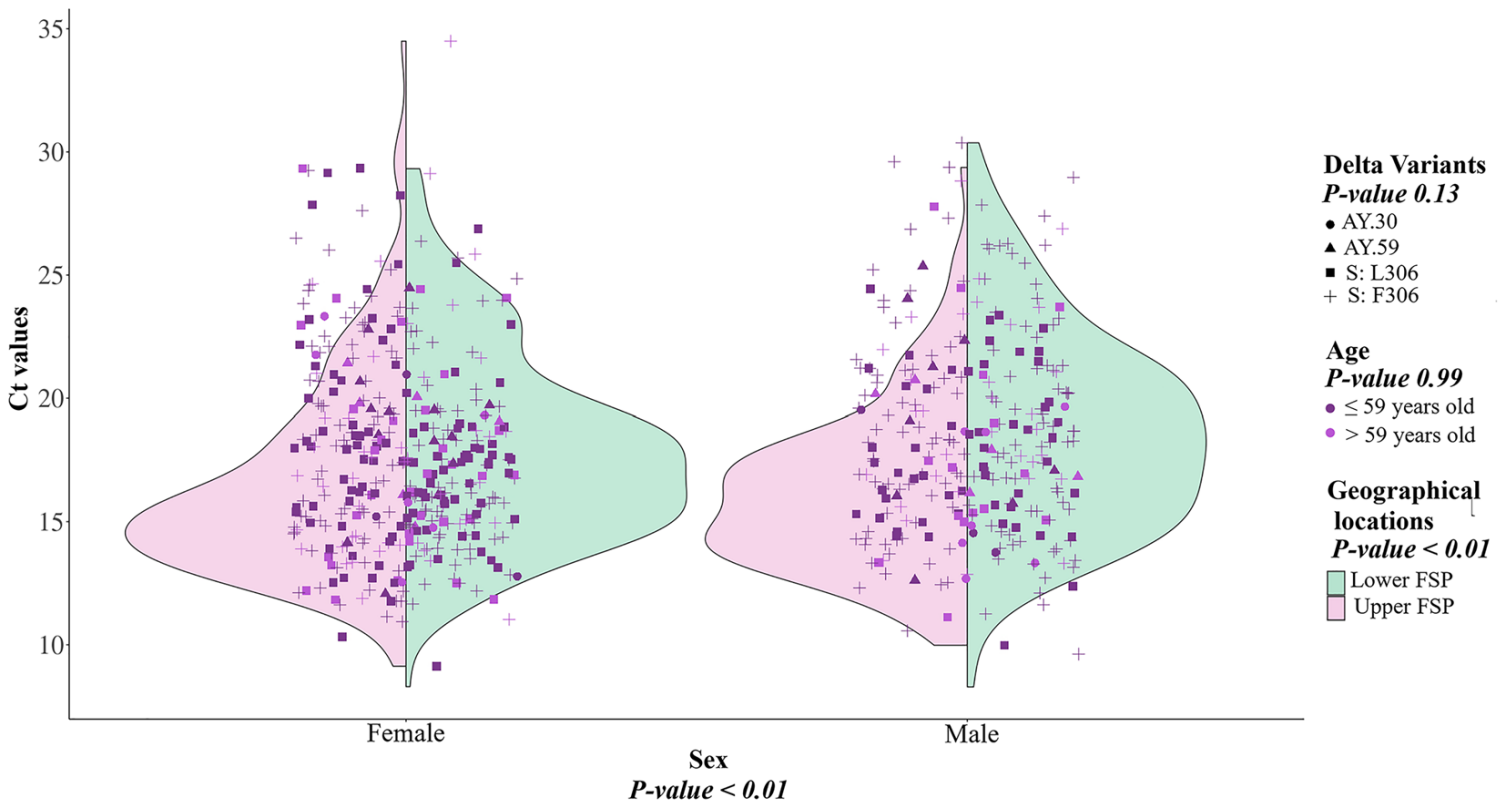
Correlation between the Ct value and age group, sex, virus variant, and geographical location. The significance of the correlation was assessed using linear model regression analysis. Other factors potentially affecting viral loads were also considered in the calculation, including patient sex (female *vs.* male), age (≤ 59 *vs.* > 59 years old), and geographical location (upper FSP *vs.* lower FSP). We found that the effects of geographical location and sex were significant (both *P* < 0.01), while those of age (*P* = 0.99) and virus variant (*P* = 0.13) were not.

The analysis (Fig. 5) suggested that the Ct values did not differ significantly among patients infected with different Delta variants (*P* = 0.13) or among patients of different age groups (*P* = 0.99), but differed significantly between males and females, with males having a significantly higher Ct value (*i.e.*, lower viral load) than females (*P* < 0.01). We also observed that the Ct value varied significantly between geographical locations (*P* < 0.01).

### F306L mutation is associated with an elevated fatality rate

During the study period, we found that among the 1417 patients infected with the Delta variants, 1.97% (*n* = 28) died. Among the Delta variants, AY.85 had the highest patient fatality rate (19/1417, 1.34%), whereas none of the patients infected with AY.59 (*n* = 59) and AY.30 (*n* = 141) died. Notably, patients infected with AY.85-S: L306 (16/840, 1.90%) had a higher crude fatality rate than those infected with AY.85-S: F306 (3/840, 0.35%). To statistically assess the impact of the F306L mutation on fatality rate, as well as other factors that could affect the rate, including age (≤ 59 *vs.* > 59 years old), sex (female *vs.* male), and geographical region (upper FSP *vs.* lower FSP), we conducted univariable and multivariable logistic regression analyses to estimate the crude odds ratios (CORs) and adjusted odds ratios.

(AORs) of fatality, respectively (Table 1, see Supplementary Table S2 for raw data).

**Table 1:** ORs of various potential risk factors for AY.85 infection fatality.

Variables	Level	Status		Crude OR		P	Adjusted OR		P
		Survived	Dead	OR	95% CI		OR	95% CI	
Total number (cases)		821 (97.7%)	19 (2.3%)						
Age	≤ 59 years	549 (99.3%)	4 (0.7%)	15.6	5.56–55.4	< 0.001	14.81	5.13–53.66	< 0.001
	> 59 years	132 (89.8%)	15 (10.2%)						
Sex	Male	344 (98.0%)	7 (2.0%)	0.81	0.30–2.03	0.66	0.84	0.30–2.47	0.75
	Female	477 (97.5%)	12 (2.5%)						
AY.85 variant	S: F306	531 (99.4%)	3 (0.6%)	9.77	3.22–42.2	< 0.001	9.26	2.92–41.20	< 0.001
	S: L306	290 (94.8%)	16 (5.6%)						
Province	Upper FSP	318 (99.7%)	1 (0.3%)	11.4	2.33–205	0.018	6.35	1.22–116.63	0.02
	Lower FSP	503 (96.5%)	18 (3.5%)						

*ORs* odds ratios.

In the univariable analysis, we found that an infection with AY.85-S: L306 was associated with a significant increased risk of fatality [COR = 9.77, 95% confidence interval (CI) = 3.22–42.2, *P* < 0.001], as was increasing age (COR = 15.6, 95% CI = 5.56–55.4, *P* < 0.001). The fatality rate was significantly higher in the lower FSP region than in the upper FSP region (COR = 11.4, 95% CI = 2.33–205, *P* = 0.018). The fatality rate was estimated to be lower in males than in females, but not significant (COR = 0.81, 95% CI = 0.30–2.03, *P* = 0.66).

The same results were obtained using multivariable logistic regression analysis, which computed the odds ratios of all factors simultaneously. The positive association between the fatality with the F306L mutation (AOR = 9.26, 95% CI = 2.92–41.20, *P* < 0.001), increasing patient age (AOR = 14.81, 95% CI = 5.13–53.66, *P* < 0.001), and lower FSP (AOR = 6.35, 95% CI = 1.22–116.63, *P* = 0.02) remained significant, while the analysis did not detect a significant difference in the fatality rate between males and females (AOR for males compared to females = 0.84, 95% CI = 0.40–3.30, *P* = 0.75).

## Discussion

This study explored the genetic diversity of SARS-CoV-2 in the FSP area of Thailand during its highly active period from April 2021 to March 2022. We collected and sequenced 1942 samples of SARS-CoV-2 from the area and analyzed their whole-genome sequences to investigate their relationship with viruses from outside the area. We also examined whether different viral variants were associated with varying viral loads and fatality rates. Patient age, sex, and geographical location were also considered in the analyses.

Our analysis revealed that the majority of viruses in our dataset were Delta B.1.617.2 and AY (1419, 73%) variants, most notably the classic Delta B.1.617.2, AY.30, AY.59, and AY.85 variants. During the nationwide outbreaks of the virus (June 2021–November 2021), strict public health measures, including city and country lockdowns and isolation of infected persons, were implemented throughout the country to control the spread of the virus^2,3^. Despite this, our analysis identified a considerably high number of isolates from outside the FSP area, both within and outside the country, to show high similarity to the FSP isolates and form mixed phylogenetic clusters together in the phylogeny (Fig. 2), suggesting cross-provincial and -country transmission of the virus. For instance, we found that almost all non-Thai isolates in the AY.59 cluster were from Malaysia and they were phylogenetically indistinguishable from the FSP isolates (Supplementary Fig. S1), indicating multiple viral exchanges between the two areas. Despite the closure of border checkpoints during the outbreak, new sources had reported that people continued to travel between Thailand and Malaysia through natural channels^15^. This could be one of the routes through which the virus spread between the two countries, potentially explaining our observations.

Compared with the Delta B.1.617.2 and AY variants, we observed considerably more Omicron isolates from outside the FSP region that showed high similarity to those in the area, and they formed a large phylogenetic cluster together without showing any clear spatial structure on the tree (Fig. 2). This suggested that the outbreaks of Omicron variants in the FSP region (January–March 2022) were caused by and supplemented with a greater number of virus introductions from multiple geographical sources, both outside and within the country. The relaxation of public disease control by the Thai government starting in November 2021 just before the nationwide Omicron wave^2,16^ may explain this observation, at least in part.

In our sequence data collection, the AY.85 variant was the most frequently observed variant (*n* = 840/1942 = 43.25%), and we identified the spike protein mutation F306L as a common mutation of the virus in the FSP area (*n* = 306/840, 36.43% of the AY.85 sequences) (Fig. 3). To the best of our knowledge, this mutation has not been previously associated with this viral variant. Spike protein plays a critical role in virus cell specificity and cell entry, as it directly binds to the human host cell receptor ACE2^13,17,18^, and mutations in this protein have been reported to affect virus cell entry mechanisms, transmission, infectivity, pathogenesis, and ability to evade host immune systems^19–23^. The F306L mutation is located very close to the receptor-binding domain, and our in silico analysis suggested that it likely increases binding affinity to the host receptor ACE2 (Fig. 4). A previous study suggested that mutations that increase the binding affinity of the spike protein to ACE2 can result in changes in viral properties, including higher transmissibility and infectivity^22^. To this end, we explored whether the F306L mutation affects the biological properties of the virus by investigating whether the viral load and fatality rate differed significantly among patients infected with the AY.85-S: L306 and AY.85-S: F306 variants. Several other potential co-variates including patient sex, age, and geographical location were also considered in the analysis.

Our analysis did not detect significant differences in viral loads among patients infected with the two viral variants (and other Delta variants) or among patients of different age groups (Fig. 5). However, we observed a significantly lower viral load in males than in females (Fig. 5). These findings were consistent with those of previous studies. For instance, a study conducted in Saudi Arabia reported no significant difference in viral loads between age groups and a significantly lower viral load in males^24^ similar to ours, and a large-scale study conducted in the USA detected no significant difference in viral loads between children and adults^25^. However, several studies have reported different results. For example, a study conducted in the USA reported a significantly higher viral load in males^26^, and the same trend was observed in Italy, although it was found that males had a faster viral load decay than females, and the viral load decayed more slowly in older patients^27^. Studies from Australia^28^ and Turkey^29^, on the other hand, reported no significant difference in viral loads among patients of different sexes and age groups. This suggests that the trends may vary from setting to setting, and the association between viral load dynamics and age or sex remains highly debated. Indeed, our study also detected a significant difference in viral loads among patients from the upper and lower FSP areas (Fig. 5), mirroring these disparities.

With respect to the fatality rate, we found that the rate increased with age, consistent with previous results^30^; however, the rate was not significantly different between males and females in our setting (Table 1). In contrast, several large-scale studies have reported higher fatality rates from SARS-CoV-2 infection in males, as compared to that in females, and this appears to be a worldwide phenomenon^31–38^. A wide range of behavioral and biological factors have been proposed as plausible (non-mutually exclusive) explanations for the sex disparities in COVID-19 severity and outcomes, including sex differences in social, cultural, hygiene, and economic behaviors; differential prevalence of medical conditions associated with a higher risk of COVID-19 fatality, such as diabetes, hypertension, chronic cardiovascular disease, and respiratory disease; and effects of sex hormones on, for instance, virus entry and priming, as well as immune and inflammatory responses^31,33^. Nevertheless, it should be noted that the degree of sex differences in COVID-19 fatalities differed widely among countries^35,36^, and similar to the present study, some studies found no differential COVID-19 fatalities between sexes. For example, a study of an Italian cohort showed that death rates were not significantly different between males and females after adjusting for various confounding factors, including age and disease severity at hospital presentation^39^. The opposite trend has also been observed in a few countries such as India, Iraq, Bhutan, and Singapore^40,41^. This could be due to incomplete data, systematic biases in case identification by sex, and/or a reflection of actual differences in healthcare systems, public health control measures, vaccination coverage, health-seeking behaviors, and access to care among males and females across geographical regions^31,36,40^.

In parallel, our study found a significant difference in COVID-19 fatality rates between the upper and lower FSP regions (Table 1). The southern part of Thailand, especially the border area, is a distinctive area of the country with a variety of cultures and beliefs, and it was challenging to fully control the health behaviors and transborder mobility of the people between this area and northern Malaysia. In the early stages of the nationwide vaccination program, the lower FSP area saw a slow distribution of COVID-19 vaccines due to certain beliefs of the locals^42^; however, this was later improved by effective public communication by local scholars. A survey conducted in October and November 2021 revealed that some immigrant workers from Myanmar in this area were reluctant to receive COVID-19 vaccination due to a lack of information about vaccine efficacy and side effects^43^. The delay in vaccine administration may, at least in part, explain the higher case fatality rate observed in the lower FSP group.

After adjusting for age, sex, and geographical location, patients infected with the AY.85-S: L306 variant were found to have a significantly higher odds of fatality than those infected with the AY.85-S: F306 variant (Table 1). Our in silico structural analysis suggested that the mutant spike protein likely binds to the host ACE2 cell receptor more tightly (Fig. 4); however, how this molecular feature contributes to the observed increased fatality rate (if at all) is unclear, and further experimental investigations are needed to elucidate the molecular mechanisms underlying this association. Interestingly, by searching for the mutation on the GISAID database, we found that, while its absolute frequency was low (detected in 2622 complete genome sequences out of 15,438,334 sequences = 0.017%, access date: April 20, 2023), this mutation was found to be geographically widespread, occurring in sequences reported from many countries, most notably, the USA (*n* = 611/2622, 23.30%), Thailand (*n* = 511, 19.49%), Russia (*n* = 460, 17.54%), United Kingdom (*n* = 328, 12.51%), and Germany (*n* = 191, 7.28%). Furthermore, this mutation could also be observed in many other virus variants besides AY.85 (*n* = 506, 19.30%), including B.1.1.523 (*n* = 611, 23.03%), B.1.1.7 (*n* = 154, 5.87%), many B.1.617.2 and AY variants (*n* = 1011, 38.56%), and many BA variants (*n* = 529, 20.18%). This observation suggested that this particular mutation may be of great interest and highlights it as a frequent convergent evolutionary feature of SARS-CoV-2. Thus, there is a need for more studies to monitor this mutation and further investigate its effects on viral pathogenesis and epidemiology.

## Materials and methods

### SARS-CoV-2 sample collection

Nasopharyngeal and throat swab samples were collected from COVID-19 patients by the Health Science Center District 12 of the FSP area. Samples from the first period of the study (April 2021–September 2021) were obtained from randomly selected patients at a university hospital in Songkhla (*n* = 74). In collaboration with the Ministry of Public Health of Thailand, the study later received a second larger set of samples from the District 12 Medical Science Center between October 2021 and March 2022 (*n* = 1868). All samples were tested positive for SARS-CoV-2 by means of RT-PCR using one of the following commercial assays: SARS-CoV2 (N/ORF) (Sansure) or an in-house protocol following the US Centers for Disease Control and Prevention guidelines (https://www.fda.gov/media/134922/download). Metadata, including patient age, sex, province, and dead/alive status, were collected within three days of the onset of symptoms, and the patients were followed up until they were discharged or died from SARS-CoV-2 infection (Supplementary Tables S1 and S2). The amount of viral genetic material present in the samples was assessed using RT-PCR and reported as the Ct value.

### SARS-CoV-2 sequencing

The SARS-CoV-2 positive samples (*n* = 1942) were sequenced using an Illumina sequencing machine (COVIDSeq™ Test). Illumina® DRAGEN COVID Lineage App (DRAGEN DNA pipeline version 3.5.7) was used to perform K_mer_-based detection of SARS-CoV-2. The viral reads obtained were aligned to the Wuhun-Hu-1 reference genome (NC_045512.2) to call nucleotide variants and generate consensus genome sequences. The Pango lineage assignment was performed using Pangolin (https://pangolin.cog-uk.io/) and NextClade (https://clades.nextstrain.org/).

### Maximum likelihood phylogenetic analysis

Multiple sequence alignment of the 1942 whole-genome sequences of SARS-CoV-2 from the FSPs was made using MAFFT v.7.475^44^, supplemented with sequences similar to those in the area, but from other countries (*n* = 1392) and Thailand, but outside the FSP area (*n* = 904; see below). The phylogeny was reconstructed under the maximum likelihood framework using IQ-TREE v.2.1.2^11^. GTR + F + R5 was determined to be the best-fit nucleotide substitution model under the Bayesian information criterion using ModelFinder^45^ and was used in the analysis. Branch support was assessed using the ultrafast bootstrap approximation method^46^ implemented in IQ-TREE v.2.1.2 with 1000 pseudo-replicates.

### Compilation of whole genome sequences of SARS-CoV-2 from outside Thailand and other provinces of Thailand for phylogenetic analysis

To compile a dataset of whole-genome sequences of SARS-CoV-2 from other countries and other provinces of Thailand outside the FSP area that showed high similarity to those in the FSP area for phylogenetic analysis (see above), we first downloaded the entire GISAID database of whole-genome sequences of SARS-CoV-2 on July 11, 2022, and excluded low-quality sequences (containing more than 5% ambiguous bases) and incomplete sequences (length < 29 kb) from the sequence collection. The dataset was then separated into two: a global non-Thai sequence collection and a Thai sequence collection, excluding those from the FSP area. We subsequently identified the top five most similar sequences for each FSP sequence in both sequence collections using BLASTn under default settings. A total of 1392 non-redundant sequences from other countries and 904 non-redundant non-FSP Thai sequences were determined to exhibit high similarity to those within the FSP area and were used to supplement the phylogenetic analysis of SARS-CoV-2 in the FSP area (see above). Pangolin v2.2.2 was used to determine the Pango lineage of these sequences^47^.

### Protein tertiary structural modeling

Tertiary structures of the spike proteins of AY.85-S: L306 and AY.85-S: F306 were predicted using the I-TASSER MTD online server^48^. The SARS-CoV-2 Delta spike protein in complex with the human ACE2 protein was used as a template to guide the domain assembly (name: S-ACE2-C2a; Protein Data Bank accession number: 7w99; accession date: December 26, 2022)^49^, and the analysis generated up to five 3D structural models for each protein. The model with the highest template modeling score (TM-score) was considered the best-predicted model (AY.85-S: L306: TM-score = 0.64 ± 0.15; AY.85-S: F306: TM-score = 0.64 ± 0.15) and was used for further analysis. In the best-predicted spike protein models of AY.85-S: F306 and AY.85-S: L306, 70.1% and 71.0% of their amino acid residues were found to be in “most favorable” regions in the Ramachandran plots by PROCHECK (https://saves.mbi.ucla.edu/)^50^, while 2.5% and 2.9% were in “disallowed” regions, respectively. The rest were in either “additionally allowed” or “generously allowed” regions (Supplementary Fig. S2).

### Molecular docking simulation

Molecular docking between the predicted spike protein structures of AY.85-S: L306 and AY.85-S: F306 and human ACE2 protein (Protein Data Bank accession number: 7w99) was performed using HADDOCK 2.4 (https://wenmr.science.uu.nl/haddock2.4/)^51,52^ under default settings, and with all COVID-19-related options selected (Supplementary Table S3). Both spike proteins were treated as ligands, whereas ACE2 was treated as a receptor. The number of structures was set to 1000 for rigid body docking, 200 for semi-flexible refinement, and 200 for explicit solvent refinement. This analysis was also performed with the reference Wuhan-Hu-1 spike protein as a validation control (Supplementary Note and Supplementary Fig. S3). The predicted Wuhan-Hu-1 spike protein/human ACE2 complex was highly similar to the one derived experimentally (structural alignment: root mean square deviation < 2 Å), validating the protocol (Supplementary Fig. S4).

### Binding free energy analysis

The binding affinities of the Wuhan-Hu-1, AY.85-S: L306 and AY.85-S: F306 spike proteins to the human ACE2 protein were assessed using PRODIGY (https://wenmr.science.uu.nl/prodigy/)^53^, which calculates the ∆G_bind_ and K_d_^54^. The AnalyseComplex function in FoldX^55^ was used to estimate the binding affinities of the complexes. Per-residue energy decomposition analysis was performed to compute the contribution of each individual amino acid residue to the total ∆G_bind_ using a fast Fourier transform-based method implemented in the pyDockEneRes server^56^.

### Association between F306L mutation and Ct value

We performed linear additive model regression analysis to investigate the association between Ct value and various variables, including virus variants (AY.85-S: L306, AY.85-S: F306, AY.30, and AY.59), patient age (≤ 59 and > 59 years), sex (male and female), and geographical location (upper and lower FSP). The analysis was carried out using the function “lm” in the R software 4.1.1 (http://www.R-project.org).

### Association between F306L mutation and case fatality rate

Both univariable and multivariable logistic additive regression analyses were carried out to compute CORs and AORs of fatality for the different virus variants (AY.85-S: L306 and AY.85-S: F306), patient ages (≤ 59 and > 59 years), sexes (male and female), and geographical locations (upper and lower FSP). Analyses were performed using the generalized linear model (glm) function in R.

Finally, we confirm that all methods were carried out in accordance with relevant guidelines and regulations.

### Ethics approval

The study has obtained informed consent from all patient subjects regarding the utilization of leftover clinical specimens for microbial genotyping. This research has been approved by the Human Research Ethics Committee of the Faculty of Medicine, Prince of Songkla University (REC.63-246-5-2). The use of anonymized clinical data without re-consent for research purposes has also been approved by the Human Research Ethics Committee.

### Supplementary Information

Supplementary Information.

## Data Availability

All SARS-CoV-2 genomes generated in this study are publicly available in the GISAID database (https://www.gisaid.org) (accession numbers: COVPSU-00379–COVPSU-02262 and WGCV-04651–WGCV-05208).
