# Supplementary material for: SARS-CoV-2 variant with the spike protein mutation F306L in the southern border provinces of Thailand
Source: Sci Rep. 2024 Apr 2;14:7729. doi: 10.1038/s41598-024-56646-6 (PMC10987673; doi:10.1038/s41598-024-56646-6)
Supplement: Supplementary file 1 — Supplementary Information. [file 41598_2024_56646_MOESM1_ESM.docx]

SARS-CoV-2 variant with the spike protein mutation F306L in the southern border provinces of Thailand

Thanit Sila^1^, Smonrapat Surasombatpattana^1^, Songyos Rajborirug^2^, Wison Laochareonsuk^3,4^, Pongsakorn Choochuen^4,5^, Chanon Kongkamol^6^, Thammasin Ingviya^6^, Napat Prompat^7^, Surakameth Mahasirimongkol^8^, Surasak Sangkhathat^3,4*^, Pakorn Aiewsakun^9,10*^

^1^Department of Pathology, Faculty of Medicine, Prince of Songkla University, Hat Yai, Songkhla, 90110 Thailand

^2^Department of Epidemiology, Faculty of Medicine, Prince of Songkla University, Hat Yai, Songkhla, 90110 Thailand

^3^Division of Surgery, Faculty of Medicine, Prince of Songkla University, Hat Yai, Songkhla, 90110 Thailand

^4^Translational Medicine Research Center, Faculty of Medicine, Prince of Songkla University, Hat Yai, Songkhla, 90110 Thailand

^5^Department of Biomedical Sciences and Biomedical Engineering, Faculty of Medicine, Prince of Songkla University, Hat Yai, Songkhla, 90110 Thailand

^6^Department of Family Medicine and Preventive Medicine, Prince of Songkla University, Hat Yai, Songkhla, 90110 Thailand

^7^Medical of Technology Service Center, Faculty of Medical Technology, Prince of Songkla University, Songkhla, 90110 Thailand

^8^Genetics Center, Medical Life Sciences Institute, Department of Medical Sciences, Ministry of Public Health, Nonthaburi, 11000 Thailand

^9^Pornchai Matangkasombut Center for Microbial Genomics, Department of Microbiology, Faculty of Science, Mahidol University, Bangkok, 10400 Thailand

^10^Department of Microbiology, Faculty of Science, Mahidol University, Bangkok, 10400 Thailand

*Surasak Sangkhathat and Pakorn Aiewsakun are co-corresponding authors.

Correspondence addresses

Surasak Sangkhathat, M.D., Ph.D.

Translational Medicine Research Center,

Faculty of Medicine, Prince of Songkla University,

Hat Yai, Songkhla, 90110 Thailand

E-mail: surasak.sa@psu.ac.th

Pakorn Aiewsakun

Department of Microbiology

Faculty of Science, Mahidol University,

Bangkok, 10400 Thailand

E-mail: pakorn.aie@mahidol.ac.th

# Supplementary Tables

**Supplementary Table S1** Metadata of sequences from the FSP area, consisting of sequence name, patient sex, province, sample collection date, age, Ct value (N gene) and Pango lineage.

| **Sequence name** | **Sex** | **Provinces** | **Collection date** | **Age** | **Ct value** | **Pango lineage** |
| --- | --- | --- | --- | --- | --- | --- |
| COVPSU-00379 | male | Narathiwat | 19/10/2021 | 57 | 20.67 | B.1.351 |
| COVPSU-00380 | female | Narathiwat | 19/10/2021 | 55 | 14.57 | AY.85 |
| COVPSU-00381 | male | Narathiwat | 19/10/2021 | 66 | 20.17 | AY.59 |
| COVPSU-00382 | female | Narathiwat | 19/10/2021 | 69 | 17.83 | AY.85 |
| COVPSU-00383 | female | Narathiwat | 19/10/2021 | 17 | 18.5 | AY.85 |
| COVPSU-00384 | male | Narathiwat | 20/10/2021 | 56 | 15.68 | AY.85 |
| COVPSU-00385 | male | Narathiwat | 20/10/2021 | 15 | 16.97 | AY.85 |
| COVPSU-00386 | female | Narathiwat | 20/10/2021 | 51 | 17.59 | AY.85 |
| COVPSU-00387 | female | Narathiwat | 20/10/2021 | 29 | 15.39 | AY.85 |
| COVPSU-00388 | female | Narathiwat | 20/10/2021 | 43 | 15.03 | AY.85 |
| COVPSU-00389 | female | Narathiwat | 20/10/2021 | 35 | 18.45 | AY.59 |
| COVPSU-00390 | male | Narathiwat | 20/10/2021 | 56 | 26.27 | AY.85 |
| COVPSU-00391 | male | Narathiwat | 20/10/2021 | 24 | 23.37 | AY.85 |
| COVPSU-00392 | female | Narathiwat | 20/10/2021 | 85 | 17.31 | AY.85 |
| COVPSU-00393 | male | Narathiwat | 9/10/2021 | 39 | NA | AY.85 |
| COVPSU-00394 | male | Narathiwat | 20/10/2021 | 55 | 24.36 | AY.85 |
| COVPSU-00395 | male | Narathiwat | 21/10/2021 | 19 | 25.48 | AY.85 |
| COVPSU-00396 | female | Narathiwat | 21/10/2021 | 69 | 14.78 | AY.59 |
| COVPSU-00397 | female | Narathiwat | 20/10/2021 | 19 | 20.36 | AY.85 |
| COVPSU-00398 | male | Narathiwat | 21/10/2021 | 15 | 22.47 | AY.85 |
| COVPSU-00399 | male | Narathiwat | 21/10/2021 | 26 | 21.55 | AY.86 |
| COVPSU-00400 | male | Narathiwat | 21/10/2021 | 66 | 17.92 | AY.59 |
| COVPSU-00401 | female | Narathiwat | 4/10/2021 | 34 | NA | AY.85 |
| COVPSU-00402 | male | Narathiwat | 22/10/2021 | 42 | 21.21 | AY.85 |
| COVPSU-00403 | male | Narathiwat | 24/10/2021 | 43 | 26.21 | AY.85 |
| COVPSU-00404 | female | Narathiwat | 24/10/2021 | 32 | 13 | AY.85 |
| COVPSU-00405 | male | Narathiwat | 24/10/2021 | 40 | 20.43 | AY.85 |
| COVPSU-00406 | female | Narathiwat | 24/10/2021 | 62 | 14.14 | AY.85 |
| COVPSU-00407 | female | Narathiwat | 25/10/2021 | 26 | 17.5 | AY.85 |
| COVPSU-00408 | male | Narathiwat | 25/10/2021 | 60 | 15.04 | AY.85 |
| COVPSU-00409 | male | Narathiwat | 25/10/2021 | 20 | 22.35 | AY.59 |
| COVPSU-00410 | female | Narathiwat | 25/10/2021 | 73 | 14.05 | AY.85 |
| COVPSU-00411 | female | Narathiwat | 25/10/2021 | 57 | 16.39 | AY.85 |
| COVPSU-00412 | male | Narathiwat | 25/10/2021 | 61 | 16.48 | AY.79 |
| COVPSU-00413 | female | Narathiwat | 25/10/2021 | 51 | 23.65 | AY.85 |
| COVPSU-00414 | female | Narathiwat | 25/10/2021 | 34 | 21.85 | AY.85 |
| COVPSU-00415 | female | Narathiwat | 25/10/2021 | 49 | 22.36 | AY.85 |
| COVPSU-00416 | female | Narathiwat | 25/10/2021 | 23 | 23.27 | AY.85 |
| COVPSU-00417 | female | Narathiwat | 25/10/2021 | 12 | 21.07 | AY.85 |
| COVPSU-00418 | male | Narathiwat | 25/10/2021 | 60 | 20.95 | AY.85 |
| COVPSU-00419 | female | Narathiwat | 25/10/2021 | 72 | 15.05 | AY.85 |
| COVPSU-00420 | female | Narathiwat | 26/10/2021 | 39 | 14.96 | AY.85 |
| COVPSU-00421 | male | Yala | 26/10/2021 | 41 | 19.6 | AY.85 |
| COVPSU-00422 | female | Narathiwat | 26/10/2021 | 34 | 22.1 | AY.85 |
| COVPSU-00423 | male | Narathiwat | 26/10/2021 | 40 | 23.33 | AY.30 |
| COVPSU-00424 | female | Narathiwat | 26/10/2021 | 60 | 13.8 | AY.85 |
| COVPSU-00425 | male | Narathiwat | 26/10/2021 | 59 | 15.94 | AY.85 |
| COVPSU-00426 | male | Narathiwat | 26/10/2021 | 42 | 19.55 | AY.85 |
| COVPSU-00427 | male | Narathiwat | 26/10/2021 | 41 | 20.07 | AY.85 |
| COVPSU-00428 | female | Narathiwat | 26/10/2021 | 30 | 16.77 | AY.85 |
| COVPSU-00429 | female | Narathiwat | 28/10/2021 | 64 | 22.97 | AY.85 |
| COVPSU-00430 | female | Narathiwat | 28/10/2021 | 26 | 18.8 | AY.85 |
| COVPSU-00431 | female | Narathiwat | 28/10/2021 | 35 | 19.45 | AY.59 |
| COVPSU-00432 | female | Narathiwat | 28/10/2021 | 30 | 17.05 | AY.85 |
| COVPSU-00433 | male | Narathiwat | 28/10/2021 | 49 | 20.88 | AY.85 |
| COVPSU-00434 | female | Narathiwat | 28/10/2021 | 40 | 13.61 | AY.85 |
| COVPSU-00435 | female | Narathiwat | 28/10/2021 | 60 | 21.01 | AY.85 |
| COVPSU-00436 | female | Narathiwat | 15/10/2021 | 36 | NA | B.1.617.2 |
| COVPSU-00437 | male | Narathiwat | 29/10/2021 | 58 | 14.3 | AY.85 |
| COVPSU-00438 | female | Narathiwat | 29/10/2021 | 36 | 19.26 | AY.85 |
| COVPSU-00439 | female | Narathiwat | 29/10/2021 | 79 | 25.56 | AY.85 |
| COVPSU-00440 | male | Narathiwat | 29/10/2021 | 23 | 16.74 | AY.85 |
| COVPSU-00441 | male | Narathiwat | 8/10/2021 | 37 | NA | B.1.617.2 |
| COVPSU-00442 | female | Narathiwat | 29/10/2021 | 71 | 14.95 | AY.85 |
| COVPSU-00443 | female | Narathiwat | 29/10/2021 | 61 | 13.98 | AY.85 |
| COVPSU-00444 | female | Narathiwat | 29/10/2021 | 13 | 19.66 | AY.85 |
| COVPSU-00445 | female | Narathiwat | 29/10/2021 | 42 | 22.81 | AY.85 |
| COVPSU-00446 | male | Narathiwat | 29/10/2021 | 58 | 21.76 | AY.30 |
| COVPSU-00447 | male | Narathiwat | 29/10/2021 | 46 | 18.68 | AY.85 |
| COVPSU-00448 | male | Narathiwat | 29/10/2021 | 39 | 26.24 | AY.85 |
| COVPSU-00449 | male | Narathiwat | 29/10/2021 | 90 | 13.31 | AY.85 |
| COVPSU-00450 | female | Narathiwat | 31/10/2021 | NA | 24.07 | AY.85 |
| COVPSU-00451 | female | Narathiwat | 1/11/2021 | NA | 20.16 | AY.59 |
| COVPSU-00452 | female | Narathiwat | 1/11/2021 | NA | 23.33 | AY.85 |
| COVPSU-00453 | female | Narathiwat | 1/11/2021 | NA | 23.78 | AY.85 |
| COVPSU-00454 | female | Narathiwat | 1/11/2021 | NA | 23.67 | AY.85 |
| COVPSU-00455 | male | Phatthalung | 1/11/2021 | NA | 16.35 | AY.85 |
| COVPSU-00456 | female | Narathiwat | 20/10/2021 | NA | 23.95 | AY.85 |
| COVPSU-00457 | male | Narathiwat | 1/11/2021 | NA | 23.3 | AY.85 |
| COVPSU-00458 | female | Yala | 1/11/2021 | NA | 22.69 | AY.59 |
| COVPSU-00459 | male | Phatthalung | 1/11/2021 | NA | 23.69 | AY.85 |
| COVPSU-00460 | female | Narathiwat | 1/11/2021 | NA | 17.66 | AY.85 |
| COVPSU-00461 | male | Narathiwat | 1/11/2021 | NA | 15.67 | AY.85 |
| COVPSU-00462 | male | Narathiwat | 1/11/2021 | NA | 22.99 | AY.85 |
| COVPSU-00463 | male | Narathiwat | 1/11/2021 | NA | 21.9 | AY.85 |
| COVPSU-00464 | male | Narathiwat | 1/11/2021 | NA | 24.75 | AY.85 |
| COVPSU-00465 | female | Phatthalung | 1/11/2021 | NA | 17.4 | AY.85 |
| COVPSU-00466 | female | Narathiwat | 16/11/2021 | NA | NA | AY.29.1 |
| COVPSU-00467 | male | Narathiwat | 1/11/2021 | NA | 19.77 | B.1.617.2 |
| COVPSU-00468 | male | Narathiwat | 1/11/2021 | NA | 26.88 | AY.85 |
| COVPSU-00469 | female | Narathiwat | 1/11/2021 | NA | 16.17 | AY.85 |
| COVPSU-00470 | female | Narathiwat | 3/11/2021 | NA | 13.82 | AY.85 |
| COVPSU-00471 | female | Narathiwat | 3/11/2021 | NA | 14.35 | AY.85 |
| COVPSU-00472 | male | Narathiwat | 3/11/2021 | NA | 17.89 | AY.59 |
| COVPSU-00473 | female | Narathiwat | 3/11/2021 | NA | 22.24 | AY.59 |
| COVPSU-00474 | female | Narathiwat | 4/11/2021 | NA | 15.02 | AY.59 |
| COVPSU-00475 | female | Narathiwat | 4/11/2021 | NA | 20.65 | AY.59 |
| COVPSU-00476 | male | Narathiwat | 5/11/2021 | NA | 14.19 | AY.85 |
| COVPSU-00477 | female | Narathiwat | 5/11/2021 | NA | 13.88 | AY.85 |
| COVPSU-00478 | male | Narathiwat | 3/11/2021 | NA | 30.37 | AY.85 |
| COVPSU-00479 | female | Narathiwat | 5/11/2021 | NA | 19.63 | AY.30 |
| COVPSU-00480 | female | Narathiwat | 8/11/2021 | NA | 16.87 | AY.85 |
| COVPSU-00481 | male | Narathiwat | 8/11/2021 | NA | 14.88 | AY.59 |
| COVPSU-00482 | male | Narathiwat | 8/11/2021 | NA | 19.01 | AY.85 |
| COVPSU-00483 | female | Narathiwat | 8/11/2021 | NA | 16.55 | AY.85 |
| COVPSU-00484 | female | Narathiwat | 8/11/2021 | NA | 14.37 | AY.85 |
| COVPSU-00485 | male | Narathiwat | 8/11/2021 | NA | 21.97 | AY.85 |
| COVPSU-00486 | male | Narathiwat | 8/11/2021 | NA | 16.84 | AY.85 |
| COVPSU-00487 | male | Narathiwat | 8/11/2021 | NA | 16.52 | AY.85 |
| COVPSU-00488 | female | Narathiwat | 8/11/2021 | NA | 23.47 | AY.59 |
| COVPSU-00489 | female | Narathiwat | 8/11/2021 | NA | 18.72 | AY.85 |
| COVPSU-00490 | female | Narathiwat | 8/11/2021 | NA | 17.95 | AY.85 |
| COVPSU-00491 | male | Narathiwat | 8/11/2021 | NA | 24.05 | AY.85 |
| COVPSU-00492 | male | Narathiwat | 8/11/2021 | NA | 21.13 | AY.85 |
| COVPSU-00493 | male | Narathiwat | 8/11/2021 | NA | 16.32 | AY.85 |
| COVPSU-00494 | male | Narathiwat | 8/11/2021 | NA | 21.73 | AY.85 |
| COVPSU-00495 | female | Narathiwat | 6/11/2021 | NA | NA | AY.85 |
| COVPSU-00496 | female | Narathiwat | 9/11/2021 | NA | 19.3 | AY.85 |
| COVPSU-00497 | male | Narathiwat | 9/11/2021 | NA | 22.92 | AY.85 |
| COVPSU-00498 | male | Narathiwat | 9/11/2021 | NA | 17.44 | AY.85 |
| COVPSU-00499 | female | Yala | 19/10/2021 | 47 | NA | AY.85 |
| COVPSU-00500 | female | Yala | 18/10/2021 | 37 | NA | AY.85 |
| COVPSU-00501 | female | Yala | 23/10/2021 | 27 | NA | AY.85 |
| COVPSU-00502 | female | Songkhla | 23/10/2021 | 44 | 12.78 | AY.30 |
| COVPSU-00503 | male | Songkhla | 23/10/2021 | 29 | 13.32 | AY.30 |
| COVPSU-00504 | male | Songkhla | 23/10/2021 | 34 | 14.76 | AY.30 |
| COVPSU-00505 | female | Narathiwat | 20/10/2021 | NA | 17.14 | B.1.1.7 |
| COVPSU-00508 | male | Narathiwat | 22/10/2021 | NA | 25.5 | AY.39.1 |
| COVPSU-00509 | male | Yala | 21/10/2021 | 29 | 17.22 | AY.85 |
| COVPSU-00510 | female | Yala | 24/10/2021 | 45 | 19.69 | AY.85 |
| COVPSU-00511 | female | Yala | 24/10/2021 | 23 | 17.32 | AY.85 |
| COVPSU-00513 | female | Yala | 24/10/2021 | 35 | 16.06 | AY.59 |
| COVPSU-00514 | female | Yala | 24/10/2021 | 44 | 16.07 | AY.85 |
| COVPSU-00515 | female | Yala | 25/10/2021 | 62 | 16.59 | AY.85 |
| COVPSU-00516 | female | Yala | 25/10/2021 | 37 | 14.48 | AY.85 |
| COVPSU-00517 | male | Yala | 14/10/2021 | 37 | 18 | AY.85 |
| COVPSU-00518 | female | Narathiwat | 27/10/2021 | 56 | 26.37 | AY.85 |
| COVPSU-00519 | male | Narathiwat | 25/10/2021 | 38 | 16.53 | AY.85 |
| COVPSU-00520 | female | Pattani | 27/10/2021 | NA | 18.51 | AY.85 |
| COVPSU-00521 | male | Pattani | 27/10/2021 | NA | 14.14 | AY.30 |
| COVPSU-00522 | female | Pattani | 26/10/2021 | NA | 10.94 | AY.85 |
| COVPSU-00523 | male | Pattani | 27/10/2021 | NA | 11.51 | AY.85 |
| COVPSU-00524 | female | Pattani | 27/10/2021 | NA | 12.54 | AY.30 |
| COVPSU-00525 | male | Songkhla | 26/10/2021 | 48 | 20.93 | AY.85 |
| COVPSU-00526 | male | Songkhla | 26/10/2021 | 34 | 19.66 | AY.30 |
| COVPSU-00527 | female | Songkhla | 26/10/2021 | 62 | 15.64 | AY.85 |
| COVPSU-00528 | female | Songkhla | 26/10/2021 | 43 | 13.89 | AY.85 |
| COVPSU-00529 | female | Songkhla | 26/10/2021 | 29 | 14.9 | AY.85 |
| COVPSU-00530 | female | Songkhla | 23/10/2021 | 45 | 13.14 | AY.85 |
| COVPSU-00531 | male | Songkhla | 21/10/2021 | 34 | 17.07 | AY.59 |
| COVPSU-00532 | female | Yala | 27/10/2021 | 89 | 24.06 | AY.85 |
| COVPSU-00533 | female | Yala | 24/10/2021 | 44 | 15.75 | AY.85 |
| COVPSU-00534 | female | Narathiwat | 27/10/2021 | 33 | 29.24 | AY.85 |
| COVPSU-00535 | female | Yala | 31/10/2021 | 40 | 16.17 | AY.85 |
| COVPSU-00536 | female | Yala | 31/10/2021 | 60 | 15.25 | AY.85 |
| COVPSU-00537 | female | Narathiwat | 29/10/2021 | NA | 16.9 | AY.85 |
| COVPSU-00538 | female | Narathiwat | 30/10/2021 | NA | 15.9 | AY.85 |
| COVPSU-00539 | female | Narathiwat | 29/10/2021 | NA | 17.24 | AY.85 |
| COVPSU-00540 | male | Narathiwat | 29/10/2021 | NA | 20.21 | AY.85 |
| COVPSU-00541 | female | Yala | 26/10/2021 | 92 | 15.09 | AY.85 |
| COVPSU-00542 | female | Yala | 28/10/2021 | 37 | 14.84 | AY.30 |
| COVPSU-00543 | male | Yala | 28/10/2021 | 80 | 27.78 | AY.85 |
| COVPSU-00544 | male | Yala | 29/10/2021 | 59 | 16.06 | AY.85 |
| COVPSU-00545 | female | Yala | 31/10/2021 | 25 | 21.91 | AY.85 |
| COVPSU-00547 | female | Narathiwat | 17/10/2021 | 101 | 20.98 | AY.85 |
| COVPSU-00548 | female | Narathiwat | 19/10/2021 | 100 | 20.85 | AY.85 |
| COVPSU-00549 | male | Narathiwat | 21/10/2021 | 50 | 23.63 | AY.86 |
| COVPSU-00550 | male | Narathiwat | 24/10/2021 | 25 | 23.55 | AY.79 |
| COVPSU-00551 | male | Narathiwat | 24/10/2021 | 70 | 15.06 | AY.85 |
| COVPSU-00552 | female | Narathiwat | 24/10/2021 | 34 | 24.48 | AY.59 |
| COVPSU-00553 | female | Narathiwat | 26/10/2021 | 85 | 25.85 | AY.85 |
| COVPSU-00555 | male | Narathiwat | 28/10/2021 | 53 | 22.84 | AY.85 |
| COVPSU-00556 | female | Narathiwat | 31/10/2021 | 71 | 15.21 | AY.30 |
| COVPSU-00557 | male | Narathiwat | 31/10/2021 | 27 | 16.21 | AY.85 |
| COVPSU-00558 | female | Songkhla | 2/11/2021 | NA | NA | AY.30 |
| COVPSU-00559 | female | Songkhla | 2/11/2021 | NA | NA | AY.85 |
| COVPSU-00560 | female | Songkhla | 2/11/2021 | NA | NA | AY.30 |
| COVPSU-00561 | male | Songkhla | 2/11/2021 | NA | 10.73 | AY.59 |
| COVPSU-00562 | female | Songkhla | 2/11/2021 | NA | 13.75 | AY.30 |
| COVPSU-00563 | female | Songkhla | 2/11/2021 | NA | 14.54 | AY.30 |
| COVPSU-00564 | female | Songkhla | 2/11/2021 | NA | 14.67 | AY.85 |
| COVPSU-00565 | male | Songkhla | 2/11/2021 | NA | 23.79 | B.1.617.2 |
| COVPSU-00566 | female | Songkhla | 29/10/2021 | NA | 15.78 | AY.30 |
| COVPSU-00567 | female | Songkhla | 29/10/2021 | NA | 12.68 | AY.30 |
| COVPSU-00571 | male | Songkhla | 2/11/2021 | NA | 18.67 | AY.30 |
| COVPSU-00572 | male | Songkhla | 2/11/2021 | NA | 18.22 | AY.85 |
| COVPSU-00573 | female | Songkhla | 2/11/2021 | NA | 16.05 | AY.85 |
| COVPSU-00574 | female | Songkhla | 2/11/2021 | NA | 14.68 | AY.85 |
| COVPSU-00575 | female | Songkhla | 28/10/2021 | NA | 9.13 | AY.85 |
| COVPSU-00576 | male | Songkhla | 27/10/2021 | NA | 18.64 | AY.30 |
| COVPSU-00577 | male | Songkhla | 27/10/2021 | NA | 19.32 | AY.30 |
| COVPSU-00578 | female | Songkhla | 3/11/2021 | 5 | 16.52 | AY.85 |
| COVPSU-00580 | male | Songkhla | 3/11/2021 | 21 | 16.74 | AY.85 |
| COVPSU-00581 | male | Narathiwat | 4/11/2021 | NA | 12.72 | AY.85 |
| COVPSU-00582 | male | Narathiwat | 4/11/2021 | NA | 15.59 | AY.85 |
| COVPSU-00583 | female | Narathiwat | 4/11/2021 | NA | 11.66 | AY.85 |
| COVPSU-00584 | female | Pattani | 3/11/2021 | NA | 12.88 | AY.85 |
| COVPSU-00585 | female | Pattani | 3/11/2021 | NA | 11.69 | AY.85 |
| COVPSU-00586 | female | Pattani | 3/11/2021 | NA | 19.26 | AY.85 |
| COVPSU-00587 | female | Pattani | 3/11/2021 | NA | 14.36 | AY.79 |
| COVPSU-00588 | female | Pattani | 1/11/2021 | 46 | 14.81 | AY.85 |
| COVPSU-00589 | male | Pattani | 31/10/2021 | 65 | 20.96 | AY.30 |
| COVPSU-00590 | female | Pattani | 3/11/2021 | 53 | 21.87 | AY.85 |
| COVPSU-00591 | female | Pattani | 1/11/2021 | 42 | 19.53 | AY.30 |
| COVPSU-00592 | female | Yala | 31/10/2021 | 78 | 16.86 | AY.85 |
| COVPSU-00593 | male | Yala | 17/10/2021 | 89 | 15.52 | AY.85 |
| COVPSU-00594 | female | Yala | 4/11/2021 | 44 | 18.27 | AY.85 |
| COVPSU-00595 | female | Narathiwat | 2/11/2021 | 77 | 19.09 | AY.85 |
| COVPSU-00596 | female | Narathiwat | 4/11/2021 | NA | 19.01 | AY.79 |
| COVPSU-00597 | female | Narathiwat | 4/11/2021 | NA | 19.57 | AY.85 |
| COVPSU-00598 | male | Narathiwat | 4/11/2021 | NA | 21.07 | AY.85 |
| COVPSU-00599 | female | Songkhla | 7/11/2021 | 29 | 16.26 | AY.30 |
| COVPSU-00600 | male | Songkhla | 7/11/2021 | 26 | 14.47 | AY.85 |
| COVPSU-00601 | female | Songkhla | 7/11/2021 | 15 | 19.6 | AY.85 |
| COVPSU-00602 | female | Narathiwat | 4/11/2021 | 45 | 23.25 | AY.85 |
| COVPSU-00603 | male | Narathiwat | 6/11/2021 | 31 | 14.96 | AY.85 |
| COVPSU-00604 | female | Narathiwat | 6/11/2021 | 19 | 19.4 | AY.85 |
| COVPSU-00605 | female | Narathiwat | 6/11/2021 | 53 | 15.85 | AY.85 |
| COVPSU-00607 | female | Yala | 5/11/2021 | 22 | 17.53 | AY.85 |
| COVPSU-00608 | female | Yala | 5/11/2021 | 42 | 18.93 | AY.85 |
| COVPSU-00609 | female | Yala | 5/11/2021 | 38 | 17.1 | AY.85 |
| COVPSU-00610 | female | Yala | 5/11/2021 | 33 | 15.22 | AY.85 |
| COVPSU-00611 | female | Narathiwat | 5/11/2021 | 36 | 26.87 | AY.85 |
| COVPSU-00613 | female | Narathiwat | 31/10/2021 | NA | 22.15 | AY.86 |
| COVPSU-00614 | male | Yala | 5/11/2021 | 30 | 14.83 | AY.85 |
| COVPSU-00615 | male | Yala | 5/11/2021 | 28 | 15.5 | AY.85 |
| COVPSU-00616 | female | Yala | 5/11/2021 | NA | 19.44 | AY.85 |
| COVPSU-00617 | female | Yala | 5/11/2021 | 29 | 18 | AY.85 |
| COVPSU-00618 | male | Pattani | 10/11/2021 | NA | 13.05 | AY.85 |
| COVPSU-00619 | male | Narathiwat | 9/11/2021 | 36 | 18.96 | AY.85 |
| COVPSU-00622 | female | Songkhla | 9/11/2021 | NA | 22.44 | AY.30 |
| COVPSU-00624 | female | Songkhla | 7/11/2021 | NA | 17.54 | AY.85 |
| COVPSU-00625 | male | Pattani | 2/11/2021 | NA | 21.33 | AY.85 |
| COVPSU-00626 | female | Pattani | 12/11/2021 | 34 | 18.08 | AY.85 |
| COVPSU-00627 | female | Pattani | 12/11/2021 | 23 | 20.73 | AY.85 |
| COVPSU-00628 | female | Pattani | 12/11/2021 | 36 | 18.05 | AY.85 |
| COVPSU-00629 | male | Pattani | 12/11/2021 | 60 | 20.65 | AY.85 |
| COVPSU-00630 | male | Pattani | 12/11/2021 | 55 | 24.44 | AY.85 |
| COVPSU-00631 | female | Songkhla | 7/11/2021 | 32 | 13.38 | AY.30 |
| COVPSU-00632 | female | Songkhla | 8/11/2021 | 25 | 14.67 | AY.85 |
| COVPSU-00633 | female | Narathiwat | 10/11/2021 | 29 | 18.34 | AY.85 |
| COVPSU-00634 | female | Narathiwat | 9/11/2021 | 58 | 16.2 | AY.85 |
| COVPSU-00635 | female | Yala | 14/11/2021 | 32 | 20.64 | AY.85 |
| COVPSU-00636 | female | Yala | 14/11/2021 | 26 | 16 | AY.85 |
| COVPSU-00637 | male | Yala | 14/11/2021 | NA | 19.84 | AY.85 |
| COVPSU-00638 | male | Songkhla | 15/11/2021 | NA | NA | AY.85 |
| COVPSU-00639 | female | Songkhla | 15/11/2021 | NA | NA | AY.85 |
| COVPSU-00640 | male | Songkhla | 15/11/2021 | NA | NA | AY.85 |
| COVPSU-00641 | male | Songkhla | 15/11/2021 | NA | NA | AY.85 |
| COVPSU-00642 | male | Songkhla | 15/11/2021 | NA | NA | AY.85 |
| COVPSU-00643 | male | Songkhla | 15/11/2021 | NA | NA | AY.85 |
| COVPSU-00644 | male | Songkhla | 15/11/2021 | NA | NA | AY.85 |
| COVPSU-00645 | male | Songkhla | 15/11/2021 | NA | NA | AY.85 |
| COVPSU-00646 | female | Narathiwat | 10/11/2021 | 59 | 14.81 | AY.85 |
| COVPSU-00647 | female | Narathiwat | 12/11/2021 | 72 | 18.31 | AY.85 |
| COVPSU-00648 | male | Narathiwat | 12/11/2021 | 56 | 15.32 | AY.85 |
| COVPSU-00649 | female | Narathiwat | 13/11/2021 | 83 | 14.52 | AY.85 |
| COVPSU-00650 | female | Narathiwat | 13/11/2021 | 37 | 18.85 | AY.85 |
| COVPSU-00651 | male | Narathiwat | 14/11/2021 | 10 | 26.08 | AY.85 |
| COVPSU-00652 | female | Narathiwat | 9/11/2021 | NA | 15.39 | AY.85 |
| COVPSU-00653 | female | Narathiwat | 12/11/2021 | 11 | 14.63 | AY.85 |
| COVPSU-00654 | male | Narathiwat | 10/11/2021 | 59 | 16.16 | AY.85 |
| COVPSU-00655 | male | Narathiwat | 10/11/2021 | 30 | 18.87 | AY.85 |
| COVPSU-00656 | female | Narathiwat | 7/11/2021 | 84 | 21.43 | AY.59 |
| COVPSU-00657 | male | Narathiwat | 7/11/2021 | 63 | 15 | AY.85 |
| COVPSU-00658 | female | Narathiwat | 11/11/2021 | 41 | 26.5 | AY.85 |
| COVPSU-00659 | male | Narathiwat | 11/11/2021 | 57 | 27.4 | AY.85 |
| COVPSU-00660 | female | Narathiwat | 11/11/2021 | 32 | 23.93 | AY.79 |
| COVPSU-00661 | male | Narathiwat | 13/11/2021 | 70 | 13.34 | AY.85 |
| COVPSU-00662 | male | Narathiwat | 12/11/2021 | 31 | 19.08 | AY.59 |
| COVPSU-00665 | male | Narathiwat | 12/11/2021 | 63 | 28.82 | AY.85 |
| COVPSU-00666 | male | Narathiwat | 15/11/2021 | 66 | 19.36 | AY.85 |
| COVPSU-00667 | male | Narathiwat | 10/11/2021 | 34 | 20.8 | AY.85 |
| COVPSU-00668 | female | Narathiwat | 10/11/2021 | 50 | 15.12 | AY.85 |
| COVPSU-00669 | female | Narathiwat | 10/11/2021 | 71 | 15.26 | AY.85 |
| COVPSU-00670 | male | Narathiwat | 10/11/2021 | 27 | 18.12 | AY.85 |
| COVPSU-00671 | female | Narathiwat | 10/11/2021 | 3 | 16.44 | AY.85 |
| COVPSU-00672 | female | Narathiwat | 10/11/2021 | 30 | 14.56 | AY.79 |
| COVPSU-00673 | female | Narathiwat | 11/11/2021 | 22 | 19.72 | AY.59 |
| COVPSU-00674 | female | Narathiwat | 11/11/2021 | 67 | 13.23 | AY.85 |
| COVPSU-00675 | male | Narathiwat | 11/11/2021 | 51 | 16.83 | AY.85 |
| COVPSU-00676 | female | Narathiwat | 11/11/2021 | 22 | 22.27 | AY.85 |
| COVPSU-00677 | female | Narathiwat | 12/11/2021 | 18 | 20.21 | AY.85 |
| COVPSU-00678 | male | Narathiwat | 12/11/2021 | 39 | 21.2 | AY.85 |
| COVPSU-00679 | male | Narathiwat | 12/11/2021 | 23 | 24.05 | AY.59 |
| COVPSU-00680 | female | Narathiwat | 12/11/2021 | 26 | 22.17 | AY.85 |
| COVPSU-00681 | male | Narathiwat | 12/11/2021 | 54 | 19.81 | AY.85 |
| COVPSU-00682 | female | Narathiwat | 12/11/2021 | 46 | 18.92 | AY.85 |
| COVPSU-00683 | female | Narathiwat | 12/11/2021 | 31 | 15.95 | AY.85 |
| COVPSU-00684 | female | Narathiwat | 12/11/2021 | 60 | 24.64 | AY.85 |
| COVPSU-00685 | male | Narathiwat | 12/11/2021 | 80 | 20.75 | AY.85 |
| COVPSU-00686 | female | Narathiwat | 14/11/2021 | 25 | 14.83 | AY.85 |
| COVPSU-00687 | female | Songkhla | 18/11/2021 | NA | 21.13 | AY.30 |
| COVPSU-00688 | male | Songkhla | 18/11/2021 | NA | 20.14 | AY.30 |
| COVPSU-00689 | female | Songkhla | 18/11/2021 | NA | 18.46 | AY.85 |
| COVPSU-00690 | female | Songkhla | 18/11/2021 | NA | 13.16 | AY.30 |
| COVPSU-00691 | female | Songkhla | 18/11/2021 | NA | 17.1 | B.1.617.2 |
| COVPSU-00692 | female | Songkhla | 18/11/2021 | NA | 15.08 | AY.85 |
| COVPSU-00693 | female | Yala | 17/11/2021 | NA | 16.02 | AY.85 |
| COVPSU-00694 | female | Yala | 17/11/2021 | NA | 15.34 | AY.85 |
| COVPSU-00695 | female | Yala | 17/11/2021 | NA | 14.18 | AY.85 |
| COVPSU-00696 | female | Yala | 17/11/2021 | NA | 14.37 | AY.85 |
| COVPSU-00697 | male | Yala | 17/11/2021 | NA | 14.09 | AY.59 |
| COVPSU-00698 | male | Yala | 17/11/2021 | NA | 14.13 | AY.85 |
| COVPSU-00699 | male | Pattani | 17/11/2021 | 43 | 21.21 | AY.85 |
| COVPSU-00700 | male | Pattani | 17/11/2021 | 35 | 16.34 | AY.85 |
| COVPSU-00701 | male | Pattani | 17/11/2021 | 21 | 13.89 | AY.85 |
| COVPSU-00702 | male | Pattani | 17/11/2021 | 21 | 19.85 | AY.85 |
| COVPSU-00703 | male | Pattani | 17/11/2021 | 57 | 25.36 | AY.59 |
| COVPSU-00704 | male | Pattani | 17/11/2021 | 44 | 14.38 | AY.85 |
| COVPSU-00705 | male | Pattani | 17/11/2021 | 28 | 14.91 | AY.85 |
| COVPSU-00706 | female | Pattani | 17/11/2021 | 7 | 17.94 | AY.85 |
| COVPSU-00707 | female | Yala | 7/11/2021 | 24 | 16 | AY.85 |
| COVPSU-00708 | male | Yala | 15/11/2021 | 65 | 23.69 | AY.85 |
| COVPSU-00709 | male | Yala | 15/11/2021 | 62 | 23.42 | AY.85 |
| COVPSU-00711 | male | Yala | 15/11/2021 | 53 | 20.37 | AY.85 |
| COVPSU-00712 | male | Yala | 15/11/2021 | 51 | 19.35 | AY.85 |
| COVPSU-00713 | male | Yala | 15/11/2021 | 25 | 18.96 | AY.85 |
| COVPSU-00714 | female | Yala | 15/11/2021 | 26 | 14.46 | AY.85 |
| COVPSU-00715 | male | Yala | 15/11/2021 | 44 | 18.4 | AY.85 |
| COVPSU-00716 | male | Yala | 15/11/2021 | 56 | 26.19 | AY.85 |
| COVPSU-00717 | male | Yala | 15/11/2021 | 58 | 18.04 | AY.85 |
| COVPSU-00718 | male | Yala | 15/11/2021 | 27 | 21.32 | AY.85 |
| COVPSU-00719 | male | Yala | 15/11/2021 | 57 | 29.6 | AY.85 |
| COVPSU-00720 | female | Yala | 15/11/2021 | 38 | 27.62 | AY.85 |
| COVPSU-00721 | male | Yala | 15/11/2021 | 57 | 26.86 | AY.85 |
| COVPSU-00722 | male | Yala | 15/11/2021 | 47 | 18.74 | AY.85 |
| COVPSU-00723 | male | Yala | 15/11/2021 | 26 | 24.63 | AY.85 |
| COVPSU-00724 | male | Yala | 15/11/2021 | 35 | 18.23 | AY.85 |
| COVPSU-00725 | male | Yala | 15/11/2021 | 41 | 28.96 | AY.85 |
| COVPSU-00726 | male | Yala | 15/11/2021 | 51 | 25.88 | AY.85 |
| COVPSU-00727 | male | Yala | 15/11/2021 | 40 | 17.74 | AY.85 |
| COVPSU-00728 | male | Yala | 15/11/2021 | 34 | 24.84 | AY.85 |
| COVPSU-00729 | female | Yala | 15/11/2021 | 21 | 15.22 | AY.85 |
| COVPSU-00730 | female | Yala | 15/11/2021 | 34 | 15.2 | AY.85 |
| COVPSU-00731 | female | Yala | 15/11/2021 | 31 | 16.13 | AY.85 |
| COVPSU-00732 | male | Songkhla | 8/11/2021 | NA | NA | AY.85 |
| COVPSU-00733 | male | Songkhla | 5/11/2021 | NA | NA | AY.85 |
| COVPSU-00734 | male | Songkhla | 5/11/2021 | NA | NA | AY.85 |
| COVPSU-00735 | female | Songkhla | 15/11/2021 | NA | NA | AY.85 |
| COVPSU-00736 | female | Songkhla | 15/11/2021 | NA | NA | AY.85 |
| COVPSU-00737 | female | Songkhla | 13/11/2021 | NA | NA | AY.85 |
| COVPSU-00738 | female | Songkhla | 12/11/2021 | NA | NA | AY.85 |
| COVPSU-00739 | female | Pattani | 15/11/2021 | 66 | 17.2 | AY.124 |
| COVPSU-00740 | male | Pattani | 15/11/2021 | 54 | 22.08 | AY.85 |
| COVPSU-00741 | female | Pattani | 15/11/2021 | 65 | 16.43 | AY.85 |
| COVPSU-00742 | male | Pattani | 15/11/2021 | 55 | 27.84 | AY.85 |
| COVPSU-00743 | male | Pattani | 15/11/2021 | 67 | 19.48 | AY.85 |
| COVPSU-00744 | female | Yala | 7/11/2021 | 86 | 18.7 | AY.85 |
| COVPSU-00745 | male | Yala | 8/11/2021 | 61 | 24.48 | AY.85 |
| COVPSU-00746 | female | Pattani | 15/11/2021 | 34 | 18.68 | AY.85 |
| COVPSU-00747 | female | Pattani | 15/11/2021 | 37 | 18.1 | AY.85 |
| COVPSU-00748 | female | Pattani | 15/11/2021 | 36 | 18.86 | AY.85 |
| COVPSU-00749 | male | Pattani | 15/11/2021 | 44 | 14.71 | AY.30 |
| COVPSU-00750 | female | Pattani | 15/11/2021 | 24 | 16.66 | AY.85 |
| COVPSU-00751 | female | Pattani | 15/11/2021 | 66 | 17.97 | AY.85 |
| COVPSU-00752 | female | Pattani | 15/11/2021 | 30 | 16.73 | AY.85 |
| COVPSU-00753 | male | Pattani | 15/11/2021 | 33 | 18.64 | AY.85 |
| COVPSU-00754 | male | Pattani | 15/11/2021 | 53 | 17.63 | AY.85 |
| COVPSU-00757 | female | Yala | 15/11/2021 | 41 | 16.7 | AY.85 |
| COVPSU-00758 | male | Yala | 21/11/2021 | 13 | 16.56 | AY.85 |
| COVPSU-00759 | female | Yala | 21/11/2021 | 71 | 14.42 | AY.85 |
| COVPSU-00760 | female | Yala | 21/11/2021 | 57 | 23.19 | AY.85 |
| COVPSU-00761 | male | Yala | 17/11/2021 | 57 | 13.15 | AY.85 |
| COVPSU-00762 | female | Yala | 17/11/2021 | 35 | 14.43 | AY.85 |
| COVPSU-00763 | female | Yala | 18/11/2021 | 61 | 11.88 | AY.85 |
| COVPSU-00764 | female | Yala | 18/11/2021 | 58 | 12.22 | AY.85 |
| COVPSU-00765 | male | Narathiwat | 16/11/2021 | 13 | 21.75 | AY.85 |
| COVPSU-00766 | female | Narathiwat | 16/11/2021 | 55 | 18.3 | AY.85 |
| COVPSU-00767 | female | Narathiwat | 16/11/2021 | 58 | 15.53 | AY.85 |
| COVPSU-00768 | male | Narathiwat | 16/11/2021 | 55 | 16.28 | AY.85 |
| COVPSU-00769 | male | Narathiwat | 16/11/2021 | 41 | 21.09 | AY.85 |
| COVPSU-00770 | female | Narathiwat | 17/11/2021 | 58 | 20.74 | AY.108 |
| COVPSU-00771 | female | Narathiwat | 17/11/2021 | 47 | 14.16 | AY.59 |
| COVPSU-00772 | female | Narathiwat | 18/11/2021 | 54 | 15.62 | AY.85 |
| COVPSU-00773 | male | Narathiwat | 19/11/2021 | 68 | 15.65 | AY.30 |
| COVPSU-00774 | female | Narathiwat | 19/11/2021 | 35 | 18.07 | AY.85 |
| COVPSU-00775 | female | Narathiwat | 20/11/2021 | 82 | 21.63 | AY.85 |
| COVPSU-00776 | female | Narathiwat | 20/11/2021 | 51 | 17.45 | AY.85 |
| COVPSU-00777 | female | Narathiwat | 21/11/2021 | NA | 18.35 | AY.85 |
| COVPSU-00778 | female | Narathiwat | 21/11/2021 | 19 | 13.21 | AY.85 |
| COVPSU-00779 | female | Narathiwat | 16/11/2021 | 42 | 15.13 | AY.108 |
| COVPSU-00780 | male | Narathiwat | 16/11/2021 | 62 | 11.12 | AY.85 |
| COVPSU-00781 | male | Narathiwat | 16/11/2021 | NA | 12.05 | AY.85 |
| COVPSU-00782 | male | Narathiwat | 19/11/2021 | 52 | 12.44 | AY.85 |
| COVPSU-00783 | female | Narathiwat | 15/11/2021 | 29 | 28.23 | AY.85 |
| COVPSU-00785 | female | Narathiwat | 17/11/2021 | 38 | 22.7 | AY.85 |
| COVPSU-00786 | female | Narathiwat | 18/11/2021 | 41 | 17.65 | AY.85 |
| COVPSU-00787 | female | Narathiwat | 18/11/2021 | 5 | 26.01 | AY.85 |
| COVPSU-00788 | female | Narathiwat | 18/11/2021 | 100 | 11.84 | AY.85 |
| COVPSU-00789 | female | Narathiwat | 19/11/2021 | 36 | 24.59 | AY.85 |
| COVPSU-00790 | male | Narathiwat | 15/11/2021 | 38 | 15.65 | AY.45 |
| COVPSU-00791 | female | Narathiwat | 15/11/2021 | 59 | 15.85 | AY.85 |
| COVPSU-00792 | female | Narathiwat | 15/11/2021 | 30 | 17.3 | AY.85 |
| COVPSU-00793 | female | Narathiwat | 15/11/2021 | 17 | 20.68 | AY.59 |
| COVPSU-00794 | male | Narathiwat | 15/11/2021 | 3 | 18.34 | AY.85 |
| COVPSU-00795 | female | Narathiwat | 15/11/2021 | 11 | 17.72 | AY.85 |
| COVPSU-00796 | female | Narathiwat | 16/11/2021 | 44 | 16.58 | AY.85 |
| COVPSU-00797 | female | Narathiwat | 17/11/2021 | 33 | 20.97 | AY.85 |
| COVPSU-00798 | female | Narathiwat | 17/11/2021 | 34 | 18.47 | AY.85 |
| COVPSU-00799 | male | Narathiwat | 17/11/2021 | 20 | 15.75 | AY.59 |
| COVPSU-00800 | female | Narathiwat | 17/11/2021 | 63 | 19.52 | AY.85 |
| COVPSU-00801 | female | Narathiwat | 17/11/2021 | 41 | 17.47 | AY.85 |
| COVPSU-00802 | female | Narathiwat | 17/11/2021 | 32 | 20.23 | AY.104 |
| COVPSU-00803 | female | Narathiwat | 20/11/2021 | 71 | 10.64 | AY.108 |
| COVPSU-00805 | female | Songkhla | 19/11/2021 | 20 | 29.18 | B.1.617.2 |
| COVPSU-00806 | female | Songkhla | 19/11/2021 | 23 | 25.69 | AY.30 |
| COVPSU-00807 | female | Songkhla | 17/11/2021 | 33 | 29.34 | AY.85 |
| COVPSU-00808 | female | Songkhla | 17/11/2021 | 27 | 12.46 | AY.85 |
| COVPSU-00809 | female | Songkhla | 18/11/2021 | 34 | 20.27 | AY.85 |
| COVPSU-00810 | female | Songkhla | 12/11/2021 | 61 | 13.41 | AY.85 |
| COVPSU-00811 | female | Songkhla | 21/11/2021 | 82 | 34.5 | AY.85 |
| COVPSU-00812 | male | Songkhla | 17/11/2021 | 34 | 15.11 | AY.30 |
| COVPSU-00813 | female | Songkhla | 21/11/2021 | 22 | 11.9 | AY.30 |
| COVPSU-00814 | female | Songkhla | 21/11/2021 | 38 | 19.05 | AY.30 |
| COVPSU-00815 | male | Songkhla | 22/11/2021 | 21 | 15.73 | AY.85 |
| COVPSU-00816 | male | Songkhla | 22/11/2021 | 23 | 14.23 | AY.85 |
| COVPSU-00817 | male | Songkhla | 22/11/2021 | 29 | 17.1 | AY.30 |
| COVPSU-00821 | male | Pattani | 18/11/2021 | 44 | 17.62 | AY.85 |
| COVPSU-00822 | female | Pattani | 18/11/2021 | 67 | 19.2 | AY.85 |
| COVPSU-00823 | female | Pattani | 18/11/2021 | 34 | 24.14 | AY.85 |
| COVPSU-00824 | female | Pattani | 19/11/2021 | 36 | 19.35 | AY.85 |
| COVPSU-00825 | male | Pattani | 19/11/2021 | 33 | 20.19 | AY.85 |
| COVPSU-00826 | female | Pattani | 21/11/2021 | 66 | 18.7 | AY.85 |
| COVPSU-00827 | female | Pattani | 21/11/2021 | 35 | 25.69 | AY.85 |
| COVPSU-00828 | female | Pattani | 22/11/2021 | 25 | 14.9 | AY.30 |
| COVPSU-00829 | male | Songkhla | 27/11/2021 | NA | 17.06 | AY.85 |
| COVPSU-00830 | male | Songkhla | 27/11/2021 | NA | 16.63 | AY.30 |
| COVPSU-00831 | female | Songkhla | 27/11/2021 | NA | 16.33 | AY.59 |
| COVPSU-00832 | male | Songkhla | 27/11/2021 | NA | 16.46 | AY.85 |
| COVPSU-00833 | male | Songkhla | 27/11/2021 | NA | 21.01 | AY.30 |
| COVPSU-00834 | male | Songkhla | 27/11/2021 | NA | 16.35 | AY.85 |
| COVPSU-00835 | male | Songkhla | 27/11/2021 | NA | 16.7 | AY.85 |
| COVPSU-00836 | male | Songkhla | 27/11/2021 | NA | 19.05 | AY.85 |
| COVPSU-00837 | female | Songkhla | 21/11/2021 | 12 | 22.66 | B.1.1.7 |
| COVPSU-00838 | male | Songkhla | 18/11/2021 | 47 | 18.8 | AY.91 |
| COVPSU-00839 | female | Songkhla | 28/11/2021 | NA | 14.29 | AY.85 |
| COVPSU-00840 | male | Songkhla | 28/11/2021 | NA | 23.16 | AY.85 |
| COVPSU-00841 | male | Songkhla | 27/11/2021 | NA | 18.64 | AY.85 |
| COVPSU-00842 | female | Songkhla | 27/11/2021 | NA | 22.01 | AY.85 |
| COVPSU-00843 | male | Songkhla | 26/11/2021 | NA | 17.41 | AY.108 |
| COVPSU-00844 | female | Songkhla | 26/11/2021 | NA | 18.06 | AY.108 |
| COVPSU-00845 | female | Songkhla | 25/11/2021 | NA | 17.45 | AY.85 |
| COVPSU-00846 | male | Songkhla | 25/11/2021 | NA | 14.58 | AY.85 |
| COVPSU-00847 | male | Songkhla | 27/11/2021 | NA | 17.67 | AY.85 |
| COVPSU-00848 | female | Songkhla | 26/11/2021 | NA | 14.92 | AY.85 |
| COVPSU-00849 | female | Songkhla | 26/11/2021 | NA | 21.16 | AY.85 |
| COVPSU-00850 | female | Songkhla | 26/11/2021 | NA | 13.37 | AY.59 |
| COVPSU-00851 | female | Yala | 26/11/2021 | 34 | 14.78 | AY.85 |
| COVPSU-00852 | male | Yala | 25/11/2021 | 21 | 12.37 | AY.85 |
| COVPSU-00853 | female | Yala | 25/11/2021 | 33 | 18.05 | AY.85 |
| COVPSU-00854 | female | Yala | 25/11/2021 | 29 | 13.25 | AY.85 |
| COVPSU-00855 | female | Yala | 27/11/2021 | 43 | 13.13 | AY.85 |
| COVPSU-00856 | female | Narathiwat | 22/11/2021 | 57 | 18.15 | AY.85 |
| COVPSU-00857 | male | Narathiwat | 22/11/2021 | 2 | 17.78 | AY.85 |
| COVPSU-00858 | female | Narathiwat | 22/11/2021 | 37 | 22.73 | AY.85 |
| COVPSU-00859 | female | Narathiwat | 22/11/2021 | 32 | 23.98 | AY.85 |
| COVPSU-00860 | female | Narathiwat | 22/11/2021 | 34 | 19.78 | AY.85 |
| COVPSU-00861 | female | Narathiwat | 22/11/2021 | 68 | 20.05 | AY.59 |
| COVPSU-00862 | female | Narathiwat | 22/11/2021 | 55 | 15.36 | AY.108 |
| COVPSU-00863 | female | Narathiwat | 22/11/2021 | 55 | 15.67 | AY.85 |
| COVPSU-00864 | female | Narathiwat | 23/11/2021 | 22 | 20 | AY.85 |
| COVPSU-00865 | female | Narathiwat | 23/11/2021 | 22 | 25.49 | AY.85 |
| COVPSU-00866 | male | Narathiwat | 23/11/2021 | 71 | 19.01 | AY.85 |
| COVPSU-00867 | female | Narathiwat | 24/11/2021 | 35 | 15.73 | AY.85 |
| COVPSU-00868 | male | Narathiwat | 25/11/2021 | NA | 17.14 | AY.85 |
| COVPSU-00869 | male | Narathiwat | 25/11/2021 | 43 | 21.38 | AY.85 |
| COVPSU-00870 | male | Narathiwat | 26/11/2021 | 23 | 15.07 | AY.85 |
| COVPSU-00871 | male | Narathiwat | 26/11/2021 | 2 | 16.92 | AY.85 |
| COVPSU-00872 | male | Narathiwat | 26/11/2021 | 27 | 18.17 | B.1.617.2 |
| COVPSU-00873 | female | Narathiwat | 26/11/2021 | 28 | 18.1 | AY.85 |
| COVPSU-00874 | female | Narathiwat | 26/11/2021 | 26 | N/A | AY.85 |
| COVPSU-00875 | female | Narathiwat | 22/11/2021 | 65 | 17.9 | AY.85 |
| COVPSU-00876 | male | Narathiwat | 22/11/2021 | 1 | 19.73 | AY.85 |
| COVPSU-00877 | male | Narathiwat | 22/11/2021 | 67 | 15.61 | AY.85 |
| COVPSU-00878 | male | Narathiwat | 22/11/2021 | 75 | 16.92 | AY.85 |
| COVPSU-00879 | male | Narathiwat | 22/11/2021 | 3 | 11.25 | AY.85 |
| COVPSU-00880 | male | Narathiwat | 23/11/2021 | NA | 14.77 | AY.85 |
| COVPSU-00881 | female | Narathiwat | 24/11/2021 | NA | 17.62 | AY.85 |
| COVPSU-00882 | female | Narathiwat | 24/11/2021 | NA | 14.67 | AY.59 |
| COVPSU-00883 | female | Narathiwat | 24/11/2021 | NA | 17.28 | AY.59 |
| COVPSU-00884 | female | Narathiwat | 26/11/2021 | 85 | 16.22 | AY.85 |
| COVPSU-00885 | male | Narathiwat | 27/11/2021 | NA | 22.98 | AY.85 |
| COVPSU-00886 | female | Narathiwat | 27/11/2021 | NA | 12.84 | AY.85 |
| COVPSU-00887 | female | Narathiwat | 27/11/2021 | 34 | 13.16 | AY.85 |
| COVPSU-00888 | male | Narathiwat | 22/11/2021 | 47 | 20.85 | AY.85 |
| COVPSU-00889 | female | Narathiwat | 22/11/2021 | 63 | 12.62 | AY.85 |
| COVPSU-00890 | female | Narathiwat | 23/11/2021 | 40 | 14.85 | AY.85 |
| COVPSU-00891 | male | Narathiwat | 23/11/2021 | 46 | 14.32 | AY.85 |
| COVPSU-00892 | female | Narathiwat | 23/11/2021 | 54 | 17.54 | AY.85 |
| COVPSU-00893 | male | Narathiwat | 24/11/2021 | 55 | 12.42 | AY.61 |
| COVPSU-00894 | female | Narathiwat | 27/11/2021 | 76 | 29.32 | AY.85 |
| COVPSU-00895 | female | Songkhla | 26/11/2021 | 54 | 15.35 | AY.85 |
| COVPSU-00896 | male | Songkhla | 26/11/2021 | 52 | 15.02 | AY.30 |
| COVPSU-00897 | male | Songkhla | 27/11/2021 | 5 | 13.78 | AY.85 |
| COVPSU-00898 | female | Songkhla | 29/11/2021 | 41 | 19.93 | AY.85 |
| COVPSU-00899 | female | Songkhla | 29/11/2021 | 41 | 14.13 | AY.85 |
| COVPSU-00900 | female | Songkhla | 30/11/2021 | 26 | 11.62 | AY.85 |
| COVPSU-00901 | male | Songkhla | 30/11/2021 | 24 | 20.21 | AY.85 |
| COVPSU-00902 | female | Yala | 1/12/2021 | NA | 13.57 | AY.85 |
| COVPSU-00903 | female | Yala | 1/12/2021 | NA | 14.39 | AY.85 |
| COVPSU-00904 | female | Yala | 1/12/2021 | NA | 16.98 | AY.85 |
| COVPSU-00905 | male | Yala | 1/12/2021 | NA | 19.03 | AY.85 |
| COVPSU-00906 | female | Yala | 1/12/2021 | NA | 16.09 | AY.85 |
| COVPSU-00907 | male | Yala | 1/12/2021 | NA | 18.01 | AY.85 |
| COVPSU-00908 | female | Yala | 1/12/2021 | NA | 13.82 | AY.85 |
| COVPSU-00909 | female | Yala | 1/12/2021 | NA | 15.04 | AY.85 |
| COVPSU-00910 | female | Yala | 1/12/2021 | NA | 13.98 | AY.85 |
| COVPSU-00911 | female | Yala | 1/12/2021 | NA | 23.4 | AY.85 |
| COVPSU-00912 | male | Yala | 1/12/2021 | NA | 21.19 | AY.85 |
| COVPSU-00913 | female | Songkhla | 1/12/2021 | 37 | 16.16 | AY.85 |
| COVPSU-00914 | male | Songkhla | 30/11/2021 | 41 | 18.22 | AY.85 |
| COVPSU-00915 | male | Songkhla | 30/11/2021 | 21 | 21.13 | AY.85 |
| COVPSU-00916 | male | Songkhla | 25/11/2021 | NA | 14.19 | AY.85 |
| COVPSU-00917 | male | Songkhla | 23/11/2021 | NA | 21.9 | AY.30 |
| COVPSU-00918 | male | Songkhla | 27/11/2021 | NA | 15.33 | AY.85 |
| COVPSU-00919 | female | Songkhla | 1/12/2021 | NA | 14.04 | AY.30 |
| COVPSU-00920 | male | Songkhla | 29/11/2021 | NA | 18.01 | AY.30 |
| COVPSU-00921 | male | Songkhla | 30/11/2021 | NA | 21.24 | AY.85 |
| COVPSU-00922 | female | Songkhla | 30/11/2021 | NA | 21.43 | AY.85 |
| COVPSU-00923 | female | Songkhla | 30/11/2021 | NA | 25.22 | AY.85 |
| COVPSU-00924 | male | Songkhla | 30/11/2021 | NA | 13.63 | AY.85 |
| COVPSU-00925 | female | Songkhla | 30/11/2021 | NA | 22.46 | AY.85 |
| COVPSU-00926 | female | Songkhla | 30/11/2021 | NA | 12.27 | AY.85 |
| COVPSU-00927 | female | Songkhla | 30/11/2021 | NA | 25.21 | AY.85 |
| COVPSU-00928 | female | Songkhla | 30/11/2021 | NA | 14.78 | AY.85 |
| COVPSU-00929 | female | Songkhla | 30/11/2021 | NA | 10.18 | AY.85 |
| COVPSU-00930 | female | Songkhla | 30/11/2021 | NA | 13.37 | AY.85 |
| COVPSU-00931 | female | Narathiwat | 22/11/2021 | 71 | 19.81 | AY.59 |
| COVPSU-00932 | male | Narathiwat | 22/11/2021 | 9 | 17.55 | AY.85 |
| COVPSU-00933 | female | Narathiwat | 22/11/2021 | 22 | 17.41 | AY.85 |
| COVPSU-00934 | female | Narathiwat | 23/11/2021 | 58 | 20.69 | AY.85 |
| COVPSU-00935 | female | Narathiwat | 24/11/2021 | 25 | 16.42 | AY.85 |
| COVPSU-00936 | female | Narathiwat | 25/11/2021 | 58 | 24.43 | AY.85 |
| COVPSU-00937 | male | Narathiwat | 25/11/2021 | 23 | 21.49 | AY.85 |
| COVPSU-00938 | female | Narathiwat | 25/11/2021 | 27 | 18.97 | AY.85 |
| COVPSU-00939 | male | Narathiwat | 27/11/2021 | 36 | 15.14 | AY.85 |
| COVPSU-00940 | female | Narathiwat | 28/11/2021 | 61 | 15.12 | AY.30 |
| COVPSU-00944 | female | Yala | 29/11/2021 | 35 | 14.96 | AY.85 |
| COVPSU-00945 | female | Yala | 19/11/2021 | 87 | 19.21 | AY.85 |
| COVPSU-00946 | male | Songkhla | 2/12/2021 | 60 | 13.42 | AY.85 |
| COVPSU-00947 | female | Songkhla | 2/12/2021 | 29 | 13.48 | AY.85 |
| COVPSU-00948 | male | Songkhla | 2/12/2021 | 9 | 14.59 | AY.85 |
| COVPSU-00949 | male | Songkhla | 2/12/2021 | 27 | 12.88 | AY.85 |
| COVPSU-00950 | female | Songkhla | 2/12/2021 | 59 | 15.43 | AY.85 |
| COVPSU-00951 | male | Songkhla | 2/12/2021 | 3 | 13.48 | B.1.617.2 |
| COVPSU-00952 | male | Narathiwat | 8/11/2021 | NA | 17.66 | AY.85 |
| COVPSU-00953 | female | Narathiwat | 8/11/2021 | NA | 23.12 | AY.85 |
| COVPSU-00954 | male | Narathiwat | 10/11/2021 | NA | 20.85 | AY.59 |
| COVPSU-00955 | female | Narathiwat | 12/11/2021 | NA | 23.6 | AY.85 |
| COVPSU-00956 | male | Narathiwat | 12/11/2021 | NA | 17.67 | AY.85 |
| COVPSU-00957 | male | Songkhla | 3/12/2021 | 27 | 15.43 | AY.30 |
| COVPSU-00958 | male | Songkhla | 3/12/2021 | 35 | 11.3 | AY.43 |
| COVPSU-00959 | female | Songkhla | 3/12/2021 | 5 | 11.78 | AY.85 |
| COVPSU-00960 | male | Pattani | 25/11/2021 | 55 | 18.74 | AY.85 |
| COVPSU-00961 | male | Pattani | 26/11/2021 | 37 | 20.18 | AY.85 |
| COVPSU-00962 | male | Pattani | 28/11/2021 | 43 | 20.49 | AY.85 |
| COVPSU-00963 | male | Pattani | 29/11/2021 | 17 | 20.09 | AY.45 |
| COVPSU-00964 | female | Pattani | 30/11/2021 | 38 | 17.29 | AY.85 |
| COVPSU-00965 | male | Pattani | 30/11/2021 | 4 | 16.62 | AY.85 |
| COVPSU-00966 | male | Pattani | 1/12/2021 | 64 | 17.7 | AY.85 |
| COVPSU-00967 | female | Songkhla | 3/12/2021 | 1 | 18.19 | AY.85 |
| COVPSU-00968 | male | Songkhla | 3/12/2021 | NA | 25.36 | AY.59 |
| COVPSU-00969 | female | Songkhla | 3/12/2021 | 40 | 16.27 | AY.85 |
| COVPSU-00970 | female | Songkhla | 3/12/2021 | NA | 12.71 | AY.85 |
| COVPSU-00971 | female | Songkhla | 3/12/2021 | NA | 12.11 | AY.85 |
| COVPSU-00972 | male | Songkhla | 3/12/2021 | 19 | 23.49 | AY.85 |
| COVPSU-00973 | male | Songkhla | 3/12/2021 | 39 | 16.25 | AY.85 |
| COVPSU-00974 | female | Yala | 4/12/2021 | 62 | 12.21 | AY.85 |
| COVPSU-00975 | male | Yala | 5/12/2021 | 34 | 17.41 | AY.85 |
| COVPSU-00976 | male | Yala | 5/12/2021 | 69 | 15.24 | AY.85 |
| COVPSU-00983 | male | Songkhla | 3/12/2021 | 28 | 16.15 | AY.85 |
| COVPSU-00984 | male | Songkhla | 5/12/2021 | 37 | 21.15 | AY.85 |
| COVPSU-00985 | female | Songkhla | 5/12/2021 | 26 | 21.3 | AY.85 |
| COVPSU-00986 | male | Songkhla | 2/12/2021 | 27 | 17.4 | AY.85 |
| COVPSU-00987 | male | Songkhla | 1/12/2021 | 28 | 14.6 | AY.85 |
| COVPSU-00988 | male | Songkhla | 4/12/2021 | 34 | 20.38 | AY.85 |
| COVPSU-00989 | male | Songkhla | 4/12/2021 | 46 | 18 | AY.85 |
| COVPSU-00990 | male | Songkhla | 4/12/2021 | 60 | 13.88 | AY.30 |
| COVPSU-00991 | male | Songkhla | 4/12/2021 | 36 | 13.14 | AY.30 |
| COVPSU-00992 | male | Songkhla | 5/12/2021 | 35 | 17.45 | AY.85 |
| COVPSU-00993 | female | Songkhla | 3/12/2021 | 75 | 16.35 | AY.30 |
| COVPSU-00994 | female | Songkhla | 2/12/2021 | 76 | 15.93 | AY.30 |
| COVPSU-00995 | female | Songkhla | 1/12/2021 | 28 | 12.31 | AY.85 |
| COVPSU-00996 | female | Songkhla | 26/11/2021 | 72 | 18.04 | AY.85 |
| COVPSU-00997 | male | Narathiwat | 25/11/2021 | 51 | 16.05 | AY.59 |
| COVPSU-00998 | female | Narathiwat | 29/11/2021 | 74 | 14.21 | AY.85 |
| COVPSU-00999 | female | Narathiwat | 29/11/2021 | 31 | 13.7 | AY.61 |
| COVPSU-01000 | male | Narathiwat | 21/11/2021 | 72 | 13.21 | B.1.617.2 |
| COVPSU-01001 | female | Narathiwat | 30/11/2021 | 36 | 25 | B.1.617.2 |
| COVPSU-01002 | female | Narathiwat | 2/12/2021 | 30 | 16.55 | AY.85 |
| COVPSU-01003 | female | Narathiwat | 3/12/2021 | 46 | 19.56 | AY.59 |
| COVPSU-01004 | female | Narathiwat | 30/11/2021 | 32 | 11.14 | AY.85 |
| COVPSU-01005 | male | Narathiwat | 30/11/2021 | 28 | 14.06 | AY.85 |
| COVPSU-01006 | female | Narathiwat | 3/12/2021 | 33 | 12.52 | AY.85 |
| COVPSU-01007 | male | Narathiwat | 2/12/2021 | 30 | 13.29 | AY.85 |
| COVPSU-01008 | female | Narathiwat | 18/11/2021 | 76 | 24.42 | AY.85 |
| COVPSU-01009 | female | Narathiwat | 1/12/2021 | 35 | 18.79 | AY.85 |
| COVPSU-01010 | male | Narathiwat | 2/12/2021 | 56 | 18.42 | AY.85 |
| COVPSU-01011 | female | Narathiwat | 2/12/2021 | 42 | 25.44 | AY.85 |
| COVPSU-01012 | female | Yala | 2/12/2021 | 56 | 16.92 | AY.85 |
| COVPSU-01013 | male | Yala | 2/12/2021 | 4 | 14.32 | AY.85 |
| COVPSU-01014 | female | Yala | 3/12/2021 | 27 | 13.78 | AY.85 |
| COVPSU-01015 | female | Narathiwat | 29/11/2021 | 69 | 14.92 | AY.85 |
| COVPSU-01016 | female | Narathiwat | 29/11/2021 | 13 | 15.83 | AY.85 |
| COVPSU-01018 | male | Narathiwat | 1/12/2021 | 49 | 18.31 | AY.85 |
| COVPSU-01019 | female | Narathiwat | 3/12/2021 | 37 | 21.35 | AY.85 |
| COVPSU-01020 | female | Narathiwat | 3/12/2021 | 18 | 19.68 | AY.85 |
| COVPSU-01021 | male | Narathiwat | 3/12/2021 | 51 | 14.6 | AY.85 |
| COVPSU-01022 | female | Narathiwat | 4/12/2021 | 39 | 18.29 | AY.85 |
| COVPSU-01023 | female | Narathiwat | 4/12/2021 | 40 | 13.71 | AY.87 |
| COVPSU-01024 | female | Narathiwat | 30/11/2021 | 45 | 21.18 | AY.84 |
| COVPSU-01025 | female | Narathiwat | 1/12/2021 | 62 | 16.09 | AY.59 |
| COVPSU-01026 | female | Narathiwat | 2/12/2021 | 40 | 24.37 | AY.85 |
| COVPSU-01027 | male | Narathiwat | 3/12/2021 | 76 | 18.75 | AY.85 |
| COVPSU-01028 | male | Songkhla | 30/11/2021 | 37 | 18.84 | AY.85 |
| COVPSU-01029 | female | Songkhla | 2/12/2021 | 24 | 15.11 | AY.85 |
| COVPSU-01030 | male | Songkhla | 4/12/2021 | 1 | 14.44 | AY.85 |
| COVPSU-01031 | male | Songkhla | 6/12/2021 | 50 | 29.37 | AY.85 |
| COVPSU-01032 | male | Songkhla | 7/12/2021 | NA | 17.05 | AY.30 |
| COVPSU-01033 | female | Songkhla | 1/12/2021 | 38 | 16.03 | AY.85 |
| COVPSU-01034 | male | Songkhla | 3/12/2021 | 1 | 9.98 | AY.85 |
| COVPSU-01035 | female | Songkhla | 7/12/2021 | 63 | 13.35 | AY.85 |
| COVPSU-01036 | male | Songkhla | 8/12/2021 | 6 | 18 | AY.85 |
| COVPSU-01037 | male | Songkhla | 8/12/2021 | 40 | 12.53 | AY.85 |
| COVPSU-01038 | male | Songkhla | 8/12/2021 | 1 | 15.41 | AY.30 |
| COVPSU-01039 | male | Songkhla | 8/12/2021 | 76 | 18.36 | AY.30 |
| COVPSU-01041 | male | Songkhla | 6/12/2021 | 36 | 15.03 | AY.122 |
| COVPSU-01042 | male | Songkhla | 8/12/2021 | 40 | 15.37 | AY.30 |
| COVPSU-01043 | male | Songkhla | 9/12/2021 | 11 | 10.57 | AY.85 |
| COVPSU-01045 | male | Phatthalung | 8/12/2021 | 38 | 18.17 | AY.85 |
| COVPSU-01046 | female | Phatthalung | 8/12/2021 | 84 | 18.67 | AY.85 |
| COVPSU-01047 | female | Phatthalung | 8/12/2021 | 51 | 18.35 | AY.85 |
| COVPSU-01048 | male | Phatthalung | 9/12/2021 | 72 | 17.2 | AY.85 |
| COVPSU-01049 | female | Phatthalung | 9/12/2021 | 74 | 14.13 | AY.85 |
| COVPSU-01050 | male | Phatthalung | 9/12/2021 | 57 | 13.89 | AY.85 |
| COVPSU-01051 | male | Songkhla | 10/12/2021 | 56 | 18.84 | AY.30 |
| COVPSU-01052 | male | Songkhla | 10/12/2021 | 62 | 16.22 | AY.85 |
| COVPSU-01053 | male | Songkhla | 10/12/2021 | 67 | 17.43 | AY.85 |
| COVPSU-01054 | male | Songkhla | 10/12/2021 | 70 | 13.46 | AY.30 |
| COVPSU-01055 | male | Pattani | 10/12/2021 | 19 | 16.42 | B.1.617.2 |
| COVPSU-01056 | female | Songkhla | 2/12/2021 | 59 | 16 | AY.85 |
| COVPSU-01057 | female | Songkhla | 2/12/2021 | 65 | 15 | AY.85 |
| COVPSU-01058 | male | Songkhla | 2/12/2021 | 32 | 16.99 | AY.85 |
| COVPSU-01059 | female | Songkhla | 2/12/2021 | 10 | 12.72 | AY.85 |
| COVPSU-01060 | female | Yala | 8/12/2021 | 26 | 13.86 | AY.85 |
| COVPSU-01061 | female | Yala | 9/12/2021 | 8 | 12.11 | AY.85 |
| COVPSU-01062 | female | Yala | 8/12/2021 | 60 | 18.45 | AY.85 |
| COVPSU-01063 | male | Yala | 9/12/2021 | NA | 19.46 | AY.85 |
| COVPSU-01064 | female | Yala | 11/12/2021 | 41 | 16.2 | AY.85 |
| COVPSU-01065 | male | Yala | 11/12/2021 | 9 | 21.88 | AY.85 |
| COVPSU-01066 | female | Yala | 12/12/2021 | 70 | 23.1 | AY.85 |
| COVPSU-01067 | female | Yala | 12/12/2021 | 45 | 18 | AY.85 |
| COVPSU-01068 | female | Yala | 16/11/2021 | 71 | 16.08 | AY.85 |
| COVPSU-01069 | female | Yala | 5/12/2021 | 80 | 16.76 | AY.85 |
| COVPSU-01070 | female | Narathiwat | 10/12/2021 | 40 | 18.93 | B.1.617.2 |
| COVPSU-01071 | male | Narathiwat | 10/12/2021 | 60 | 20.74 | AY.59 |
| COVPSU-01072 | male | Narathiwat | 10/12/2021 | 22 | 27.31 | AY.85 |
| COVPSU-01073 | female | Narathiwat | 30/11/2021 | 71 | 18.32 | AY.85 |
| COVPSU-01074 | female | Narathiwat | 30/11/2021 | 31 | 18.52 | AY.59 |
| COVPSU-01075 | female | Narathiwat | 8/12/2021 | 67 | 17.43 | AY.85 |
| COVPSU-01076 | male | Narathiwat | 8/12/2021 | 8 | 20.41 | AY.91 |
| COVPSU-01077 | female | Narathiwat | 9/12/2021 | 25 | 22.99 | AY.85 |
| COVPSU-01078 | male | Narathiwat | 11/12/2021 | 20 | 15.43 | AY.85 |
| COVPSU-01079 | female | Narathiwat | 4/12/2021 | 49 | 18.27 | AY.59 |
| COVPSU-01080 | male | Narathiwat | 6/12/2021 | 18 | 21.95 | AY.85 |
| COVPSU-01081 | female | Narathiwat | 7/12/2021 | 23 | 22.52 | AY.85 |
| COVPSU-01082 | male | Narathiwat | 8/12/2021 | NA | 18.42 | AY.85 |
| COVPSU-01083 | male | Narathiwat | 9/12/2021 | 24 | 15.78 | AY.85 |
| COVPSU-01084 | male | Narathiwat | 9/12/2021 | 81 | 16.75 | AY.85 |
| COVPSU-01085 | male | Narathiwat | 9/12/2021 | 1 | 13.43 | AY.85 |
| COVPSU-01086 | male | Narathiwat | 9/12/2021 | NA | 8.29 | AY.85 |
| COVPSU-01087 | female | Narathiwat | 8/12/2021 | 100 | 11.84 | AY.85 |
| COVPSU-01088 | female | Narathiwat | 7/12/2021 | 40 | 21.06 | AY.61 |
| COVPSU-01089 | male | Narathiwat | 8/12/2021 | 50 | 13.92 | AY.85 |
| COVPSU-01090 | female | Narathiwat | 8/12/2021 | 58 | 22.84 | AY.61 |
| COVPSU-01091 | female | Narathiwat | 8/12/2021 | 15 | 17.4 | AY.85 |
| COVPSU-01092 | female | Narathiwat | 10/12/2021 | 51 | 19.49 | AY.85 |
| COVPSU-01093 | female | Narathiwat | 10/12/2021 | 74 | 29.12 | AY.85 |
| COVPSU-01094 | male | Narathiwat | 10/12/2021 | 15 | 21.22 | AY.85 |
| COVPSU-01095 | female | Narathiwat | 10/12/2021 | 31 | 12.67 | AY.85 |
| COVPSU-01096 | female | Narathiwat | 10/12/2021 | 73 | 16.95 | AY.85 |
| COVPSU-01098 | female | Songkhla | 15/12/2021 | NA | 15.26 | AY.85 |
| COVPSU-01099 | female | Songkhla | 15/12/2021 | NA | 23.56 | AY.39.1 |
| COVPSU-01101 | male | Songkhla | 15/12/2021 | NA | 16.16 | AY.85 |
| COVPSU-01102 | female | Songkhla | 15/12/2021 | NA | 14.76 | AY.45 |
| COVPSU-01103 | male | Songkhla | 15/12/2021 | NA | 19.05 | AY.30 |
| COVPSU-01104 | male | Songkhla | 15/12/2021 | NA | 18.62 | AY.85 |
| COVPSU-01105 | female | Songkhla | 15/12/2021 | NA | 12.32 | AY.85 |
| COVPSU-01106 | female | Songkhla | 15/12/2021 | NA | 11.01 | AY.30 |
| COVPSU-01107 | female | Songkhla | 16/12/2021 | NA | 30.4 | AY.85 |
| COVPSU-01108 | female | Songkhla | 16/12/2021 | NA | 21.54 | AY.85 |
| COVPSU-01109 | female | Songkhla | 16/12/2021 | NA | 16.3 | AY.30 |
| COVPSU-01110 | male | Songkhla | 16/12/2021 | NA | 14.41 | AY.85 |
| COVPSU-01111 | female | Songkhla | 16/12/2021 | NA | 9.93 | AY.91 |
| COVPSU-01112 | female | Songkhla | 16/12/2021 | NA | 14.88 | AY.85 |
| COVPSU-01113 | female | Songkhla | 16/12/2021 | NA | 20 | AY.85 |
| COVPSU-01114 | female | Songkhla | 13/12/2021 | 26 | 18.18 | AY.46.6 |
| COVPSU-01115 | female | Songkhla | 13/12/2021 | 47 | 15.58 | AY.85 |
| COVPSU-01116 | female | Songkhla | 14/12/2021 | 52 | 14.02 | AY.85 |
| COVPSU-01117 | male | Songkhla | 14/12/2021 | 47 | 14.44 | AY.85 |
| COVPSU-01118 | female | Songkhla | 15/12/2021 | NA | 13.5 | AY.85 |
| COVPSU-01119 | male | Songkhla | 9/12/2021 | 54 | 15.43 | AY.85 |
| COVPSU-01120 | female | Songkhla | 12/12/2021 | 20 | 13.91 | AY.85 |
| COVPSU-01121 | male | Songkhla | 16/12/2021 | 42 | 16.89 | AY.85 |
| COVPSU-01126 | female | Songkhla | 20/12/2021 | 53 | 22.11 | AY.85 |
| COVPSU-01127 | female | Songkhla | 12/12/2021 | 35 | 25.22 | AY.85 |
| COVPSU-01128 | female | Songkhla | 24/12/2021 | 3 | 18.62 | AY.85 |
| COVPSU-01129 | female | Songkhla | 27/12/2021 | 38 | 13.72 | AY.85 |
| COVPSU-01130 | male | Songkhla | 24/12/2021 | 63 | NA | AY.85 |
| COVPSU-01131 | female | Pattani | 2/12/2021 | 57 | 15.51 | AY.85 |
| COVPSU-01132 | male | Pattani | 3/12/2021 | 30 | 19.56 | AY.85 |
| COVPSU-01133 | female | Pattani | 4/12/2021 | 47 | 17.36 | AY.59 |
| COVPSU-01134 | female | Pattani | 5/12/2021 | 44 | 18.52 | AY.85 |
| COVPSU-01135 | female | Pattani | 6/12/2021 | 17 | 20.52 | AY.85 |
| COVPSU-01136 | female | Pattani | 9/12/2021 | 52 | 17.51 | AY.85 |
| COVPSU-01137 | female | Pattani | 10/12/2021 | 47 | 18.95 | AY.85 |
| COVPSU-01138 | female | Pattani | 12/12/2021 | 49 | 18.62 | AY.85 |
| COVPSU-01139 | female | Pattani | 13/12/2021 | 52 | 19.57 | AY.85 |
| COVPSU-01140 | male | Pattani | 15/12/2021 | 16 | 18.24 | AY.85 |
| COVPSU-01141 | male | Songkhla | 13/12/2021 | NA | 19.19 | AY.85 |
| COVPSU-01142 | male | Songkhla | 14/12/2021 | NA | 14.74 | AY.30 |
| COVPSU-01143 | male | Songkhla | 15/12/2021 | NA | 21.41 | AY.85 |
| COVPSU-01144 | male | Songkhla | 15/12/2021 | NA | 14.32 | AY.30 |
| COVPSU-01145 | female | Songkhla | 15/12/2021 | NA | 13.9 | AY.85 |
| COVPSU-01146 | male | Songkhla | 15/12/2021 | NA | 11.3 | AY.85 |
| COVPSU-01147 | male | Songkhla | 16/12/2021 | NA | 11.62 | AY.85 |
| COVPSU-01148 | female | Songkhla | 15/12/2021 | NA | 13.04 | AY.85 |
| COVPSU-01149 | male | Songkhla | 15/12/2021 | NA | 14.82 | AY.85 |
| COVPSU-01150 | female | Yala | 14/12/2021 | 90 | 13.71 | AY.32 |
| COVPSU-01151 | female | Phatthalung | 11/12/2021 | 10 | 13.23 | AY.30 |
| COVPSU-01153 | male | Phatthalung | 11/12/2021 | 57 | 15.28 | AY.85 |
| COVPSU-01154 | male | Phatthalung | 12/12/2021 | 14 | NA | AY.85 |
| COVPSU-01155 | female | Phatthalung | 12/12/2021 | 26 | NA | AY.85 |
| COVPSU-01156 | male | Phatthalung | 12/12/2021 | 54 | NA | AY.85 |
| COVPSU-01158 | female | Phatthalung | 13/12/2021 | 75 | NA | AY.85 |
| COVPSU-01159 | female | Pattani | 13/12/2021 | 38 | NA | AY.85 |
| COVPSU-01161 | female | Phatthalung | 14/12/2021 | 38 | NA | AY.85 |
| COVPSU-01162 | male | Phatthalung | 14/12/2021 | 16 | NA | AY.85 |
| COVPSU-01163 | female | Pattani | 14/12/2021 | 24 | NA | AY.85 |
| COVPSU-01164 | female | Pattani | 15/12/2021 | 25 | NA | AY.59 |
| COVPSU-01165 | female | Pattani | 15/12/2021 | 82 | NA | AY.85 |
| COVPSU-01166 | male | Pattani | 15/12/2021 | 16 | NA | AY.85 |
| COVPSU-01169 | male | Pattani | 16/12/2021 | 64 | NA | AY.85 |
| COVPSU-01170 | female | Phatthalung | 16/12/2021 | 13 | NA | AY.85 |
| COVPSU-01171 | male | Phatthalung | 16/12/2021 | 55 | NA | AY.85 |
| COVPSU-01172 | female | Phatthalung | 16/12/2021 | 27 | NA | AY.85 |
| COVPSU-01173 | male | Pattani | 17/12/2021 | 16 | NA | AY.85 |
| COVPSU-01176 | female | Phatthalung | 17/12/2021 | 66 | NA | AY.85 |
| COVPSU-01178 | female | Songkhla | 22/1/2022 | 59 | 13.24 | AY.85 |
| COVPSU-01179 | male | Songkhla | 17/12/2021 | 53 | 16.07 | AY.85 |
| COVPSU-01180 | male | Songkhla | 18/12/2021 | 75 | 12.86 | AY.85 |
| COVPSU-01190 | female | Pattani | 16/12/2021 | 59 | 19.1 | AY.30 |
| COVPSU-01191 | female | Pattani | 16/12/2021 | 67 | 13.3 | AY.85 |
| COVPSU-01192 | female | Pattani | 16/12/2021 | 43 | 14.41 | AY.85 |
| COVPSU-01193 | female | Pattani | 16/12/2021 | 2 | 10.32 | AY.85 |
| COVPSU-01194 | female | Pattani | 18/12/2021 | 48 | 20.72 | AY.85 |
| COVPSU-01195 | female | Pattani | 18/12/2021 | 42 | 12.07 | AY.59 |
| COVPSU-01196 | female | Pattani | 18/12/2021 | 43 | 27.86 | AY.85 |
| COVPSU-01197 | female | Pattani | 17/12/2021 | 22 | 15.74 | AY.85 |
| COVPSU-01198 | female | Pattani | 17/12/2021 | 19 | 14.19 | AY.85 |
| COVPSU-01199 | male | Pattani | 17/12/2021 | 71 | 18.65 | AY.85 |
| COVPSU-01200 | female | Pattani | 19/12/2021 | 64 | 14.2 | AY.30 |
| COVPSU-01201 | female | Pattani | 19/12/2021 | 24 | 12.71 | AY.85 |
| COVPSU-01202 | male | Pattani | 19/12/2021 | 55 | 16.88 | AY.85 |
| COVPSU-01203 | male | Pattani | 19/12/2021 | 51 | 18.27 | AY.85 |
| COVPSU-01204 | male | Yala | 17/12/2021 | 26 | 16.78 | AY.85 |
| COVPSU-01205 | female | Yala | 17/12/2021 | 10 | 15.95 | AY.85 |
| COVPSU-01206 | male | Songkhla | 20/12/2021 | NA | 10.54 | AY.85 |
| COVPSU-01207 | female | Yala | 16/12/2021 | 27 | 15.38 | AY.85 |
| COVPSU-01208 | male | Yala | 16/12/2021 | 48 | 16.92 | AY.85 |
| COVPSU-01209 | female | Yala | 17/12/2021 | 14 | 16.57 | AY.85 |
| COVPSU-01210 | male | Narathiwat | 14/12/2021 | 48 | 13.28 | AY.85 |
| COVPSU-01211 | female | Narathiwat | 14/12/2021 | NA | 8.3 | AY.59 |
| COVPSU-01212 | female | Narathiwat | 16/12/2021 | 54 | 14.09 | AY.79 |
| COVPSU-01213 | male | Narathiwat | 16/12/2021 | 34 | 13.74 | AY.79 |
| COVPSU-01214 | female | Narathiwat | 15/12/2021 | NA | 14.56 | AY.59 |
| COVPSU-01215 | female | Narathiwat | 20/12/2021 | 63 | 11.03 | AY.85 |
| COVPSU-01216 | female | Narathiwat | 13/12/2021 | 66 | 19.06 | AY.59 |
| COVPSU-01217 | male | Narathiwat | 13/12/2021 | 70 | 16.17 | AY.59 |
| COVPSU-01218 | male | Narathiwat | 14/12/2021 | 82 | 16.83 | AY.59 |
| COVPSU-01219 | male | Narathiwat | 17/12/2021 | 51 | 21.27 | AY.59 |
| COVPSU-01220 | female | Narathiwat | 17/12/2021 | 40 | 19.53 | AY.59 |
| COVPSU-01221 | female | Narathiwat | 13/12/2021 | 21 | 20.69 | AY.85 |
| COVPSU-01222 | male | Narathiwat | 12/12/2021 | 87 | 23.71 | AY.85 |
| COVPSU-01223 | male | Narathiwat | 15/12/2021 | 24 | 16.97 | AY.85 |
| COVPSU-01224 | male | Narathiwat | 19/12/2021 | 50 | 23.97 | AY.85 |
| COVPSU-01225 | female | Songkhla | 20/12/2021 | 31 | 29.15 | AY.85 |
| COVPSU-01226 | male | Songkhla | 17/12/2021 | NA | 18.36 | AY.85 |
| COVPSU-01227 | male | Songkhla | 17/12/2021 | NA | 13.75 | AY.30 |
| COVPSU-01228 | female | Songkhla | 14/12/2021 | NA | 10.29 | AY.85 |
| COVPSU-01229 | male | Songkhla | 18/12/2021 | NA | 13.97 | AY.30 |
| COVPSU-01230 | female | Songkhla | 14/12/2021 | NA | 13.49 | AY.30 |
| COVPSU-01232 | male | Songkhla | 17/12/2021 | 39 | 15.31 | AY.85 |
| COVPSU-01233 | male | Songkhla | 20/12/2021 | NA | 13.52 | AY.85 |
| COVPSU-01234 | male | Songkhla | 23/12/2021 | 24 | 11.62 | AY.85 |
| COVPSU-01235 | female | Songkhla | 16/12/2021 | 46 | 13.43 | AY.85 |
| COVPSU-01236 | male | Songkhla | 20/12/2021 | 39 | 15.59 | AY.85 |
| COVPSU-01238 | male | Pattani | 16/12/2021 | 43 | 20.23 | AY.85 |
| COVPSU-01239 | male | Pattani | 19/12/2021 | 71 | 20 | AY.85 |
| COVPSU-01240 | female | Pattani | 20/12/2021 | 41 | 24.85 | AY.85 |
| COVPSU-01241 | female | Songkhla | 21/12/2021 | NA | 16.27 | AY.85 |
| COVPSU-01242 | male | Songkhla | 21/12/2021 | NA | 14.98 | AY.85 |
| COVPSU-01243 | male | Songkhla | 21/12/2021 | NA | 12.98 | AY.30 |
| COVPSU-01244 | female | Songkhla | 22/12/2021 | NA | 15.32 | AY.85 |
| COVPSU-01245 | female | Songkhla | 22/12/2021 | NA | 21.25 | AY.85 |
| COVPSU-01246 | male | Songkhla | 22/12/2021 | NA | 16.73 | AY.85 |
| COVPSU-01247 | female | Songkhla | 22/12/2021 | NA | 13.89 | AY.85 |
| COVPSU-01248 | female | Songkhla | 22/12/2021 | NA | 13.34 | AY.85 |
| COVPSU-01249 | female | Songkhla | 24/12/2021 | NA | 14.22 | AY.59 |
| COVPSU-01250 | female | Songkhla | 24/12/2021 | NA | 11.77 | AY.85 |
| COVPSU-01251 | female | Songkhla | 24/12/2021 | NA | 15.63 | AY.85 |
| COVPSU-01252 | female | Songkhla | 24/12/2021 | NA | 15.76 | AY.85 |
| COVPSU-01253 | male | Songkhla | 24/12/2021 | NA | 17.38 | AY.85 |
| COVPSU-01254 | female | Songkhla | 17/12/2021 | NA | 14.96 | AY.85 |
| COVPSU-01255 | female | Songkhla | 17/12/2021 | NA | 19.49 | AY.85 |
| COVPSU-01256 | female | Songkhla | 18/12/2021 | NA | 15.31 | AY.85 |
| COVPSU-01257 | female | Songkhla | 21/12/2021 | NA | 19.4 | AY.85 |
| COVPSU-01258 | female | Songkhla | 21/12/2021 | NA | 11.77 | AY.85 |
| COVPSU-01259 | female | Songkhla | 21/12/2021 | NA | 19.46 | AY.85 |
| COVPSU-01260 | female | Yala | 10/12/2021 | 77 | 21.69 | AY.85 |
| COVPSU-01261 | female | Songkhla | 17/12/2021 | 57 | NA | AY.85 |
| COVPSU-01263 | female | Phatthalung | 19/12/2021 | 49 | NA | AY.85 |
| COVPSU-01264 | male | Songkhla | 20/12/2021 | 20 | NA | AY.85 |
| COVPSU-01265 | male | Songkhla | 20/12/2021 | 31 | NA | AY.85 |
| COVPSU-01266 | male | Songkhla | 20/12/2021 | 18 | NA | AY.85 |
| COVPSU-01269 | female | Phatthalung | 21/12/2021 | 60 | NA | AY.85 |
| COVPSU-01270 | female | Pattani | 21/12/2021 | 53 | NA | AY.85 |
| COVPSU-01271 | male | Pattani | 21/12/2021 | 32 | NA | AY.30 |
| COVPSU-01272 | male | Phatthalung | 21/12/2021 | 57 | NA | AY.85 |
| COVPSU-01273 | female | Phatthalung | 21/12/2021 | 25 | NA | AY.85 |
| COVPSU-01275 | female | Songkhla | 21/12/2021 | 41 | 22.79 | AY.59 |
| COVPSU-01276 | female | Songkhla | 21/12/2021 | 30 | 14.95 | AY.85 |
| COVPSU-01277 | male | Phatthalung | 23/12/2021 | 89 | NA | AY.30 |
| COVPSU-01278 | male | Pattani | 23/12/2021 | 16 | NA | AY.85 |
| COVPSU-01280 | female | Phatthalung | 23/12/2021 | 3 | NA | AY.85 |
| COVPSU-01281 | female | Phatthalung | 23/12/2021 | 86 | NA | AY.85 |
| COVPSU-01282 | female | Phatthalung | 24/12/2021 | 20 | NA | AY.85 |
| COVPSU-01284 | female | Phatthalung | 24/12/2021 | 20 | NA | AY.85 |
| COVPSU-01286 | female | Songkhla | 23/12/2021 | 32 | 17.42 | AY.85 |
| COVPSU-01287 | female | Songkhla | 25/12/2021 | 63 | 12.51 | AY.85 |
| COVPSU-01288 | male | Songkhla | 27/12/2021 | NA | 11.72 | AY.30 |
| COVPSU-01289 | male | Songkhla | 27/12/2021 | NA | 13.36 | AY.30 |
| COVPSU-01290 | male | Songkhla | 27/12/2021 | NA | 22.2 | AY.85 |
| COVPSU-01291 | male | Songkhla | 27/12/2021 | NA | 13.37 | AY.85 |
| COVPSU-01292 | male | Songkhla | 27/12/2021 | NA | 12.79 | AY.85 |
| COVPSU-01293 | male | Songkhla | 27/12/2021 | NA | 13.14 | AY.85 |
| COVPSU-01294 | female | Songkhla | 27/12/2021 | NA | 23.48 | AY.85 |
| COVPSU-01295 | male | Songkhla | 27/12/2021 | NA | 17.19 | AY.85 |
| COVPSU-01296 | female | Songkhla | 27/12/2021 | 31 | 15.57 | AY.30 |
| COVPSU-01297 | female | Songkhla | 27/12/2021 | 27 | 18.05 | AY.85 |
| COVPSU-01298 | female | Pattani | 27/12/2021 | 52 | 16.17 | AY.85 |
| COVPSU-01299 | female | Songkhla | 25/12/2021 | 31 | 23.07 | AY.85 |
| COVPSU-01300 | male | Narathiwat | 22/12/2021 | 49 | 18.89 | AY.85 |
| COVPSU-01301 | male | Narathiwat | 28/12/2021 | 19 | 19.31 | AY.85 |
| COVPSU-01302 | female | Narathiwat | 21/12/2021 | 35 | 15.15 | AY.85 |
| COVPSU-01303 | male | Narathiwat | 23/12/2021 | 32 | 18.23 | AY.61 |
| COVPSU-01304 | male | Narathiwat | 26/12/2021 | 32 | 21.33 | AY.85 |
| COVPSU-01305 | male | Narathiwat | 24/12/2021 | 31 | 12.62 | AY.59 |
| COVPSU-01307 | male | Narathiwat | 21/12/2021 | 21 | 23.06 | AY.85 |
| COVPSU-01308 | male | Narathiwat | 21/12/2021 | 22 | 25.21 | AY.85 |
| COVPSU-01309 | male | Narathiwat | 21/12/2021 | 40 | 20.63 | AY.85 |
| COVPSU-01310 | female | Narathiwat | 21/12/2021 | 34 | 20.01 | AY.85 |
| COVPSU-01311 | female | Narathiwat | 23/12/2021 | 41 | 18.66 | AY.85 |
| COVPSU-01312 | male | Narathiwat | 24/12/2021 | 72 | 19.49 | AY.85 |
| COVPSU-01313 | male | Narathiwat | 24/12/2021 | 7 | 18.55 | AY.85 |
| COVPSU-01314 | female | Narathiwat | 24/12/2021 | 5 | 18.86 | AY.85 |
| COVPSU-01315 | male | Narathiwat | 25/12/2021 | 39 | 22.5 | AY.85 |
| COVPSU-01316 | male | Narathiwat | 24/12/2021 | 28 | 23.24 | AY.85 |
| COVPSU-01317 | female | Songkhla | 28/12/2021 | 33 | 21.62 | AY.85 |
| COVPSU-01318 | male | Yala | 14/11/2021 | 89 | 15.36 | AY.85 |
| COVPSU-01319 | female | Songkhla | 30/12/2021 | NA | 17.45 | AY.85 |
| COVPSU-01320 | male | Songkhla | 29/12/2021 | NA | 17.73 | AY.85 |
| COVPSU-01321 | male | Songkhla | 29/12/2021 | NA | 14.07 | AY.85 |
| COVPSU-01322 | female | Songkhla | 27/12/2021 | NA | 16.72 | AY.85 |
| COVPSU-01323 | female | Songkhla | 28/12/2021 | NA | 19.9 | AY.85 |
| COVPSU-01324 | female | Songkhla | 28/12/2021 | NA | 22.17 | AY.85 |
| COVPSU-01325 | female | Songkhla | 29/12/2021 | NA | 12.5 | AY.85 |
| COVPSU-01326 | female | Songkhla | 30/12/2021 | NA | 22.98 | AY.85 |
| COVPSU-01327 | male | Songkhla | 29/12/2021 | NA | 21.17 | AY.85 |
| COVPSU-01330 | female | Phatthalung | 25/12/2021 | 84 | NA | AY.85 |
| COVPSU-01331 | female | Phatthalung | 25/12/2021 | 28 | NA | AY.85 |
| COVPSU-01332 | female | Phatthalung | 26/12/2021 | 89 | NA | AY.85 |
| COVPSU-01333 | male | Phatthalung | 26/12/2021 | 25 | NA | AY.85 |
| COVPSU-01334 | female | Phatthalung | 26/12/2021 | 34 | NA | AY.85 |
| COVPSU-01338 | male | Pattani | 27/12/2021 | 33 | NA | AY.85 |
| COVPSU-01340 | female | Phatthalung | 27/12/2021 | 74 | NA | AY.85 |
| COVPSU-01341 | female | Phatthalung | 28/12/2021 | 37 | NA | AY.30 |
| COVPSU-01342 | female | Phatthalung | 28/12/2021 | 68 | NA | AY.30 |
| COVPSU-01343 | male | Pattani | 28/12/2021 | 43 | NA | AY.85 |
| COVPSU-01344 | female | Phatthalung | 29/12/2021 | 56 | NA | AY.85 |
| COVPSU-01345 | male | Phatthalung | 29/12/2021 | 42 | NA | AY.85 |
| COVPSU-01346 | female | Phatthalung | 29/12/2021 | 49 | NA | AY.85 |
| COVPSU-01347 | female | Pattani | 30/12/2021 | 37 | NA | AY.85 |
| COVPSU-01348 | female | Phatthalung | 30/12/2021 | 28 | NA | AY.85 |
| COVPSU-01350 | male | Songkhla | 2/1/2022 | NA | 20.12 | AY.85 |
| COVPSU-01351 | male | Songkhla | 2/1/2022 | NA | 17.71 | AY.30 |
| COVPSU-01352 | male | Songkhla | 2/1/2022 | NA | 16.96 | AY.85 |
| COVPSU-01353 | male | Songkhla | 2/1/2022 | NA | 17.93 | AY.85 |
| COVPSU-01354 | female | Songkhla | 2/1/2022 | NA | 18.32 | AY.85 |
| COVPSU-01355 | female | Songkhla | 29/12/2021 | 36 | 15.84 | AY.85 |
| COVPSU-01356 | female | Songkhla | 2/1/2022 | NA | 15.58 | AY.85 |
| COVPSU-01357 | female | Songkhla | 31/12/2021 | NA | 20.29 | AY.85 |
| COVPSU-01358 | male | Yala | 16/12/2021 | NA | 13.5 | AY.85 |
| COVPSU-01359 | male | Yala | 18/12/2021 | NA | 12.97 | AY.85 |
| COVPSU-01360 | male | Yala | 19/12/2021 | NA | 14.34 | AY.85 |
| COVPSU-01361 | male | Yala | 20/12/2021 | NA | 16.47 | AY.85 |
| COVPSU-01362 | male | Yala | 20/12/2021 | NA | 14.16 | AY.85 |
| COVPSU-01363 | female | Yala | 22/12/2021 | NA | 13.68 | AY.32 |
| COVPSU-01364 | female | Yala | 22/12/2021 | NA | 11.87 | AY.85 |
| COVPSU-01365 | male | Yala | 24/12/2021 | NA | 13.48 | AY.85 |
| COVPSU-01366 | male | Yala | 24/12/2021 | NA | 12.1 | AY.85 |
| COVPSU-01367 | female | Yala | 27/12/2021 | NA | 13.48 | AY.85 |
| COVPSU-01368 | female | Yala | 27/12/2021 | NA | 13.6 | AY.85 |
| COVPSU-01369 | female | Yala | 28/12/2021 | NA | 17.39 | AY.85 |
| COVPSU-01370 | female | Yala | 29/12/2021 | NA | 14.28 | AY.85 |
| COVPSU-01371 | female | Songkhla | 31/12/2021 | NA | 14.04 | AY.85 |
| COVPSU-01372 | female | Songkhla | 1/1/2022 | NA | 16.66 | AY.85 |
| COVPSU-01373 | female | Songkhla | 1/1/2022 | NA | 16.2 | AY.85 |
| COVPSU-01374 | male | Songkhla | 2/1/2022 | NA | 13.03 | AY.85 |
| COVPSU-01375 | male | Narathiwat | 31/12/2021 | 24 | 18.68 | AY.85 |
| COVPSU-01376 | male | Narathiwat | 29/12/2021 | 48 | 22.33 | AY.85 |
| COVPSU-01377 | female | Narathiwat | 29/12/2021 | 31 | 17.71 | AY.85 |
| COVPSU-01378 | female | Narathiwat | 31/12/2021 | 27 | 12.19 | AY.85 |
| COVPSU-01379 | female | Narathiwat | 31/12/2021 | 82 | 17.58 | AY.85 |
| COVPSU-01380 | male | Narathiwat | 3/1/2022 | 31 | 14.38 | AY.85 |
| COVPSU-01381 | female | Narathiwat | 27/12/2021 | 45 | NA | AY.85 |
| COVPSU-01382 | female | Narathiwat | 30/12/2021 | 37 | NA | AY.79 |
| COVPSU-01383 | female | Narathiwat | 31/12/2021 | 11 | NA | AY.85 |
| COVPSU-01384 | female | Songkhla | 5/1/2022 | NA | 14.44 | AY.85 |
| COVPSU-01385 | female | Songkhla | 5/1/2022 | NA | 11.25 | AY.85 |
| COVPSU-01386 | female | Songkhla | 5/1/2022 | NA | 13.88 | AY.30 |
| COVPSU-01387 | female | Yala | 4/1/2022 | NA | 25.1 | AY.85 |
| COVPSU-01388 | male | Yala | 4/1/2022 | NA | 13.18 | AY.85 |
| COVPSU-01389 | male | Songkhla | 4/1/2022 | NA | 22.21 | AY.85 |
| COVPSU-01390 | male | Songkhla | 5/1/2022 | NA | 21.66 | AY.85 |
| COVPSU-01391 | male | Songkhla | 4/1/2022 | NA | 15.12 | AY.85 |
| COVPSU-01392 | female | Songkhla | 23/1/2022 | 20 | 18.31 | AY.85 |
| COVPSU-01393 | male | Pattani | 5/1/2022 | 19 | 21.51 | BA.1.1 |
| COVPSU-01394 | male | Pattani | 5/1/2022 | 16 | 15.3 | BA.1.1 |
| COVPSU-01395 | female | Yala | 5/1/2022 | 25 | 18.32 | BA.1 |
| COVPSU-01396 | male | Yala | 5/1/2022 | 29 | 17.17 | BA.1.1 |
| COVPSU-01397 | female | Songkhla | 5/1/2022 | 27 | 19.26 | BA.1.1 |
| COVPSU-01398 | female | Songkhla | 5/1/2022 | 6 | 16 | BA.1 |
| COVPSU-01399 | female | Songkhla | 5/1/2022 | 20 | 17.22 | BA.1.1 |
| COVPSU-01400 | male | Songkhla | 3/1/2022 | 49 | 14.41 | AY.30 |
| COVPSU-01401 | female | Songkhla | 4/1/2022 | 39 | 16.24 | BA.1 |
| COVPSU-01402 | female | Songkhla | 3/1/2022 | 34 | 16.1 | AY.85 |
| COVPSU-01403 | female | Songkhla | 3/1/2022 | 24 | 15.51 | BA.1.1 |
| COVPSU-01404 | male | Songkhla | 5/1/2022 | 12 | 14.08 | AY.85 |
| COVPSU-01405 | female | Songkhla | 6/1/2022 | 26 | 16.25 | BA.1 |
| COVPSU-01406 | male | Songkhla | 7/1/2022 | NA | 21.44 | BA.1 |
| COVPSU-01407 | male | Songkhla | 7/1/2022 | NA | 14.21 | BA.1.1 |
| COVPSU-01408 | male | Songkhla | 7/1/2022 | NA | 10.05 | BA.1 |
| COVPSU-01409 | female | Songkhla | 7/1/2022 | NA | 17.94 | BA.1.1 |
| COVPSU-01410 | female | Songkhla | 7/1/2022 | NA | 12.9 | BA.1.1 |
| COVPSU-01411 | male | Songkhla | 7/1/2022 | NA | 16.59 | BA.1 |
| COVPSU-01412 | female | Songkhla | 7/1/2022 | NA | 16.23 | BA.1.1 |
| COVPSU-01413 | female | Songkhla | 7/1/2022 | NA | 32.21 | BA.1.1 |
| COVPSU-01414 | female | Songkhla | 7/1/2022 | NA | 12.55 | AY.85 |
| COVPSU-01415 | female | Songkhla | 7/1/2022 | NA | 10.47 | BA.1.1 |
| COVPSU-01416 | male | Songkhla | 7/1/2022 | NA | 23.03 | BA.1.1 |
| COVPSU-01417 | male | Songkhla | 5/1/2022 | NA | 16.12 | AY.30 |
| COVPSU-01418 | male | Songkhla | 5/1/2022 | NA | 15.1 | BA.1.1 |
| COVPSU-01419 | female | Songkhla | 4/1/2022 | NA | 15.97 | BA.1 |
| COVPSU-01420 | female | Songkhla | 4/1/2022 | NA | 17.81 | BA.1.1 |
| COVPSU-01421 | female | Songkhla | 4/1/2022 | NA | 19.73 | BA.1 |
| COVPSU-01422 | female | Songkhla | 4/1/2022 | NA | 13.67 | BA.1.1 |
| COVPSU-01423 | male | Songkhla | 4/1/2022 | NA | 16.05 | BA.1.1 |
| COVPSU-01424 | female | Songkhla | 4/1/2022 | NA | 14.87 | AY.85 |
| COVPSU-01425 | male | Songkhla | 4/1/2022 | NA | 21.03 | BA.1.1 |
| COVPSU-01426 | female | Songkhla | 4/1/2022 | NA | 17.54 | BA.1 |
| COVPSU-01427 | female | Songkhla | 4/1/2022 | NA | 13.29 | AY.85 |
| COVPSU-01428 | male | Songkhla | 4/1/2022 | NA | 17.16 | BA.1.1 |
| COVPSU-01429 | male | Songkhla | 4/1/2022 | NA | 19.51 | BA.1 |
| COVPSU-01430 | male | Songkhla | 4/1/2022 | NA | 14.76 | BA.1.1 |
| COVPSU-01431 | female | Songkhla | 4/1/2022 | NA | 17.26 | BA.1.1 |
| COVPSU-01432 | female | Songkhla | 30/12/2021 | NA | 15.52 | AY.85 |
| COVPSU-01433 | female | Songkhla | 6/1/2022 | NA | 21.08 | BA.1.1 |
| COVPSU-01434 | female | Songkhla | 6/1/2022 | NA | 17.69 | BA.2 |
| COVPSU-01435 | female | Songkhla | 6/1/2022 | NA | 21.48 | BA.1.1 |
| COVPSU-01436 | male | Songkhla | 6/1/2022 | NA | 16.07 | AY.85 |
| COVPSU-01437 | female | Songkhla | 6/1/2022 | NA | 21.25 | BA.1.1 |
| COVPSU-01438 | female | Songkhla | 6/1/2022 | NA | 22.24 | BA.1.1 |
| COVPSU-01439 | female | Songkhla | 1/1/2022 | 33 | 17.32 | BA.1 |
| COVPSU-01440 | female | Songkhla | 4/1/2022 | 32 | 15.58 | BA.1 |
| COVPSU-01441 | female | Songkhla | 4/1/2022 | 18 | 25.12 | BA.1 |
| COVPSU-01442 | female | Songkhla | 4/1/2022 | 15 | 23.16 | BA.1 |
| COVPSU-01443 | male | Pattani | 24/12/2021 | 62 | 24.85 | AY.85 |
| COVPSU-01444 | female | Pattani | 25/12/2021 | 49 | 21.63 | AY.85 |
| COVPSU-01445 | male | Pattani | 28/12/2021 | 11 | 19.65 | AY.85 |
| COVPSU-01448 | male | Pattani | 6/1/2022 | NA | 17.59 | BA.1.1 |
| COVPSU-01449 | male | Pattani | 6/1/2022 | NA | 20.38 | BA.1.1 |
| COVPSU-01450 | male | Pattani | 6/1/2022 | NA | 17.52 | BA.1 |
| COVPSU-01451 | male | Yala | 6/1/2022 | NA | 22.56 | BA.1.1 |
| COVPSU-01452 | male | Yala | 6/1/2022 | NA | 19.59 | BA.1.1 |
| COVPSU-01453 | female | Songkhla | 6/1/2022 | 48 | 18.59 | AY.85 |
| COVPSU-01455 | female | Songkhla | 8/1/2022 | NA | 15.49 | AY.85 |
| COVPSU-01456 | female | Songkhla | 8/1/2022 | NA | 30.43 | BA.1.1 |
| COVPSU-01457 | female | Songkhla | 8/1/2022 | NA | 12.55 | AY.85 |
| COVPSU-01458 | female | Songkhla | 8/1/2022 | NA | 13.64 | AY.85 |
| COVPSU-01460 | female | Songkhla | 7/1/2022 | 38 | 25.02 | BA.1 |
| COVPSU-01461 | male | Songkhla | 7/1/2022 | 52 | 25.57 | BA.1 |
| COVPSU-01462 | male | Songkhla | 7/1/2022 | 24 | 16.43 | BA.1.1 |
| COVPSU-01463 | male | Songkhla | 7/1/2022 | 56 | 15.99 | BA.1.1 |
| COVPSU-01464 | female | Songkhla | 7/1/2022 | NA | 22.12 | BA.1 |
| COVPSU-01465 | female | Songkhla | 7/1/2022 | 27 | 20.34 | BA.1 |
| COVPSU-01466 | male | Songkhla | 7/1/2022 | 25 | 24.77 | BA.1 |
| COVPSU-01467 | male | Songkhla | 7/1/2022 | 71 | 18.13 | AY.30 |
| COVPSU-01468 | female | Songkhla | 4/1/2022 | NA | 13.72 | BA.1.1 |
| COVPSU-01469 | male | Songkhla | 4/1/2022 | NA | 18.28 | BA.1 |
| COVPSU-01470 | female | Songkhla | 4/1/2022 | NA | 13.16 | AY.85 |
| COVPSU-01471 | female | Songkhla | 6/1/2022 | NA | 16.79 | BA.1.1 |
| COVPSU-01472 | male | Songkhla | 6/1/2022 | NA | 17.05 | BA.1.1 |
| COVPSU-01473 | male | Songkhla | 6/1/2022 | NA | 15.92 | BA.1.1 |
| COVPSU-01475 | female | Songkhla | 2/1/2022 | 9 | 19.5 | AY.85 |
| COVPSU-01476 | female | Phatthalung | 2/1/2022 | 19 | 14.51 | AY.85 |
| COVPSU-01477 | male | Phatthalung | 2/1/2022 | 56 | 15.34 | AY.85 |
| COVPSU-01479 | female | Phatthalung | 4/1/2022 | 80 | N/A | AY.85 |
| COVPSU-01480 | female | Phatthalung | 4/1/2022 | 11 | N/A | AY.30 |
| COVPSU-01490 | male | Pattani | 6/1/2022 | 15 | NA | AY.85 |
| COVPSU-01491 | female | Pattani | 5/1/2022 | 20 | NA | AY.85 |
| COVPSU-01492 | female | Phatthalung | 6/1/2022 | 28 | NA | AY.85 |
| COVPSU-01494 | female | Phatthalung | 7/1/2022 | 18 | NA | AY.85 |
| COVPSU-01496 | female | Songkhla | 1/1/2022 | 1 | 16.15 | AY.85 |
| COVPSU-01498 | male | Songkhla | 3/1/2022 | 57 | 20.11 | BA.1.1 |
| COVPSU-01499 | male | Songkhla | 3/1/2022 | 1 | 14.49 | BA.1 |
| COVPSU-01500 | female | Songkhla | 4/1/2022 | 41 | 18.27 | BA.1 |
| COVPSU-01501 | female | Songkhla | 4/1/2022 | 20 | 17.96 | BA.1 |
| COVPSU-01502 | female | Songkhla | 4/1/2022 | 10 | 21.01 | AY.30 |
| COVPSU-01503 | female | Songkhla | 4/1/2022 | 34 | 23.69 | AY.85 |
| COVPSU-01504 | male | Songkhla | 4/1/2022 | 34 | 24.16 | BA.1.1 |
| COVPSU-01505 | female | Songkhla | 6/1/2022 | 20 | 15.13 | BA.1 |
| COVPSU-01506 | female | Songkhla | 8/1/2022 | NA | 14.58 | AY.85 |
| COVPSU-01507 | male | Songkhla | 31/1/2022 | NA | 11.49 | BA.1.1 |
| COVPSU-01697 | male | Yala | 14/2/2022 | NA | NA | BA.1.1 |
| COVPSU-01698 | female | Yala | 14/2/2022 | NA | NA | BA.1.1 |
| COVPSU-01699 | female | Yala | 14/2/2022 | NA | NA | BA.2 |
| COVPSU-01700 | female | Yala | 14/2/2022 | NA | NA | BA.1.1 |
| COVPSU-01701 | female | Yala | 14/2/2022 | NA | NA | BA.1.1 |
| COVPSU-01702 | female | Yala | 15/2/2022 | NA | NA | BA.2 |
| COVPSU-01703 | female | Yala | 15/2/2022 | NA | NA | BA.2 |
| COVPSU-01704 | female | Yala | 15/2/2022 | NA | NA | BA.1.1 |
| COVPSU-01705 | female | Yala | 16/2/2022 | NA | NA | BA.2 |
| COVPSU-01706 | male | Yala | 16/2/2022 | NA | NA | BA.2 |
| COVPSU-01707 | female | Yala | 17/2/2022 | NA | NA | BA.1.1 |
| COVPSU-01708 | male | Yala | 18/2/2022 | NA | NA | BA.1.1 |
| COVPSU-01709 | female | Yala | 19/2/2022 | NA | NA | BA.2 |
| COVPSU-01710 | male | Yala | 19/2/2022 | NA | NA | BA.2 |
| COVPSU-01711 | female | Yala | 20/2/2022 | NA | NA | BA.1.1 |
| COVPSU-01712 | male | Yala | 20/2/2022 | NA | NA | BA.2 |
| COVPSU-01713 | male | Songkhla | 18/2/2022 | NA | 22.59 | BA.1 |
| COVPSU-01714 | male | Songkhla | 18/2/2022 | NA | 21.28 | BA.1 |
| COVPSU-01715 | male | Songkhla | 18/2/2022 | NA | 21.1 | BA.1.1 |
| COVPSU-01716 | male | Songkhla | 18/2/2022 | NA | 24.97 | BA.2 |
| COVPSU-01717 | male | Songkhla | 18/2/2022 | NA | 21.63 | BA.1 |
| COVPSU-01718 | male | Songkhla | 18/2/2022 | NA | 20.65 | BA.1 |
| COVPSU-01719 | male | Songkhla | 18/2/2022 | NA | 22.49 | BA.1 |
| COVPSU-01720 | female | Yala | 17/2/2022 | 37 | 17.62 | BA.2 |
| COVPSU-01721 | female | Yala | 16/2/2022 | 36 | 19.71 | BA.2 |
| COVPSU-01722 | male | Songkhla | 20/2/2022 | NA | 17.23 | BA.1.1 |
| COVPSU-01723 | female | Songkhla | 20/2/2022 | NA | 14.9 | BA.2 |
| COVPSU-01724 | female | Songkhla | 20/2/2022 | NA | 16.24 | BA.1 |
| COVPSU-01725 | female | Songkhla | 20/2/2022 | NA | 15.22 | BA.1.1 |
| COVPSU-01726 | female | Songkhla | 20/2/2022 | NA | 16.64 | BA.1 |
| COVPSU-01727 | female | Songkhla | 20/2/2022 | NA | 14.29 | BA.2 |
| COVPSU-01728 | male | Songkhla | 20/2/2022 | NA | 16.11 | BA.1.1 |
| COVPSU-01729 | female | Songkhla | 20/2/2022 | NA | 21.78 | BA.2 |
| COVPSU-01730 | male | Songkhla | 20/2/2022 | NA | 19.21 | BA.1.1 |
| COVPSU-01731 | male | Songkhla | 20/2/2022 | NA | 16.68 | BA.1.1 |
| COVPSU-01732 | female | Songkhla | 20/2/2022 | NA | 13.84 | BA.2 |
| COVPSU-01735 | female | Yala | 19/2/2022 | 35 | 16.51 | BA.2 |
| COVPSU-01736 | male | Yala | 17/2/2022 | 41 | 27.11 | BA.2 |
| COVPSU-01737 | female | Yala | 19/2/2022 | 35 | 16.93 | BA.2 |
| COVPSU-01738 | female | Yala | 21/2/2022 | 36 | 18.87 | BA.2 |
| COVPSU-01739 | female | Yala | 16/2/2022 | 3 | 23.16 | BA.2 |
| COVPSU-01740 | male | Yala | 16/2/2022 | 1 | 16.79 | BA.2 |
| COVPSU-01741 | female | Yala | 18/2/2022 | 29 | 23.81 | BA.2 |
| COVPSU-01742 | male | Yala | 19/2/2022 | 52 | 15.62 | BA.2 |
| COVPSU-01743 | male | Yala | 16/2/2022 | 57 | 16.13 | BA.1.1 |
| COVPSU-01744 | male | Yala | 17/2/2022 | 75 | 16.01 | BA.2 |
| COVPSU-01745 | male | Yala | 19/2/2022 | 45 | 10.01 | BA.2 |
| COVPSU-01746 | female | Songkhla | 19/2/2022 | 74 | 24.35 | BA.1 |
| COVPSU-01747 | male | Yala | 14/2/2022 | 37 | 17.13 | BA.2 |
| COVPSU-01748 | male | Yala | 14/2/2022 | 17 | 20.68 | BA.2 |
| COVPSU-01749 | male | Yala | 14/2/2022 | 19 | 19.36 | BA.2 |
| COVPSU-01750 | female | Yala | 14/2/2022 | 23 | 15.64 | BA.2 |
| COVPSU-01751 | male | Yala | 14/2/2022 | 20 | 15.02 | BA.2 |
| COVPSU-01752 | female | Yala | 15/2/2022 | 46 | 16.68 | BA.2 |
| COVPSU-01753 | male | Yala | 17/2/2022 | 69 | 15.54 | BA.2 |
| COVPSU-01754 | male | Yala | 17/2/2022 | 23 | 17.07 | BA.1.1 |
| COVPSU-01755 | female | Yala | 19/2/2022 | 23 | 12.45 | BA.2 |
| COVPSU-01756 | male | Songkhla | 15/2/2022 | 32 | 14.06 | BA.1.1 |
| COVPSU-01757 | male | Songkhla | 15/2/2022 | 43 | 18.19 | BA.2 |
| COVPSU-01758 | female | Songkhla | 18/2/2022 | 65 | 14.54 | BA.2 |
| COVPSU-01759 | male | Songkhla | 18/2/2022 | 49 | 13.76 | BA.1.1 |
| COVPSU-01760 | female | Songkhla | 18/2/2022 | 31 | 15.44 | BA.2 |
| COVPSU-01761 | female | Songkhla | 22/2/2022 | 28 | 15.24 | BA.1.1 |
| COVPSU-01762 | female | Songkhla | 22/2/2022 | 10 | 13.19 | BA.2 |
| COVPSU-01763 | female | Yala | 21/2/2022 | 58 | 20.85 | BA.2 |
| COVPSU-01764 | female | Yala | 21/2/2022 | 64 | 22.86 | BA.2 |
| COVPSU-01765 | female | Yala | 21/2/2022 | 44 | 19.74 | BA.2 |
| COVPSU-01766 | female | Yala | 19/2/2022 | 37 | 20.05 | BA.2 |
| COVPSU-01767 | female | Yala | 19/2/2022 | 32 | 18.11 | BA.2 |
| COVPSU-01768 | male | Songkhla | 19/2/2022 | 72 | 15.02 | BA.1.1 |
| COVPSU-01769 | male | Songkhla | 19/2/2022 | 32 | 17.02 | BA.1.1 |
| COVPSU-01770 | male | Songkhla | 19/2/2022 | 87 | 15.93 | BA.2 |
| COVPSU-01771 | male | Songkhla | 19/2/2022 | 72 | 11.09 | BA.2 |
| COVPSU-01772 | female | Songkhla | 19/2/2022 | 42 | 14.24 | BA.2 |
| COVPSU-01773 | female | Songkhla | 19/2/2022 | 30 | 17.5 | BA.2 |
| COVPSU-01774 | female | Songkhla | 19/2/2022 | 50 | 15.29 | BA.2 |
| COVPSU-01776 | female | Songkhla | 20/2/2022 | 44 | 13.88 | BA.1.1 |
| COVPSU-01777 | male | Songkhla | 20/2/2022 | 24 | 13.44 | BA.1.1 |
| COVPSU-01778 | male | Songkhla | 20/2/2022 | 19 | 16.89 | BA.1.1 |
| COVPSU-01779 | female | Songkhla | 20/2/2022 | 15 | 14.26 | BA.2 |
| COVPSU-01780 | male | Songkhla | 20/2/2022 | 59 | 15.49 | BA.2 |
| COVPSU-01781 | female | Songkhla | 20/2/2022 | 43 | 14.6 | BA.2 |
| COVPSU-01782 | female | Songkhla | 20/2/2022 | 32 | 18.48 | BA.1 |
| COVPSU-01783 | female | Yala | 21/2/2022 | 39 | 15.87 | BA.2 |
| COVPSU-01784 | female | Yala | 21/2/2022 | 36 | 12.42 | BA.2 |
| COVPSU-01785 | male | Songkhla | 23/2/2022 | 26 | 13.13 | BA.1.1 |
| COVPSU-01786 | female | Songkhla | 23/2/2022 | 32 | 12.01 | BA.2 |
| COVPSU-01787 | female | Songkhla | 21/2/2022 | 57 | NA | BA.1.1 |
| COVPSU-01788 | male | Songkhla | 19/2/2022 | 58 | NA | BA.2 |
| COVPSU-01789 | male | Songkhla | 19/2/2022 | 45 | NA | BA.1.1 |
| COVPSU-01790 | male | Songkhla | 19/2/2022 | 47 | NA | BA.1.1 |
| COVPSU-01791 | male | Songkhla | 19/2/2022 | 27 | NA | BA.1.1 |
| COVPSU-01792 | male | Songkhla | 19/2/2022 | 40 | NA | BA.2 |
| COVPSU-01793 | male | Songkhla | 19/2/2022 | 58 | NA | BA.2 |
| COVPSU-01794 | female | Songkhla | 21/2/2022 | NA | NA | BA.2 |
| COVPSU-01795 | female | Songkhla | 21/2/2022 | NA | NA | BA.1.1 |
| COVPSU-01796 | female | Songkhla | 21/2/2022 | NA | NA | BA.2 |
| COVPSU-01797 | female | Songkhla | 23/2/2022 | NA | NA | BA.2 |
| COVPSU-01798 | female | Songkhla | 21/2/2022 | NA | NA | BA.2 |
| COVPSU-01799 | male | Songkhla | 21/2/2022 | NA | NA | BA.2 |
| COVPSU-01800 | female | Songkhla | 23/2/2022 | NA | NA | BA.1.1 |
| COVPSU-01801 | female | Songkhla | 21/2/2022 | NA | NA | BA.1.1 |
| COVPSU-01802 | female | Songkhla | 22/2/2022 | NA | NA | BA.1.1 |
| COVPSU-01803 | female | Songkhla | 22/2/2022 | NA | NA | BA.2 |
| COVPSU-01804 | female | Songkhla | 21/2/2022 | NA | NA | BA.1.1 |
| COVPSU-01805 | female | Yala | 24/2/2022 | 25 | 19.19 | BA.2 |
| COVPSU-01806 | female | Yala | 21/2/2022 | 35 | 15.75 | BA.1.1 |
| COVPSU-01807 | female | Songkhla | 26/2/2022 | 1 | NA | BA.2 |
| COVPSU-01808 | female | Pattani | 22/2/2022 | 2 | NA | BA.2 |
| COVPSU-01812 | male | Narathiwat | 21/2/2022 | 52 | NA | BA.2 |
| COVPSU-01814 | male | Narathiwat | 18/2/2022 | 26 | NA | BA.2 |
| COVPSU-01816 | female | Yala | 23/2/2022 | 43 | 13.17 | BA.2 |
| COVPSU-01817 | female | Yala | 21/2/2022 | 46 | 20.16 | BA.1.1 |
| COVPSU-01818 | female | Yala | 22/2/2022 | NA | NA | BA.1.1 |
| COVPSU-01819 | male | Yala | 22/2/2022 | NA | NA | BA.2 |
| COVPSU-01820 | male | Yala | 26/2/2022 | NA | NA | BA.2 |
| COVPSU-01821 | female | Yala | 27/2/2022 | NA | NA | BA.2 |
| COVPSU-01822 | female | Songkhla | 28/2/2022 | 36 | NA | BA.1.1 |
| COVPSU-01831 | female | Phatthalung | 15/2/2022 | 23 | NA | BA.2 |
| COVPSU-01832 | female | Phatthalung | 15/2/2022 | 54 | NA | BA.1.1 |
| COVPSU-01839 | female | Yala | 21/2/2022 | 88 | 19.16 | BA.1.1 |
| COVPSU-01840 | female | Yala | 20/2/2022 | 34 | 15.87 | BA.1.1 |
| COVPSU-01841 | female | Yala | 20/2/2022 | 22 | 21.95 | BA.2 |
| COVPSU-01842 | male | Yala | 21/2/2022 | 30 | 19.12 | BA.1 |
| COVPSU-01843 | male | Yala | 21/2/2022 | 33 | 16.04 | BA.2 |
| COVPSU-01844 | female | Yala | 21/2/2022 | 33 | 21.85 | BA.2 |
| COVPSU-01845 | male | Yala | 21/2/2022 | 48 | 16.93 | BA.2 |
| COVPSU-01846 | male | Yala | 22/2/2022 | 8 | 17.56 | BA.2 |
| COVPSU-01847 | female | Yala | 22/2/2022 | 36 | 12.24 | BA.1 |
| COVPSU-01848 | male | Yala | 22/2/2022 | 25 | 22.57 | BA.1.1 |
| COVPSU-01849 | male | Yala | 22/2/2022 | 37 | 16.42 | BA.2 |
| COVPSU-01850 | male | Yala | 22/2/2022 | 27 | 21.18 | BA.2 |
| COVPSU-01851 | female | Yala | 22/2/2022 | 22 | 17.99 | BA.1.1 |
| COVPSU-01852 | female | Yala | 22/2/2022 | 66 | 18.86 | BA.2 |
| COVPSU-01853 | female | Yala | 26/2/2022 | 38 | 14.95 | BA.2 |
| COVPSU-01854 | male | Yala | 22/2/2022 | 26 | 23.49 | BA.2 |
| COVPSU-01855 | male | Yala | 24/2/2022 | 36 | 13.41 | BA.1.1 |
| COVPSU-01856 | female | Yala | 24/2/2022 | 27 | 14.3 | BA.2 |
| COVPSU-01857 | female | Yala | 26/2/2022 | 23 | 20.22 | BA.2 |
| COVPSU-01858 | female | Yala | 26/2/2022 | 23 | 17.36 | BA.1 |
| COVPSU-01859 | male | Yala | 26/2/2022 | 71 | 17.63 | BA.2 |
| COVPSU-01860 | female | Yala | 24/2/2022 | 74 | 15.3 | BA.1.1 |
| COVPSU-01861 | female | Narathiwat | 23/2/2022 | 47 | 16.87 | BA.2 |
| COVPSU-01862 | male | Narathiwat | 22/2/2022 | 59 | 15.19 | BA.2 |
| COVPSU-01863 | female | Narathiwat | 22/2/2022 | 90 | 15.36 | BA.2 |
| COVPSU-01864 | female | Narathiwat | 22/2/2022 | 96 | 16.22 | BA.2 |
| COVPSU-01865 | male | Narathiwat | 24/2/2022 | 63 | 19.81 | BA.2 |
| COVPSU-01866 | female | Narathiwat | 26/2/2022 | 43 | 11.86 | BA.1 |
| COVPSU-01867 | female | Narathiwat | 27/2/2022 | 57 | 17.69 | BA.2 |
| COVPSU-01868 | male | Narathiwat | 13/2/2022 | 69 | 15.86 | BA.2 |
| COVPSU-01869 | female | Narathiwat | 25/2/2022 | 33 | NA | BA.2 |
| COVPSU-01870 | male | Narathiwat | 25/2/2022 | 30 | 18.53 | BA.2 |
| COVPSU-01871 | female | Songkhla | 2/3/2022 | 30 | NA | BA.2 |
| COVPSU-01872 | male | Yala | 28/2/2022 | 28 | 20.71 | BA.1.1 |
| COVPSU-01873 | female | Yala | 26/2/2022 | 44 | 18.32 | BA.2 |
| COVPSU-01874 | female | Yala | 26/2/2022 | 56 | 19.07 | BA.2 |
| COVPSU-01887 | female | Pattani | 13/1/2022 | 15 | 22.64 | BA.1.17 |
| COVPSU-01888 | female | Pattani | 12/1/2022 | 28 | 21.26 | BA.1.1 |
| COVPSU-01890 | male | Pattani | 12/1/2022 | 23 | 18.57 | BA.1.1 |
| COVPSU-01892 | female | Songkhla | 11/1/2022 | NA | 25.2 | AY.85 |
| COVPSU-01893 | female | Songkhla | 11/1/2022 | NA | 14.61 | BA.1.18 |
| COVPSU-01894 | female | Songkhla | 11/1/2022 | NA | 20.04 | BA.1.18 |
| COVPSU-01895 | male | Songkhla | 11/1/2022 | NA | 28.22 | BA.1.1 |
| COVPSU-01896 | male | Songkhla | 10/1/2022 | NA | 19.35 | BA.1.1 |
| COVPSU-01897 | male | Songkhla | 10/1/2022 | NA | 16.41 | BA.1.1 |
| COVPSU-01899 | female | Yala | 10/1/2022 | NA | NA | AY.85 |
| COVPSU-01900 | female | Songkhla | 14/1/2022 | 19 | 15.9 | BA.1.17 |
| COVPSU-01901 | female | Songkhla | 14/1/2022 | 31 | 14.42 | BA.1.17.2 |
| COVPSU-01902 | male | Songkhla | 14/1/2022 | 27 | 13.36 | BA.1.1 |
| COVPSU-01903 | male | Songkhla | 14/1/2022 | 38 | 13.69 | AY.85 |
| COVPSU-01904 | female | Songkhla | 11/1/2022 | NA | 13.6 | AY.85 |
| COVPSU-01905 | female | Songkhla | 11/1/2022 | NA | 16.3 | BA.1.17 |
| COVPSU-01906 | female | Songkhla | 11/1/2022 | NA | 19.57 | BA.1.17 |
| COVPSU-01907 | female | Songkhla | 11/1/2022 | NA | 15.73 | BA.1.17 |
| COVPSU-01908 | male | Songkhla | 11/1/2022 | NA | 15.65 | BA.1.1 |
| COVPSU-01909 | male | Songkhla | 11/1/2022 | NA | 15.08 | BA.1.1 |
| COVPSU-01910 | female | Songkhla | 11/1/2022 | NA | 18 | AY.85 |
| COVPSU-01911 | male | Songkhla | 11/1/2022 | NA | 15.69 | BA.1.1 |
| COVPSU-01912 | male | Songkhla | 11/1/2022 | NA | 15.14 | BA.1.1 |
| COVPSU-01913 | male | Songkhla | 11/1/2022 | NA | 15.71 | BA.1.17 |
| COVPSU-01914 | male | Songkhla | 12/1/2022 | NA | 12.31 | AY.85 |
| COVPSU-01915 | male | Songkhla | 12/1/2022 | NA | 19.89 | BA.1.17 |
| COVPSU-01916 | female | Songkhla | 12/1/2022 | NA | 19.16 | BA.1.1 |
| COVPSU-01917 | female | Songkhla | 12/1/2022 | NA | 21.42 | BA.1.1 |
| COVPSU-01918 | female | Songkhla | 14/1/2022 | 33 | 21.75 | BA.2.10 |
| COVPSU-01919 | male | Songkhla | 8/1/2022 | 27 | 13.98 | BA.1.17 |
| COVPSU-01920 | male | Phatthalung | 8/1/2022 | 25 | NA | AY.46.6 |
| COVPSU-01943 | male | Phatthalung | 13/1/2022 | 80 | NA | AY.85 |
| COVPSU-01944 | female | Phatthalung | 13/1/2022 | 44 | NA | AY.85 |
| COVPSU-01945 | male | Phatthalung | 14/1/2022 | 15 | NA | BA.1.1 |
| COVPSU-01946 | male | Phatthalung | 14/1/2022 | 80 | NA | AY.85 |
| COVPSU-01947 | male | Phatthalung | 14/1/2022 | 36 | NA | AY.85 |
| COVPSU-01949 | female | Songkhla | 13/1/2022 | NA | 15.09 | BA.1.1 |
| COVPSU-01950 | female | Songkhla | 13/1/2022 | NA | 17.65 | AY.85 |
| COVPSU-01951 | male | Songkhla | 16/1/2022 | NA | 27.25 | BA.1 |
| COVPSU-01952 | female | Songkhla | 16/1/2022 | NA | 17.35 | BA.1 |
| COVPSU-01953 | female | Songkhla | 16/1/2022 | NA | 18.29 | BA.1 |
| COVPSU-01954 | female | Songkhla | 16/1/2022 | NA | 26.77 | BA.1 |
| COVPSU-01955 | male | Songkhla | 16/1/2022 | NA | 15.19 | BA.1 |
| COVPSU-01956 | female | Songkhla | 16/1/2022 | NA | 18.1 | BA.1.17 |
| COVPSU-01957 | female | Songkhla | 16/1/2022 | NA | 14.18 | BA.1.1 |
| COVPSU-01958 | female | Songkhla | 16/1/2022 | NA | 19.42 | BA.1.1 |
| COVPSU-01959 | female | Songkhla | 14/1/2022 | 87 | 17.32 | BA.1.1 |
| COVPSU-01960 | female | Songkhla | 14/1/2022 | 5 | 23.28 | BA.1 |
| COVPSU-01961 | female | Songkhla | 14/1/2022 | 2 | 17.97 | BA.1 |
| COVPSU-01962 | female | Songkhla | 14/1/2022 | 29 | 13.8 | BA.1 |
| COVPSU-01963 | female | Songkhla | 14/1/2022 | 26 | 20.29 | BA.1 |
| COVPSU-01964 | male | Songkhla | 14/1/2022 | 31 | 17.22 | BA.1 |
| COVPSU-01965 | male | Songkhla | 14/1/2022 | 1 | 22.78 | BA.1.17 |
| COVPSU-01966 | female | Songkhla | 14/1/2022 | 30 | 18.71 | BA.1.1 |
| COVPSU-01967 | male | Songkhla | 14/1/2022 | 48 | 19.03 | BA.1 |
| COVPSU-01968 | female | Songkhla | 15/1/2022 | NA | 12.08 | BA.2 |
| COVPSU-01969 | female | Songkhla | 15/1/2022 | NA | 12.76 | BA.1.1 |
| COVPSU-01970 | male | Songkhla | 16/1/2022 | NA | 15.03 | BA.1.1 |
| COVPSU-01971 | female | Songkhla | 16/1/2022 | NA | 17.36 | BA.1 |
| COVPSU-01972 | male | Yala | 10/1/2022 | NA | NA | BA.1.1 |
| COVPSU-01973 | male | Yala | 10/1/2022 | NA | NA | BA.1.1 |
| COVPSU-01974 | female | Yala | 10/1/2022 | NA | NA | BA.1.1.1 |
| COVPSU-01975 | female | Yala | 10/1/2022 | NA | NA | BA.1.1.1 |
| COVPSU-01976 | female | Yala | 10/1/2022 | NA | NA | AY.85 |
| COVPSU-01977 | female | Yala | 10/1/2022 | NA | NA | BA.1.1.1 |
| COVPSU-01978 | female | Yala | 10/1/2022 | NA | NA | BA.1.1.18 |
| COVPSU-01979 | male | Yala | 10/1/2022 | NA | NA | AY.85 |
| COVPSU-01980 | male | Yala | 10/1/2022 | NA | NA | BA.1.1 |
| COVPSU-01981 | female | Yala | 10/1/2022 | NA | NA | BA.1.1 |
| COVPSU-01982 | male | Yala | 10/1/2022 | NA | NA | BA.1.1 |
| COVPSU-01983 | female | Yala | 10/1/2022 | NA | NA | BA.1.1 |
| COVPSU-01984 | male | Yala | 10/1/2022 | 8 | NA | AY.85 |
| COVPSU-01985 | male | Songkhla | 16/1/2022 | NA | 14.25 | AY.85 |
| COVPSU-01986 | female | Songkhla | 17/1/2022 | NA | 13.02 | BA.1 |
| COVPSU-01987 | male | Songkhla | 17/1/2022 | 4 | 19.31 | BA.1.1 |
| COVPSU-01988 | female | Songkhla | 17/1/2022 | 13 | 21.9 | BA.1.1 |
| COVPSU-01989 | female | Songkhla | 17/1/2022 | 37 | 20 | BA.1.1 |
| COVPSU-01990 | female | Songkhla | 6/1/2022 | 25 | 21.99 | BA.1.17 |
| COVPSU-01991 | male | Songkhla | 6/1/2022 | 49 | 17.46 | AY.85 |
| COVPSU-01992 | female | Songkhla | 17/1/2022 | NA | 20.38 | BA.1.1 |
| COVPSU-01993 | female | Songkhla | 17/1/2022 | NA | 16.54 | BA.1 |
| COVPSU-01994 | female | Songkhla | 17/1/2022 | NA | 15.24 | BA.1 |
| COVPSU-01995 | female | Songkhla | 17/1/2022 | NA | 17.78 | BA.1 |
| COVPSU-01996 | male | Songkhla | 17/1/2022 | NA | 21.21 | BA.1 |
| COVPSU-01997 | male | Songkhla | 17/1/2022 | NA | 15.31 | BA.1 |
| COVPSU-01998 | male | Songkhla | 17/1/2022 | 57 | 15 | BA.1.1 |
| COVPSU-01999 | male | Songkhla | 17/1/2022 | 39 | 16.2 | BA.1.1 |
| COVPSU-02000 | female | Songkhla | 17/1/2022 | 32 | 15.23 | BA.1.17 |
| COVPSU-02001 | male | Songkhla | 18/1/2022 | 27 | 17.28 | BA.1.1 |
| COVPSU-02002 | female | Songkhla | 18/1/2022 | 40 | 14.09 | BA.1.1 |
| COVPSU-02003 | female | Songkhla | 19/1/2022 | NA | 20.62 | BA.1.17 |
| COVPSU-02004 | male | Songkhla | 19/1/2022 | NA | 13.97 | BA.1.1 |
| COVPSU-02005 | male | Songkhla | 19/1/2022 | NA | 13.09 | AY.85 |
| COVPSU-02007 | male | Songkhla | 5/1/2022 | 32 | 15.09 | BA.1.1 |
| COVPSU-02008 | male | Songkhla | 19/1/2022 | NA | 16.27 | BA.1.17 |
| COVPSU-02009 | male | Songkhla | 17/1/2022 | NA | 15.3 | BA.1.17 |
| COVPSU-02010 | male | Narathiwat | 17/1/2022 | 29 | NA | BA.1.17 |
| COVPSU-02011 | male | Narathiwat | 17/1/2022 | 39 | NA | BA.1.17 |
| COVPSU-02012 | female | Narathiwat | 17/1/2022 | 26 | NA | BA.1.17 |
| COVPSU-02014 | female | Songkhla | 20/1/2022 | NA | 23.88 | BA.1.1 |
| COVPSU-02015 | female | Songkhla | 20/1/2022 | NA | 23.98 | BA.1.1 |
| COVPSU-02016 | male | Songkhla | 20/1/2022 | NA | 14.36 | BA.1.1 |
| COVPSU-02017 | female | Songkhla | 20/1/2022 | NA | 24.72 | BA.1 |
| COVPSU-02018 | male | Songkhla | 20/1/2022 | NA | 19.86 | BA.1 |
| COVPSU-02019 | female | Songkhla | 20/1/2022 | NA | 21.31 | BA.1 |
| COVPSU-02020 | male | Songkhla | 20/1/2022 | NA | 17.84 | BA.1 |
| COVPSU-02021 | female | Songkhla | 20/1/2022 | NA | 18.48 | BA.1 |
| COVPSU-02022 | female | Songkhla | 20/1/2022 | NA | 22.74 | BA.1 |
| COVPSU-02023 | female | Songkhla | 20/1/2022 | NA | 20.27 | BA.1.1 |
| COVPSU-02024 | male | Songkhla | 20/1/2022 | NA | 19 | BA.1 |
| COVPSU-02025 | male | Yala | 19/1/2022 | 45 | 21.57 | AY.85 |
| COVPSU-02026 | male | Songkhla | 13/1/2022 | 38 | 16.59 | BA.1.17 |
| COVPSU-02027 | male | Songkhla | 13/1/2022 | 65 | 15.17 | BA.1.17 |
| COVPSU-02028 | male | Songkhla | 13/1/2022 | 54 | 16.26 | BA.1.1 |
| COVPSU-02029 | female | Songkhla | 13/1/2022 | 6 | 17.64 | BA.1.17 |
| COVPSU-02030 | male | Songkhla | 16/1/2022 | 31 | 13.49 | BA.2 |
| COVPSU-02031 | female | Songkhla | 15/1/2022 | 63 | 16.49 | BA.1.1 |
| COVPSU-02032 | female | Songkhla | 15/1/2022 | 23 | 16.05 | BA.1.1 |
| COVPSU-02033 | female | Songkhla | 16/1/2022 | 19 | 15.7 | BA.1.17 |
| COVPSU-02034 | female | Songkhla | 16/1/2022 | 35 | 12.52 | AY.85 |
| COVPSU-02035 | male | Songkhla | 17/1/2022 | 24 | 18.47 | BA.2 |
| COVPSU-02036 | female | Songkhla | 18/1/2022 | 52 | 15.52 | BA.1.17 |
| COVPSU-02037 | male | Songkhla | 19/1/2022 | 36 | 21.02 | BA.1 |
| COVPSU-02039 | male | Yala | 18/1/2022 | 17 | 23.58 | BA.1.1 |
| COVPSU-02040 | male | Songkhla | 21/1/2022 | NA | 19.15 | BA.1.1 |
| COVPSU-02041 | female | Songkhla | 21/1/2022 | NA | 16.86 | BA.1.17 |
| COVPSU-02042 | male | Songkhla | 21/1/2022 | NA | 15.55 | BA.1.1 |
| COVPSU-02043 | female | Songkhla | 21/1/2022 | NA | 15.17 | BA.1 |
| COVPSU-02044 | male | Songkhla | 21/1/2022 | NA | 20.66 | BA.1 |
| COVPSU-02045 | male | Songkhla | 17/1/2022 | NA | 15.1 | BA.1.17 |
| COVPSU-02046 | female | Songkhla | 17/1/2022 | NA | 20.87 | BA.1.17 |
| COVPSU-02047 | female | Songkhla | 17/1/2022 | NA | 16.32 | AY.30 |
| COVPSU-02048 | female | Songkhla | 17/1/2022 | NA | 17.38 | AY.30 |
| COVPSU-02049 | male | Songkhla | 17/1/2022 | NA | 17.25 | AY.30 |
| COVPSU-02050 | female | Songkhla | 17/1/2022 | NA | 18.73 | BA.1.17 |
| COVPSU-02051 | male | Songkhla | 18/1/2022 | NA | 18.03 | BA.1.17 |
| COVPSU-02052 | female | Songkhla | 18/1/2022 | NA | 15.5 | AY.85 |
| COVPSU-02053 | male | Songkhla | 18/1/2022 | NA | 18.98 | B.1.617.2 |
| COVPSU-02054 | female | Songkhla | 18/1/2022 | NA | 13.92 | AY.85 |
| COVPSU-02055 | female | Songkhla | 18/1/2022 | NA | 14.77 | AY.85 |
| COVPSU-02056 | female | Songkhla | 18/1/2022 | NA | 16.12 | BA.1.1 |
| COVPSU-02057 | female | Songkhla | 19/1/2022 | 30 | 14.83 | BA.1.1 |
| COVPSU-02058 | female | Songkhla | 20/1/2022 | 10 | 14.44 | BA.1.15.1 |
| COVPSU-02059 | female | Songkhla | 21/1/2022 | 31 | 14.67 | BA.1.17 |
| COVPSU-02060 | female | Songkhla | 21/1/2022 | 32 | 15.1 | BA.1.17 |
| COVPSU-02061 | female | Songkhla | 21/1/2022 | NA | 15.18 | BA.1.15 |
| COVPSU-02062 | male | Phatthalung | 15/1/2022 | 16 | NA | BA.1.1 |
| COVPSU-02063 | female | Phatthalung | 15/1/2022 | 20 | NA | AY.85 |
| COVPSU-02064 | female | Phatthalung | 15/1/2022 | 45 | NA | AY.85 |
| COVPSU-02065 | female | Phatthalung | 15/1/2022 | 26 | NA | AY.46.6 |
| COVPSU-02066 | female | Phatthalung | 15/1/2022 | 56 | NA | AY.85 |
| COVPSU-02067 | male | Phatthalung | 16/1/2022 | 58 | NA | AY.85 |
| COVPSU-02068 | female | Phatthalung | 16/1/2022 | 11 | NA | AY.85 |
| COVPSU-02069 | male | Phatthalung | 16/1/2022 | 17 | NA | BA.1.1 |
| COVPSU-02070 | male | Phatthalung | 17/1/2022 | 18 | 17.2 | BA.1.17 |
| COVPSU-02071 | female | Phatthalung | 17/1/2022 | 33 | 14.3 | AY.85 |
| COVPSU-02072 | male | Phatthalung | 17/1/2022 | 37 | 12.1 | AY.85 |
| COVPSU-02074 | female | Phatthalung | 18/1/2022 | 27 | NA | BA.1.17 |
| COVPSU-02075 | male | Phatthalung | 18/1/2022 | 68 | NA | AY.85 |
| COVPSU-02077 | male | Phatthalung | 19/1/2022 | 29 | NA | BA.1.17.2 |
| COVPSU-02078 | female | Phatthalung | 19/1/2022 | 77 | NA | AY.85 |
| COVPSU-02080 | female | Phatthalung | 19/1/2022 | 62 | NA | BA.1.1 |
| COVPSU-02081 | male | Phatthalung | 20/1/2022 | 55 | NA | BA.1.1 |
| COVPSU-02082 | male | Phatthalung | 20/1/2022 | 53 | NA | BA.1.1 |
| COVPSU-02083 | female | Phatthalung | 20/1/2022 | 36 | NA | BA.1.17.2 |
| COVPSU-02084 | female | Phatthalung | 20/1/2022 | 35 | NA | AY.85 |
| COVPSU-02086 | male | Phatthalung | 21/1/2022 | 40 | NA | AY.85 |
| COVPSU-02087 | female | Phatthalung | 21/1/2022 | 55 | NA | BA.1.1 |
| COVPSU-02088 | female | Phatthalung | 21/1/2022 | 36 | NA | BA.1.1 |
| COVPSU-02089 | male | Songkhla | 23/1/2022 | NA | 19.61 | BA.1 |
| COVPSU-02090 | male | Songkhla | 23/1/2022 | NA | 17.22 | BA.1.17 |
| COVPSU-02091 | female | Songkhla | 22/1/2022 | 32 | 18.23 | BA.2.3 |
| COVPSU-02105 | female | Yala | 19/1/2022 | 22 | 22.72 | AY.85 |
| COVPSU-02106 | male | Songkhla | 24/1/2022 | 60 | 15.58 | BA.1.17 |
| COVPSU-02107 | female | Songkhla | 26/1/2022 | NA | 17.97 | BA.1.1 |
| COVPSU-02108 | male | Narathiwat | 18/1/2022 | 58 | 20.86 | BA.1.17 |
| COVPSU-02109 | female | Narathiwat | 20/1/2022 | 1 | 22.77 | BA.1.17 |
| COVPSU-02110 | male | Narathiwat | 22/1/2022 | 26 | 23.96 | BA.1.17 |
| COVPSU-02111 | female | Narathiwat | 22/1/2022 | 27 | 20.09 | BA.1.17 |
| COVPSU-02112 | male | Narathiwat | 23/1/2022 | 70 | 17.3 | BA.1.1 |
| COVPSU-02113 | female | Narathiwat | 23/1/2022 | 41 | 18.76 | BA.2.9 |
| COVPSU-02114 | male | Narathiwat | 23/1/2022 | 42 | 19.3 | BA.1.1 |
| COVPSU-02115 | female | Songkhla | 14/1/2022 | NA | 13.4 | AY.85 |
| COVPSU-02116 | female | Songkhla | 23/1/2022 | NA | 15.95 | BA.1.17 |
| COVPSU-02117 | female | Songkhla | 24/1/2022 | NA | 17.94 | BA.1.17 |
| COVPSU-02118 | female | Songkhla | 24/1/2022 | NA | 27.08 | BA.1 |
| COVPSU-02119 | NA | Songkhla | 24/1/2022 | NA | 18.29 | BA.1.1 |
| COVPSU-02120 | female | Yala | 19/1/2022 | 32 | 23.84 | AY.85 |
| COVPSU-02121 | female | Yala | 23/1/2022 | 34 | 15.29 | AY.85 |
| COVPSU-02122 | male | Songkhla | 20/1/2022 | 48 | 14.8 | BA.1.17.2 |
| COVPSU-02123 | female | Songkhla | 22/1/2022 | 58 | 21.12 | BA.1.17 |
| COVPSU-02124 | female | Songkhla | 22/1/2022 | 32 | 18.32 | BA.1 |
| COVPSU-02125 | female | Songkhla | 24/1/2022 | 25 | 17.3 | BA.1 |
| COVPSU-02126 | female | Songkhla | 25/1/2022 | 23 | 19.13 | BA.1.1 |
| COVPSU-02127 | female | Songkhla | 25/1/2022 | 58 | 15.81 | BA.1.1.15 |
| COVPSU-02128 | female | Songkhla | 22/1/2022 | 58 | - | AY.85 |
| COVPSU-02129 | male | Songkhla | 25/1/2022 | 10 | 15.93 | BA.1 |
| COVPSU-02130 | female | Songkhla | 25/1/2022 | 7 | 13.47 | BA.2.3 |
| COVPSU-02131 | female | Songkhla | 25/1/2022 | 8 | 16.48 | BA.2.3 |
| COVPSU-02132 | male | Songkhla | 26/1/2022 | NA | 15.27 | BA.1.1 |
| COVPSU-02133 | male | Songkhla | 26/1/2022 | NA | 11.8 | BA.1.15.1 |
| COVPSU-02134 | female | Songkhla | 25/1/2022 | 36 | 26.89 | BA.1.1 |
| COVPSU-02135 | male | Songkhla | 26/1/2022 | 73 | 20.82 | BA.1 |
| COVPSU-02136 | female | Songkhla | 26/1/2022 | 15 | 22.46 | BA.1 |
| COVPSU-02137 | male | Songkhla | 26/1/2022 | 41 | 22.17 | BA.1 |
| COVPSU-02138 | female | Songkhla | 25/1/2022 | 50 | 17.7 | BA.1.1 |
| COVPSU-02144 | female | Songkhla | 26/1/2022 | 85 | 22.74 | BA.1 |
| COVPSU-02145 | male | Songkhla | 26/1/2022 | 91 | 25.78 | BA.1.1 |
| COVPSU-02146 | female | Yala | 24/1/2022 | NA | 15.41 | AY.85 |
| COVPSU-02147 | female | Yala | 24/1/2022 | NA | 17.21 | BA.1.17 |
| COVPSU-02148 | female | Yala | 24/1/2022 | NA | 19.37 | BA.1.1 |
| COVPSU-02149 | female | Yala | 25/1/2022 | NA | 24.21 | BA.1.1 |
| COVPSU-02150 | male | Pattani | 22/1/2022 | NA | 9.62 | AY.85 |
| COVPSU-02151 | male | Pattani | 22/1/2022 | NA | 15.6 | AY.85 |
| COVPSU-02152 | female | Phatthalung | 22/1/2022 | 30 | NA | AY.85 |
| COVPSU-02153 | male | Phatthalung | 22/1/2022 | 45 | NA | BA.1.17.2 |
| COVPSU-02154 | female | Phatthalung | 22/1/2022 | 38 | NA | BA.1.1 |
| COVPSU-02155 | female | Phatthalung | 22/1/2022 | 85 | NA | BA.1.1 |
| COVPSU-02156 | female | Phatthalung | 23/1/2022 | 56 | NA | BA.1.1 |
| COVPSU-02157 | female | Phatthalung | 23/1/2022 | 25 | NA | AY.85 |
| COVPSU-02158 | female | Phatthalung | 23/1/2022 | 35 | NA | BA.1.1.15 |
| COVPSU-02159 | male | Phatthalung | 23/1/2022 | 44 | NA | AY.85 |
| COVPSU-02160 | male | Phatthalung | 23/1/2022 | 41 | NA | AY.85 |
| COVPSU-02161 | male | Phatthalung | 24/1/2022 | 71 | NA | BA.1.1.15 |
| COVPSU-02163 | male | Phatthalung | 24/1/2022 | 72 | NA | BA.1.17.2 |
| COVPSU-02164 | female | Phatthalung | 24/1/2022 | 11 | NA | AY.85 |
| COVPSU-02165 | female | Phatthalung | 24/1/2022 | 64 | NA | AY.85 |
| COVPSU-02167 | female | Phatthalung | 25/1/2022 | 26 | NA | BA.1.1 |
| COVPSU-02168 | female | Phatthalung | 25/1/2022 | 58 | NA | BA.1.17.2 |
| COVPSU-02169 | male | Phatthalung | 25/1/2022 | 54 | NA | BA.1.1 |
| COVPSU-02170 | female | Phatthalung | 26/1/2022 | 17 | NA | BA.1.17.2 |
| COVPSU-02171 | female | Phatthalung | 26/1/2022 | 52 | NA | AY.85 |
| COVPSU-02172 | female | Phatthalung | 26/1/2022 | 35 | NA | BA.1.1.15 |
| COVPSU-02173 | female | Phatthalung | 26/1/2022 | 79 | NA | BA.1.1.15 |
| COVPSU-02175 | male | Phatthalung | 27/1/2022 | 38 | NA | BA.1.17 |
| COVPSU-02177 | male | Phatthalung | 28/1/2022 | 19 | NA | BA.1.1.15 |
| COVPSU-02178 | female | Phatthalung | 28/1/2022 | 69 | NA | AY.85 |
| COVPSU-02179 | female | Songkhla | 23/1/2022 | 51 | 14.59 | BA.1.1 |
| COVPSU-02180 | female | Songkhla | 23/1/2022 | 34 | 21.94 | BA.1.1 |
| COVPSU-02181 | female | Songkhla | 28/1/2022 | NA | 13.95 | BA.2.5 |
| COVPSU-02182 | female | Songkhla | 29/1/2022 | NA | 16.23 | BA.2.5 |
| COVPSU-02183 | female | Narathiwat | 27/1/2022 | 28 | 18.98 | BA.2.10 |
| COVPSU-02184 | female | Yala | 24/1/2022 | 77 | 12.35 | BA.1.1 |
| COVPSU-02185 | male | Yala | 24/1/2022 | 82 | 15.7 | BA.1.1 |
| COVPSU-02186 | female | Yala | 25/1/2022 | 25 | 21.34 | BA.1.1 |
| COVPSU-02187 | female | Yala | 25/1/2022 | 12 | 14.45 | BA.1.1 |
| COVPSU-02188 | female | Yala | 28/1/2022 | 42 | 22.4 | BA.1.1 |
| COVPSU-02189 | female | Songkhla | 30/1/2022 | 10 | 12.17 | AY.85 |
| COVPSU-02190 | female | Songkhla | 30/1/2022 | 10 | 14.36 | AY.85 |
| COVPSU-02191 | female | Songkhla | 30/1/2022 | 40 | 15.22 | BA.1.1 |
| COVPSU-02192 | female | Songkhla | 1/2/2022 | 9 | 12.04 | BA.1.1 |
| COVPSU-02193 | female | Songkhla | 26/1/2022 | NA | 17.59 | AY.85 |
| COVPSU-02194 | male | Narathiwat | 24/1/2022 | 47 | NA | BA.2.9 |
| COVPSU-02195 | male | Narathiwat | 24/1/2022 | 1 | 20.7 | BA.2.10 |
| COVPSU-02196 | female | Yala | 29/1/2022 | NA | NA | BA.1 |
| COVPSU-02197 | female | Yala | 30/1/2022 | NA | NA | BA.2.10 |
| COVPSU-02198 | female | Yala | 31/1/2022 | NA | NA | BA.1.1 |
| COVPSU-02199 | male | Yala | 2/2/2022 | NA | NA | BA.2 |
| COVPSU-02200 | female | Songkhla | 26/1/2022 | NA | 17.93 | BA.1.1 |
| COVPSU-02201 | male | Pattani | 27/1/2022 | 60 | 17.47 | AY.85 |
| COVPSU-02202 | male | Pattani | 1/2/2022 | 31 | 16.96 | BA.1.1 |
| COVPSU-02203 | female | Songkhla | 3/2/2022 | 46 | 15.04 | BA.1.1 |
| COVPSU-02204 | male | Songkhla | 3/2/2022 | 34 | 13.32 | AY.85 |
| COVPSU-02205 | female | Songkhla | 4/2/2022 | 52 | 14.88 | BA.1.17 |
| COVPSU-02206 | male | Songkhla | 4/2/2022 | 32 | 15.6 | BA.1 |
| COVPSU-02207 | female | Songkhla | 4/2/2022 | 35 | 15.05 | BA.1 |
| COVPSU-02208 | female | Phatthalung | 29/1/2022 | 35 | NA | BA.1.1 |
| COVPSU-02209 | female | Phatthalung | 29/1/2022 | 27 | NA | BA.1.1 |
| COVPSU-02210 | male | Phatthalung | 29/1/2022 | 58 | NA | BA.1.1 |
| COVPSU-02211 | female | Phatthalung | 30/1/2022 | 59 | NA | BA.1.17 |
| COVPSU-02212 | female | Phatthalung | 30/1/2022 | 32 | NA | BA.1.1.15 |
| COVPSU-02213 | female | Phatthalung | 31/1/2022 | 40 | NA | BA.1.1 |
| COVPSU-02215 | male | Phatthalung | 1/2/2022 | 64 | NA | BA.1.1.15 |
| COVPSU-02217 | female | Phatthalung | 2/2/2022 | 62 | NA | BA.1.1 |
| COVPSU-02218 | female | Phatthalung | 2/2/2022 | 41 | NA | BA.1.1 |
| COVPSU-02219 | female | Phatthalung | 2/2/2022 | 71 | NA | BA.1.1 |
| COVPSU-02220 | female | Phatthalung | 3/2/2022 | 68 | NA | BA.1.1 |
| COVPSU-02221 | female | Phatthalung | 3/2/2022 | 18 | NA | BA.1.1.15 |
| COVPSU-02222 | male | Phatthalung | 3/2/2022 | 70 | NA | BA.1.1.15 |
| COVPSU-02226 | male | Songkhla | 5/2/2022 | 42 | 13.48 | AY.85 |
| COVPSU-02227 | female | Songkhla | 6/2/2022 | 25 | 14.15 | BA.1.1 |
| COVPSU-02228 | male | Songkhla | 6/2/2022 | NA | 13.01 | BA.2 |
| COVPSU-02229 | male | Narathiwat | 2/2/2022 | 26 | 19.73 | BA.1.1 |
| COVPSU-02230 | male | Narathiwat | 2/2/2022 | 52 | 22.72 | AY.79 |
| COVPSU-02231 | female | Narathiwat | 4/2/2022 | 36 | 27.67 | BA.2.10 |
| COVPSU-02232 | male | Narathiwat | 4/2/2022 | 32 | 29.19 | AY.79 |
| COVPSU-02233 | female | Yala | 31/1/2022 | 49 | 16.72 | BA.1.1 |
| COVPSU-02234 | female | Yala | 4/2/2022 | 23 | 16.29 | BA.1.1 |
| COVPSU-02235 | male | Yala | 22/1/2022 | 69 | 23.9 | AY.85 |
| COVPSU-02236 | male | Yala | 3/2/2022 | 57 | 21.23 | BA.1.16.1 |
| COVPSU-02237 | female | Songkhla | 7/2/2022 | NA | 16.55 | BA.1 |
| COVPSU-02238 | female | Songkhla | 7/2/2022 | NA | 14.09 | BA.1.1 |
| COVPSU-02244 | male | Songkhla | 7/2/2022 | 37 | 14.15 | BA.2.27 |
| COVPSU-02245 | female | Songkhla | 8/2/2022 | 44 | 16.7 | BA.2.3 |
| COVPSU-02246 | female | Songkhla | 6/2/2022 | 31 | 14.89 | BA.1.16.1 |
| COVPSU-02247 | male | Narathiwat | 4/2/2022 | 16 | 19.99 | BA.2.10 |
| COVPSU-02248 | male | Narathiwat | 7/2/2022 | 73 | NA | AY.85 |
| COVPSU-02249 | female | Narathiwat | 5/2/2022 | 67 | 24.19 | BA.2.10 |
| COVPSU-02250 | female | Narathiwat | 4/2/2022 | 36 | 22.05 | BA.2.10 |
| COVPSU-02251 | female | Songkhla | 10/2/2022 | NA | 18.58 | BA.1.1 |
| COVPSU-02252 | male | Songkhla | 10/2/2022 | NA | 14.91 | BA.1.1 |
| COVPSU-02253 | female | Songkhla | 10/2/2022 | NA | 17.44 | BA.2 |
| COVPSU-02254 | male | Songkhla | 10/2/2022 | NA | 15.16 | BA.1 |
| COVPSU-02255 | female | Songkhla | 10/2/2022 | NA | 23.03 | AY.85 |
| COVPSU-02256 | female | Songkhla | 10/2/2022 | NA | 13 | BA.1 |
| COVPSU-02257 | female | Songkhla | 10/2/2022 | NA | 16.12 | AY.85 |
| COVPSU-02258 | male | Songkhla | 10/2/2022 | NA | 27.11 | BA.1 |
| COVPSU-02259 | male | Songkhla | 10/2/2022 | NA | 34.45 | BA.1.17 |
| COVPSU-02260 | male | Songkhla | 10/2/2022 | NA | 23.52 | BA.2 |
| COVPSU-02261 | male | Songkhla | 10/2/2022 | NA | 19.12 | BA.1.1 |
| COVPSU-02262 | female | Songkhla | 4/2/2022 | NA | 14.11 | BA.2.3 |
| CSEQ-001 | NA | Songkhla | 1/7/2021 | NA | 16.43 | AY.30 |
| CSEQ-002 | NA | Songkhla | 24/4/2021 | NA | 13.73 | B.1.1.7 |
| CSEQ-003 | NA | Songkhla | 24/4/2021 | NA | 19.11 | B.1.1.7 |
| CSEQ-006 | NA | Songkhla | 30/6/2021 | NA | 14.8 | B.1.351 |
| CSEQ-007 | NA | Songkhla | 27/6/2021 | NA | 14.38 | B.1.351 |
| CSEQ-008 | NA | Songkhla | 28/6/2021 | NA | 31.62 | B.1.1.7 |
| CSEQ-011 | NA | Songkhla | 26/6/2021 | NA | 16.85 | B.1.351 |
| CSEQ-012 | NA | Songkhla | 29/6/2021 | NA | 18.29 | AY.30 |
| CSEQ-023 | NA | Songkhla | 7/7/2021 | NA | 13.73 | B.1.1.7 |
| CSEQ-025 | NA | Songkhla | 2/7/2021 | NA | 22.9 | B.1.1.7 |
| CSEQ-027 | NA | Songkhla | 2/7/2021 | NA | 22.38 | B.1.351 |
| CSEQ-034 | NA | Songkhla | 26/6/2021 | NA | 15.59 | AY.30 |
| CSEQ-035 | NA | Songkhla | 4/7/2021 | NA | 24.42 | B.1.1.7 |
| CSEQ-036 | NA | Songkhla | 5/7/2021 | NA | 28.07 | B.1.617.2 |
| CSEQ-043 | NA | Songkhla | 11/4/2021 | NA | 11.01 | B.1.1.7 |
| CSEQ-045 | NA | Songkhla | 5/4/2021 | NA | 11.79 | B.1.1.7 |
| CSEQ-061-KIT | NA | Songkhla | 25/5/2021 | NA | 25.41 | B.1.617.2 |
| CSEQ-062 | NA | Songkhla | 25/5/2021 | NA | 25.33 | B.1.1.7 |
| CSEQ-080 | NA | Songkhla | 9/6/2021 | NA | 13.7 | B.1.1.7 |
| CSEQ-083 | NA | Songkhla | 11/6/2021 | NA | 25.94 | B.1.1.7 |
| CSEQ-084 | NA | Songkhla | 11/6/2021 | NA | 23.8 | B.1.1.7 |
| CSEQ-087 | NA | Songkhla | 16/6/2021 | NA | 15.21 | B.1.1.7 |
| CSEQ-091 | NA | Songkhla | 18/6/2021 | NA | 17.17 | B.1.351 |
| CSEQ-092 | NA | Songkhla | 20/6/2021 | NA | 14.84 | B.1.351 |
| CSEQ-124 | NA | Songkhla | 1/7/2021 | NA | 13.49 | B.1.351 |
| CSEQ-127 | NA | Songkhla | 22/6/2021 | NA | 25.12 | AY.30 |
| CSEQ-151 | NA | Songkhla | 21/7/2021 | NA | 14.45 | B.1.1.7 |
| CSEQ-158 | NA | Songkhla | 31/7/2021 | NA | 15.12 | B.1.1.7 |
| CSEQ-159 | NA | Songkhla | 1/8/2021 | NA | 14.02 | AY.30 |
| CSEQ-173 | NA | Songkhla | 22/8/2021 | NA | 22.19 | AY.30 |
| CSEQ-180 | NA | Songkhla | 14/8/2021 | NA | 19.13 | B.1.617.2 |
| CSEQ-195 | NA | Songkhla | 9/8/2021 | NA | 12.94 | B.1.1.7 |
| CSEQ-196 | NA | Songkhla | 12/8/2021 | NA | 21.13 | AY.30 |
| CSEQ-219 | NA | Songkhla | 30/8/2021 | NA | 12.12 | AY.30 |
| CSEQ-220 | NA | Songkhla | 30/8/2021 | NA | 13.99 | B.1.1.7 |
| CSEQ-224 | NA | Songkhla | 8/8/2021 | NA | 22.98 | B.1.617.2 |
| CSEQ-226 | NA | Songkhla | 11/9/2021 | NA | 11.61 | B.1.1.7 |
| CSEQ-229-1 | NA | Songkhla | 11/9/2021 | NA | 14.09 | AY.30 |
| CSEQ-237 | NA | Songkhla | 27/9/2021 | NA | 10.63 | AY.30 |
| CSEQ-242 | NA | Songkhla | 3/10/2021 | NA | 12.21 | B.1.617.2 |
| CSEQ-245 | NA | Songkhla | 3/10/2021 | NA | 13.59 | B.1.617.2 |
| CSEQ-246 | NA | Songkhla | 3/10/2021 | NA | 15.38 | AY.30 |
| CSEQ-249 | NA | Songkhla | 4/10/2021 | NA | 16.12 | B.1.617.2 |
| CSEQ-252 | NA | Songkhla | 7/10/2021 | NA | 24.5 | B.1.617.2 |
| CSEQ-253 | NA | Songkhla | 7/10/2021 | NA | 16.77 | B.1.617.2 |
| CSEQ-254 | NA | Songkhla | 4/10/2021 | NA | 11.11 | B.1.617.2 |
| CSEQ-255 | NA | Songkhla | 4/10/2021 | NA | 13.56 | B.1.617.2 |
| CSEQ-256-1 | NA | Songkhla | 4/10/2021 | NA | 10.64 | B.1.617.2 |
| CSEQ014 | NA | Songkhla | 13/5/2021 | NA | 25.88 | B.1.1.7 |
| CSEQ015 | NA | Songkhla | 3/5/2021 | NA | 12.82 | B.1.1.7 |
| CSEQ017 | NA | Songkhla | 30/4/2021 | NA | 18.14 | B.1.1.7 |
| CSEQ021 | NA | Songkhla | 20/6/2021 | NA | 16.97 | B.1.1.7 |
| CSEQ024 | NA | Songkhla | 2/7/2021 | NA | 19.29 | B.1.1.7 |
| CSEQ028 | NA | Songkhla | 26/5/2021 | NA | 24.18 | B.1.1.7 |
| CSEQ030 | NA | Songkhla | 7/7/2021 | NA | 16.69 | B.1.351 |
| CSEQ032 | NA | Songkhla | 19/4/2021 | NA | 25.63 | B.1.1.7 |
| CSEQ037 | NA | Songkhla | 5/7/2021 | NA | 26.42 | B.1.1.7 |
| CSEQ095 | NA | Songkhla | 1/7/2021 | NA | 13.05 | B.1.1.7 |
| CSEQ110 | NA | Songkhla | 8/6/2021 | NA | 24.76 | B.1.1.7 |
| CSEQ111 | NA | Songkhla | 7/5/2021 | NA | 18.19 | B.1.1.7 |
| CSEQ113 | NA | Songkhla | 23/6/2021 | NA | 20.89 | B.1.1.7 |
| CSEQ114 | NA | Songkhla | 24/6/2021 | NA | 16.86 | AY.85 |
| CSEQ117 | NA | Songkhla | 1/7/2021 | NA | 17.05 | B.1.351 |
| CSEQ118 | NA | Songkhla | 26/6/2021 | NA | 34.92 | B.1.1.7 |
| CSEQ119 | NA | Songkhla | 1/7/2021 | NA | 29.59 | B.1.1.7 |
| CSEQ121 | NA | Songkhla | 26/6/2021 | NA | 16.93 | B.1.1.7 |
| CSEQ122 | NA | Songkhla | 26/6/2021 | NA | 23.432 | B.1.351 |
| CSEQ125 | NA | Songkhla | 15/6/2021 | NA | 30.82 | B.1.351 |
| CSEQ129 | NA | Songkhla | 1/7/2021 | NA | 23.22 | B.1.351 |
| CSEQ131 | NA | Songkhla | 26/6/2021 | NA | 21.63 | B.1.1.7 |
| CSEQ132 | NA | Songkhla | 1/7/2021 | NA | 22.64 | B.1.351 |
| CSEQ133 | NA | Songkhla | 25/6/2021 | NA | 13.5 | AY.85 |
| CSEQ135 | NA | Songkhla | 1/7/2021 | NA | 27.4 | B.1.351 |
| CSEQ136 | NA | Songkhla | 25/6/2021 | NA | 15.71 | B.1.1.7 |
| CSEQ141 | NA | Songkhla | 24/6/2021 | NA | 24.72 | B.1.351 |
| WGCV-04651 | male | Narathiwat | 9/10/2021 | 25 | NA | B.1.617.2 |
| WGCV-04652 | male | Songkhla | 11/10/2021 | 32 | NA | AY.30 |
| WGCV-04653 | female | Narathiwat | 12/10/2021 | 6 | NA | B.1.617.2 |
| WGCV-04654 | male | Songkhla | 14/10/2021 | 36 | NA | AY.30 |
| WGCV-04655 | male | Songkhla | 14/10/2021 | 5 | NA | AY.30 |
| WGCV-04659 | female | Narathiwat | 9/10/2021 | 48 | NA | B.1.617.2 |
| WGCV-04660 | male | Songkhla | 11/10/2021 | 20 | NA | AY.30 |
| WGCV-04661 | female | Narathiwat | 12/10/2021 | 40 | NA | B.1.617.2 |
| WGCV-04662 | female | Songkhla | 14/10/2021 | 35 | NA | AY.30 |
| WGCV-04663 | male | Songkhla | 14/10/2021 | 18 | NA | AY.30 |
| WGCV-04667 | female | Narathiwat | 9/10/2021 | 60 | NA | B.1.617.2 |
| WGCV-04668 | female | Songkhla | 11/10/2021 | 12 | NA | B.1.617.2 |
| WGCV-04669 | male | Narathiwat | 12/10/2021 | 23 | NA | AY.30 |
| WGCV-04670 | male | Songkhla | 14/10/2021 | 1 | NA | B.1.617.2 |
| WGCV-04671 | female | Songkhla | 15/10/2021 | 32 | NA | AY.30 |
| WGCV-04675 | female | Narathiwat | 9/10/2021 | 20 | NA | B.1.617.2 |
| WGCV-04676 | female | Songkhla | 11/10/2021 | 35 | NA | B.1.617.2 |
| WGCV-04677 | female | Narathiwat | 12/10/2021 | 68 | NA | B.1.617.2 |
| WGCV-04678 | male | Songkhla | 14/10/2021 | 26 | NA | AY.30 |
| WGCV-04679 | male | Songkhla | 15/10/2021 | 23 | NA | B.1.617.2 |
| WGCV-04683 | male | Narathiwat | 9/10/2021 | 18 | NA | B.1.617.2 |
| WGCV-04684 | female | Songkhla | 11/10/2021 | 10 | NA | B.1.617.2 |
| WGCV-04685 | female | Narathiwat | 12/10/2021 | 70 | NA | B.1.617.2 |
| WGCV-04686 | male | Songkhla | 14/10/2021 | 32 | NA | AY.30 |
| WGCV-04687 | male | Songkhla | 15/10/2021 | 20 | NA | B.1.617.2 |
| WGCV-04691 | female | Narathiwat | 9/10/2021 | 30 | NA | B.1.617.2 |
| WGCV-04692 | female | Songkhla | 11/10/2021 | 53 | NA | B.1.617.2 |
| WGCV-04693 | female | Narathiwat | 12/10/2021 | 36 | NA | B.1.617.2 |
| WGCV-04694 | male | Songkhla | 14/10/2021 | 22 | NA | B.1.617.2 |
| WGCV-04695 | male | Songkhla | 15/10/2021 | 26 | NA | AY.30 |
| WGCV-04699 | female | Narathiwat | 9/10/2021 | 19 | NA | B.1.617.2 |
| WGCV-04700 | male | Songkhla | 11/10/2021 | 26 | NA | AY.30 |
| WGCV-04701 | female | Narathiwat | 13/10/2021 | 52 | NA | B.1.617.2 |
| WGCV-04702 | female | Songkhla | 14/10/2021 | 3 | NA | AY.30 |
| WGCV-04703 | male | Songkhla | 15/10/2021 | 27 | NA | B.1.617.2 |
| WGCV-04707 | male | Narathiwat | 9/10/2021 | 69 | NA | B.1.617.2 |
| WGCV-04708 | male | Songkhla | 11/10/2021 | 35 | NA | AY.30 |
| WGCV-04709 | female | Narathiwat | 13/10/2021 | 32 | NA | B.1.617.2 |
| WGCV-04710 | female | Songkhla | 14/10/2021 | 33 | NA | AY.30 |
| WGCV-04711 | male | Songkhla | 15/10/2021 | 61 | NA | B.1.617.2 |
| WGCV-04715 | male | Narathiwat | 10/10/2021 | 80 | NA | B.1.617.2 |
| WGCV-04716 | male | Songkhla | 11/10/2021 | 68 | NA | B.1.617.2 |
| WGCV-04717 | male | Songkhla | 14/10/2021 | 48 | NA | AY.30 |
| WGCV-04718 | female | Songkhla | 14/10/2021 | 26 | NA | B.1.617.2 |
| WGCV-04719 | female | Pattani | 15/10/2021 | 16 | NA | B.1.617.2 |
| WGCV-04722 | female | Narathiwat | 9/10/2021 | 67 | NA | B.1.617.2 |
| WGCV-04723 | male | Narathiwat | 10/10/2021 | 35 | NA | B.1.617.2 |
| WGCV-04724 | male | Songkhla | 11/10/2021 | 17 | NA | None |
| WGCV-04725 | male | Songkhla | 14/10/2021 | 49 | NA | AY.30 |
| WGCV-04726 | female | Songkhla | 14/10/2021 | 50 | NA | AY.30 |
| WGCV-04727 | female | Songkhla | 15/10/2021 | 45 | NA | B.1.617.2 |
| WGCV-04730 | female | Narathiwat | 9/10/2021 | 30 | NA | B.1.617.2 |
| WGCV-04731 | male | Songkhla | 11/10/2021 | 38 | NA | AY.30 |
| WGCV-04732 | male | Songkhla | 11/10/2021 | 35 | NA | AY.30 |
| WGCV-04733 | female | Songkhla | 14/10/2021 | 26 | NA | AY.30 |
| WGCV-04734 | female | Songkhla | 14/10/2021 | 17 | NA | B.1.617.2 |
| WGCV-04737 | male | Narathiwat | 9/10/2021 | 21 | NA | B.1.617.2 |
| WGCV-04738 | male | Songkhla | 11/10/2021 | 57 | NA | B.1.617.2 |
| WGCV-04739 | male | Songkhla | 11/10/2021 | 34 | NA | AY.30 |
| WGCV-04740 | male | Songkhla | 14/10/2021 | 42 | NA | AY.30 |
| WGCV-04741 | male | Songkhla | 14/10/2021 | 58 | NA | AY.30 |
| WGCV-04742 | male | Songkhla | 15/10/2021 | 34 | NA | B.1.617.2 |
| WGCV-04743 | female | Yala | 10/10/2021 | 41 | NA | B.1.617.2 |
| WGCV-04744 | male | Pattani | 30/12/2021 | NA | NA | B.1.617.2 |
| WGCV-04745 | male | Pattani | 15/10/2021 | NA | NA | B.1.617.2 |
| WGCV-04747 | male | Narathiwat | 13/10/2021 | NA | NA | B.1.617.2 |
| WGCV-04748 | female | Narathiwat | 16/10/2021 | NA | NA | AY.30 |
| WGCV-04750 | male | Songkhla | 15/10/2021 | NA | NA | AY.30 |
| WGCV-04751 | male | Yala | 11/10/2021 | NA | NA | B.1.617.2 |
| WGCV-04753 | male | Pattani | 30/12/2021 | NA | NA | B.1.617.2 |
| WGCV-04754 | female | Narathiwat | 11/10/2021 | NA | NA | B.1.617.2 |
| WGCV-04755 | male | Narathiwat | 14/10/2021 | NA | NA | B.1.617.2 |
| WGCV-04756 | male | Narathiwat | 16/10/2021 | NA | NA | AY.30 |
| WGCV-04758 | female | Narathiwat | 11/10/2021 | NA | NA | B.1.351 |
| WGCV-04759 | female | Yala | 11/10/2021 | NA | NA | B.1.617.2 |
| WGCV-04760 | female | Pattani | 15/10/2021 | NA | NA | B.1.617.2 |
| WGCV-04761 | female | Pattani | 15/10/2021 | NA | NA | B.1.617.2 |
| WGCV-04762 | male | Narathiwat | 12/10/2021 | NA | NA | B.1.617.2 |
| WGCV-04763 | male | Narathiwat | 14/10/2021 | NA | NA | B.1.617.2 |
| WGCV-04764 | female | Narathiwat | 16/10/2021 | NA | NA | B.1.617.2 |
| WGCV-04766 | male | Narathiwat | 11/10/2021 | NA | NA | B.1.617.2 |
| WGCV-04767 | male | Yala | 16/10/2021 | NA | NA | B.1.617.2 |
| WGCV-04768 | female | Pattani | 15/10/2021 | NA | NA | B.1.617.2 |
| WGCV-04769 | male | Pattani | 15/10/2021 | NA | NA | B.1.617.2 |
| WGCV-04770 | male | Narathiwat | 12/10/2021 | NA | NA | B.1.617.2 |
| WGCV-04771 | male | Narathiwat | 14/10/2021 | NA | NA | AY.30 |
| WGCV-04772 | female | Narathiwat | 19/10/2021 | NA | NA | B.1.617.2 |
| WGCV-04774 | female | Narathiwat | 11/10/2021 | NA | NA | B.1.617.2 |
| WGCV-04775 | female | Yala | 17/10/2021 | NA | NA | B.1.617.2 |
| WGCV-04776 | female | Pattani | 15/10/2021 | NA | NA | B.1.617.2 |
| WGCV-04777 | male | Pattani | 15/10/2021 | NA | NA | B.1.617.2 |
| WGCV-04778 | male | Narathiwat | 10/10/2021 | NA | NA | B.1.617.2 |
| WGCV-04779 | male | Narathiwat | 14/10/2021 | NA | NA | B.1.617.2 |
| WGCV-04780 | female | Narathiwat | 16/10/2021 | NA | NA | B.1.617.2 |
| WGCV-04782 | female | Narathiwat | 11/10/2021 | NA | NA | B.1.617.2 |
| WGCV-04783 | female | Yala | 17/10/2021 | NA | NA | B.1.617.2 |
| WGCV-04784 | male | Pattani | 30/12/2021 | NA | NA | B.1.617.2 |
| WGCV-04785 | male | Pattani | 15/10/2021 | NA | NA | B.1.617.2 |
| WGCV-04786 | female | Narathiwat | 13/10/2021 | NA | NA | B.1.617.2 |
| WGCV-04787 | female | Narathiwat | 14/10/2021 | NA | NA | AY.30 |
| WGCV-04788 | male | Narathiwat | 19/10/2021 | NA | NA | B.1.617.2 |
| WGCV-04790 | female | Yala | 6/10/2021 | NA | NA | B.1.617.2 |
| WGCV-04791 | female | Yala | 17/10/2021 | NA | NA | B.1.617.2 |
| WGCV-04792 | male | Pattani | 15/10/2021 | NA | NA | B.1.617.2 |
| WGCV-04793 | male | Pattani | 15/10/2021 | NA | NA | B.1.617.2 |
| WGCV-04794 | female | Narathiwat | 18/10/2021 | NA | NA | B.1.617.2 |
| WGCV-04795 | female | Narathiwat | 15/10/2021 | NA | NA | B.1.617.2 |
| WGCV-04798 | female | Yala | 7/10/2021 | NA | NA | B.1.617.2 |
| WGCV-04799 | female | Pattani | 30/12/2021 | NA | NA | B.1.617.2 |
| WGCV-04800 | female | Pattani | 30/12/2021 | NA | NA | B.1.617.2 |
| WGCV-04801 | male | Pattani | 30/12/2021 | NA | NA | B.1.617.2 |
| WGCV-04802 | male | Narathiwat | 13/10/2021 | NA | NA | B.1.617.2 |
| WGCV-04803 | female | Narathiwat | 15/10/2021 | NA | NA | B.1.617.2 |
| WGCV-04806 | female | Yala | 7/10/2021 | NA | NA | B.1.617.2 |
| WGCV-04807 | male | Pattani | 15/10/2021 | NA | NA | AY.30 |
| WGCV-04808 | female | Pattani | 30/12/2021 | NA | NA | B.1.617.2 |
| WGCV-04809 | male | Pattani | 30/12/2021 | NA | NA | AY.30 |
| WGCV-04810 | male | Narathiwat | 13/10/2021 | NA | NA | B.1.617.2 |
| WGCV-04811 | male | Narathiwat | 15/10/2021 | NA | NA | B.1.617.2 |
| WGCV-04814 | female | Yala | 7/10/2021 | NA | NA | B.1.617.2 |
| WGCV-04815 | male | Pattani | 30/12/2021 | NA | NA | B.1.617.2 |
| WGCV-04816 | male | Pattani | 30/12/2021 | NA | NA | B.1.617.2 |
| WGCV-04817 | female | Pattani | 30/12/2021 | NA | NA | B.1.617.2 |
| WGCV-04818 | female | Narathiwat | 15/10/2021 | NA | NA | B.1.617.2 |
| WGCV-04819 | male | Narathiwat | 15/10/2021 | NA | NA | B.1.617.2 |
| WGCV-04822 | male | Yala | 8/10/2021 | NA | NA | B.1.617.2 |
| WGCV-04823 | female | Pattani | 14/10/2021 | NA | NA | B.1.617.2 |
| WGCV-04824 | female | Pattani | 30/12/2021 | NA | NA | B.1.617.2 |
| WGCV-04825 | male | Narathiwat | 18/10/2021 | NA | NA | B.1.617.2 |
| WGCV-04826 | female | Narathiwat | 17/10/2021 | NA | NA | AY.38 |
| WGCV-04827 | female | Narathiwat | 16/10/2021 | NA | NA | B.1.617.2 |
| WGCV-04829 | female | Yala | 8/10/2021 | NA | NA | B.1.617.2 |
| WGCV-04830 | female | Pattani | 30/12/2021 | NA | NA | B.1.617.2 |
| WGCV-04831 | female | Pattani | 30/12/2021 | NA | NA | B.1.617.2 |
| WGCV-04832 | male | Narathiwat | 14/10/2021 | NA | NA | B.1.617.2 |
| WGCV-04833 | female | Narathiwat | 13/10/2021 | NA | NA | B.1.617.2 |
| WGCV-04834 | female | Narathiwat | 17/10/2021 | NA | NA | B.1.1.7 |
| WGCV-04930 | male | Narathiwat | 16/10/2021 | NA | NA | B.1.617.2 |
| WGCV-04931 | male | Narathiwat | 15/10/2021 | NA | NA | B.1.617.2 |
| WGCV-04932 | female | Narathiwat | 15/10/2021 | NA | NA | B.1.617.2 |
| WGCV-04933 | male | Songkhla | 18/10/2021 | NA | NA | AY.30 |
| WGCV-04934 | male | Songkhla | 19/10/2021 | NA | NA | B.1.617.2 |
| WGCV-04935 | female | Yala | 19/10/2021 | NA | NA | B.1.617.2 |
| WGCV-04936 | male | Yala | 18/10/2021 | NA | NA | B.1.617.2 |
| WGCV-04937 | female | Yala | 18/10/2021 | NA | NA | B.1.617.2 |
| WGCV-04938 | male | Narathiwat | 16/10/2021 | NA | NA | AY.30 |
| WGCV-04939 | male | Narathiwat | 15/10/2021 | NA | NA | B.1.617.2 |
| WGCV-04940 | female | Narathiwat | 15/10/2021 | NA | NA | B.1.617.2 |
| WGCV-04941 | male | Songkhla | 18/10/2021 | NA | NA | AY.30 |
| WGCV-04942 | male | Songkhla | 19/10/2021 | NA | NA | B.1.617.2 |
| WGCV-04943 | male | Yala | 18/10/2021 | NA | NA | B.1.617.2 |
| WGCV-04944 | female | Yala | 18/10/2021 | NA | NA | B.1.617.2 |
| WGCV-04945 | male | Yala | 18/10/2021 | NA | NA | B.1.617.2 |
| WGCV-04946 | male | Songkhla | 16/10/2021 | NA | NA | B.1.617.2 |
| WGCV-04947 | female | Narathiwat | 15/10/2021 | NA | NA | B.1.617.2 |
| WGCV-04948 | male | Narathiwat | 15/10/2021 | NA | NA | B.1.617.2 |
| WGCV-04949 | male | Songkhla | 18/10/2021 | NA | NA | AY.30 |
| WGCV-04950 | male | Songkhla | 19/10/2021 | NA | NA | B.1.617.2 |
| WGCV-04951 | female | Yala | 18/10/2021 | NA | NA | B.1.617.2 |
| WGCV-04952 | female | Yala | 18/10/2021 | NA | NA | B.1.617.2 |
| WGCV-04953 | male | Yala | 18/10/2021 | NA | NA | B.1.617.2 |
| WGCV-04954 | male | Narathiwat | 16/10/2021 | NA | NA | B.1.617.2 |
| WGCV-04955 | male | Narathiwat | 15/10/2021 | NA | NA | B.1.617.2 |
| WGCV-04956 | male | Narathiwat | 15/10/2021 | NA | NA | AY.39 |
| WGCV-04957 | male | Songkhla | 18/10/2021 | NA | NA | B.1.617.2 |
| WGCV-04958 | female | Songkhla | 19/10/2021 | NA | NA | B.1.617.2 |
| WGCV-04959 | male | Yala | 18/10/2021 | NA | NA | B.1.617.2 |
| WGCV-04960 | female | Yala | 18/10/2021 | NA | NA | B.1.617.2 |
| WGCV-04961 | male | Yala | 19/10/2021 | NA | NA | B.1.617.2 |
| WGCV-04962 | male | Narathiwat | 16/10/2021 | NA | NA | B.1.617.2 |
| WGCV-04963 | female | Narathiwat | 15/10/2021 | NA | NA | B.1.617.2 |
| WGCV-04964 | female | Narathiwat | 15/10/2021 | NA | NA | B.1.617.2 |
| WGCV-04965 | female | Songkhla | 18/10/2021 | NA | NA | AY.30 |
| WGCV-04966 | female | Songkhla | 19/10/2021 | NA | NA | AY.39 |
| WGCV-04967 | female | Yala | 18/10/2021 | NA | NA | B.1.617.2 |
| WGCV-04968 | female | Yala | 18/10/2021 | NA | NA | B.1.617.2 |
| WGCV-04969 | male | Yala | 19/10/2021 | NA | NA | B.1.617.2 |
| WGCV-04970 | male | Songkhla | 16/10/2021 | NA | NA | B.1.617.2 |
| WGCV-04971 | male | Narathiwat | 15/10/2021 | NA | NA | B.1.617.2 |
| WGCV-04972 | female | Narathiwat | 15/10/2021 | NA | NA | B.1.617.2 |
| WGCV-04973 | female | Songkhla | 18/10/2021 | NA | NA | AY.30 |
| WGCV-04974 | male | Songkhla | 19/10/2021 | NA | NA | AY.30 |
| WGCV-04975 | male | Yala | 18/10/2021 | NA | NA | B.1.617.2 |
| WGCV-04976 | male | Yala | 18/10/2021 | NA | NA | B.1.617.2 |
| WGCV-04977 | male | Yala | 19/10/2021 | NA | NA | B.1.617.2 |
| WGCV-04978 | male | Songkhla | 16/10/2021 | NA | NA | B.1.617.2 |
| WGCV-04979 | female | Narathiwat | 15/10/2021 | NA | NA | B.1.617.2 |
| WGCV-04980 | female | Narathiwat | 15/10/2021 | NA | NA | B.1.617.2 |
| WGCV-04981 | female | Songkhla | 18/10/2021 | NA | NA | AY.39 |
| WGCV-04982 | female | Songkhla | 19/10/2021 | NA | NA | AY.30 |
| WGCV-04983 | male | Yala | 18/10/2021 | NA | NA | B.1.617.2 |
| WGCV-04984 | male | Yala | 18/10/2021 | NA | NA | B.1.617.2 |
| WGCV-04985 | male | Yala | 19/10/2021 | NA | NA | B.1.617.2 |
| WGCV-04986 | female | Narathiwat | 15/10/2021 | NA | NA | B.1.617.2 |
| WGCV-04987 | male | Narathiwat | 15/10/2021 | NA | NA | B.1.617.2 |
| WGCV-04988 | male | Narathiwat | 15/10/2021 | NA | NA | B.1.617.2 |
| WGCV-04989 | male | Songkhla | 18/10/2021 | NA | NA | AY.30 |
| WGCV-04990 | male | Songkhla | 19/10/2021 | NA | NA | B.1.617.2 |
| WGCV-04991 | male | Yala | 18/10/2021 | NA | NA | B.1.617.2 |
| WGCV-04992 | female | Yala | 18/10/2021 | NA | NA | B.1.617.2 |
| WGCV-04993 | male | Yala | 19/10/2021 | NA | NA | B.1.617.2 |
| WGCV-04994 | female | Narathiwat | 15/10/2021 | NA | NA | B.1.617.2 |
| WGCV-04995 | male | Narathiwat | 15/10/2021 | NA | NA | B.1.617.2 |
| WGCV-04996 | male | Songkhla | 18/10/2021 | NA | NA | B.1.617.2 |
| WGCV-04997 | female | Songkhla | 18/10/2021 | NA | NA | B.1.617.2 |
| WGCV-04998 | female | Yala | 18/10/2021 | NA | NA | B.1.617.2 |
| WGCV-04999 | female | Yala | 18/10/2021 | NA | NA | B.1.617.2 |
| WGCV-05000 | female | Yala | 18/10/2021 | NA | NA | B.1.617.2 |
| WGCV-05001 | male | Yala | 19/10/2021 | NA | NA | B.1.617.2 |
| WGCV-05002 | female | Narathiwat | 15/10/2021 | NA | NA | B.1.617.2 |
| WGCV-05003 | female | Narathiwat | 15/10/2021 | NA | NA | B.1.617.2 |
| WGCV-05004 | female | Songkhla | 18/10/2021 | NA | NA | B.1.617.2 |
| WGCV-05005 | female | Songkhla | 18/10/2021 | NA | NA | B.1.617.2 |
| WGCV-05006 | female | Yala | 18/10/2021 | NA | NA | B.1.617.2 |
| WGCV-05007 | male | Yala | 18/10/2021 | NA | NA | B.1.617.2 |
| WGCV-05008 | male | Yala | 18/10/2021 | NA | NA | B.1.617.2 |
| WGCV-05009 | female | Yala | 19/10/2021 | NA | NA | B.1.617.2 |
| WGCV-05010 | female | Narathiwat | 15/10/2021 | NA | NA | B.1.617.2 |
| WGCV-05011 | female | Narathiwat | 15/10/2021 | NA | NA | B.1.617.2 |
| WGCV-05012 | male | Songkhla | 18/10/2021 | NA | NA | B.1.617.2 |
| WGCV-05013 | female | Songkhla | 19/10/2021 | NA | NA | B.1.617.2 |
| WGCV-05014 | male | Yala | 18/10/2021 | NA | NA | B.1.617.2 |
| WGCV-05015 | male | Yala | 18/10/2021 | NA | NA | B.1.617.2 |
| WGCV-05016 | male | Yala | 18/10/2021 | NA | NA | B.1.617.2 |
| WGCV-05017 | female | Narathiwat | 15/10/2021 | NA | NA | B.1.617.2 |
| WGCV-05018 | male | Narathiwat | 15/10/2021 | NA | NA | B.1.617.2 |
| WGCV-05020 | male | Songkhla | 19/10/2021 | NA | NA | B.1.617.2 |
| WGCV-05021 | female | Yala | 18/10/2021 | NA | NA | B.1.617.2 |
| WGCV-05022 | female | Yala | 18/10/2021 | NA | NA | B.1.617.2 |
| WGCV-05023 | male | Yala | 18/10/2021 | NA | NA | B.1.617.2 |
| WGCV-05024 | female | Yala | 19/10/2021 | NA | NA | AY.29 |
| WGCV-05025 | female | Yala | 19/10/2021 | NA | NA | B.1.617.2 |
| WGCV-05026 | female | Yala | 19/10/2021 | NA | NA | B.1.617.2 |
| WGCV-05027 | male | Yala | 19/10/2021 | NA | NA | AY.30 |
| WGCV-05028 | male | Yala | 19/10/2021 | NA | NA | B.1.617.2 |
| WGCV-05029 | female | Pattani | 14/10/2021 | NA | NA | B.1.617.2 |
| WGCV-05030 | female | Pattani | 18/10/2021 | NA | NA | B.1.617.2 |
| WGCV-05031 | female | Narathiwat | 19/10/2021 | NA | NA | B.1.617.2 |
| WGCV-05032 | female | Yala | 19/10/2021 | NA | NA | B.1.617.2 |
| WGCV-05033 | female | Yala | 19/10/2021 | NA | NA | B.1.617.2 |
| WGCV-05034 | female | Yala | 19/10/2021 | NA | NA | B.1.617.2 |
| WGCV-05035 | male | Yala | 19/10/2021 | NA | NA | B.1.617.2 |
| WGCV-05036 | female | Yala | 14/10/2021 | NA | NA | AY.39 |
| WGCV-05037 | female | Pattani | 14/10/2021 | NA | NA | B.1.617.2 |
| WGCV-05038 | male | Pattani | 19/10/2021 | NA | NA | B.1.617.2 |
| WGCV-05039 | male | Narathiwat | 19/10/2021 | NA | NA | B.1.617.2 |
| WGCV-05040 | female | Yala | 19/10/2021 | NA | NA | B.1.617.2 |
| WGCV-05041 | female | Yala | 19/10/2021 | NA | NA | B.1.617.2 |
| WGCV-05043 | male | Yala | 19/10/2021 | NA | NA | B.1.617.2 |
| WGCV-05044 | female | Pattani | 5/10/2021 | NA | NA | AY.46 |
| WGCV-05045 | female | Pattani | 15/10/2021 | NA | NA | B.1.617.2 |
| WGCV-05046 | male | Pattani | 19/10/2021 | NA | NA | B.1.617.2 |
| WGCV-05048 | male | Yala | 19/10/2021 | NA | NA | B.1.617.2 |
| WGCV-05049 | female | Yala | 19/10/2021 | NA | NA | B.1.617.2 |
| WGCV-05050 | female | Yala | 19/10/2021 | NA | NA | B.1.617.2 |
| WGCV-05051 | female | Yala | 19/10/2021 | NA | NA | B.1.617.2 |
| WGCV-05052 | female | Pattani | 7/10/2021 | NA | NA | B.1.617.2 |
| WGCV-05053 | female | Pattani | 15/10/2021 | NA | NA | AY.46 |
| WGCV-05056 | female | Yala | 19/10/2021 | NA | NA | B.1.617.2 |
| WGCV-05057 | male | Yala | 19/10/2021 | NA | NA | B.1.617.2 |
| WGCV-05058 | female | Yala | 19/10/2021 | NA | NA | B.1.617.2 |
| WGCV-05059 | female | Yala | 19/10/2021 | NA | NA | B.1.617.2 |
| WGCV-05060 | female | Pattani | 8/10/2021 | NA | NA | B.1.617.2 |
| WGCV-05061 | female | Pattani | 16/10/2021 | NA | NA | B.1.617.2 |
| WGCV-05062 | female | Pattani | 19/10/2021 | NA | NA | B.1.617.2 |
| WGCV-05063 | male | Narathiwat | 19/10/2021 | NA | NA | B.1.617.2 |
| WGCV-05064 | male | Yala | 19/10/2021 | NA | NA | B.1.617.2 |
| WGCV-05065 | female | Yala | 19/10/2021 | NA | NA | B.1.617.2 |
| WGCV-05066 | female | Yala | 19/10/2021 | NA | NA | B.1.617.2 |
| WGCV-05067 | male | Yala | 19/10/2021 | NA | NA | AY.30 |
| WGCV-05068 | female | Pattani | 8/10/2021 | NA | NA | B.1.617.2 |
| WGCV-05070 | female | Narathiwat | 19/10/2021 | NA | NA | B.1.617.2 |
| WGCV-05071 | male | Narathiwat | 19/10/2021 | NA | NA | B.1.617.2 |
| WGCV-05072 | male | Yala | 19/10/2021 | NA | NA | B.1.617.2 |
| WGCV-05073 | female | Yala | 19/10/2021 | NA | NA | B.1.617.2 |
| WGCV-05074 | male | Yala | 19/10/2021 | NA | NA | B.1.617.2 |
| WGCV-05075 | male | Yala | 19/10/2021 | NA | NA | B.1.617.2 |
| WGCV-05076 | male | Pattani | 10/10/2021 | NA | NA | B.1.617.2 |
| WGCV-05077 | female | Pattani | 17/10/2021 | NA | NA | B.1.617.2 |
| WGCV-05078 | male | Narathiwat | 19/10/2021 | NA | NA | AY.30 |
| WGCV-05079 | female | Narathiwat | 19/10/2021 | NA | NA | B.1.617.2 |
| WGCV-05080 | female | Yala | 19/10/2021 | NA | NA | AY.39 |
| WGCV-05081 | female | Yala | 19/10/2021 | NA | NA | B.1.617.2 |
| WGCV-05082 | female | Yala | 19/10/2021 | NA | NA | B.1.617.2 |
| WGCV-05083 | female | Yala | 19/10/2021 | NA | NA | B.1.617.2 |
| WGCV-05084 | female | Pattani | 14/10/2021 | NA | NA | B.1.617.2 |
| WGCV-05085 | female | Pattani | 17/10/2021 | NA | NA | B.1.617.2 |
| WGCV-05086 | female | Narathiwat | 21/10/2021 | NA | NA | B.1.617.2 |
| WGCV-05087 | female | Narathiwat | 19/10/2021 | NA | NA | B.1.617.2 |
| WGCV-05088 | male | Yala | 19/10/2021 | NA | NA | B.1.617.2 |
| WGCV-05089 | female | Yala | 19/10/2021 | NA | NA | B.1.617.2 |
| WGCV-05090 | female | Yala | 19/10/2021 | NA | NA | AY.44 |
| WGCV-05091 | female | Yala | 19/10/2021 | NA | NA | B.1.617.2 |
| WGCV-05092 | female | Pattani | 11/10/2021 | NA | NA | B.1.617.2 |
| WGCV-05093 | male | Pattani | 17/10/2021 | NA | NA | B.1.617.2 |
| WGCV-05094 | female | Narathiwat | 21/10/2021 | NA | NA | B.1.617.2 |
| WGCV-05095 | female | Narathiwat | 19/10/2021 | NA | NA | B.1.617.2 |
| WGCV-05096 | male | Yala | 19/10/2021 | NA | NA | B.1.617.2 |
| WGCV-05098 | male | Yala | 19/10/2021 | NA | NA | AY.29 |
| WGCV-05099 | male | Yala | 19/10/2021 | NA | NA | B.1.617.2 |
| WGCV-05100 | male | Pattani | 12/10/2021 | NA | NA | B.1.617.2 |
| WGCV-05101 | female | Pattani | 17/10/2021 | NA | NA | B.1.617.2 |
| WGCV-05102 | female | Narathiwat | 20/10/2021 | NA | NA | B.1.617.2 |
| WGCV-05103 | male | Narathiwat | 19/10/2021 | NA | NA | B.1.617.2 |
| WGCV-05104 | male | Yala | 19/10/2021 | NA | NA | B.1.617.2 |
| WGCV-05105 | male | Yala | 19/10/2021 | NA | NA | B.1.617.2 |
| WGCV-05106 | male | Yala | 19/10/2021 | NA | NA | B.1.617.2 |
| WGCV-05107 | male | Yala | 19/10/2021 | NA | NA | B.1.617.2 |
| WGCV-05108 | male | Pattani | 12/10/2021 | NA | NA | B.1.617.2 |
| WGCV-05109 | male | Pattani | 18/10/2021 | NA | NA | AY.30 |
| WGCV-05110 | female | Narathiwat | 19/10/2021 | NA | NA | B.1.617.2 |
| WGCV-05111 | female | Yala | 19/10/2021 | NA | NA | B.1.617.2 |
| WGCV-05112 | female | Yala | 19/10/2021 | NA | NA | B.1.617.2 |
| WGCV-05113 | female | Yala | 19/10/2021 | NA | NA | B.1.617.2 |
| WGCV-05114 | male | Yala | 19/10/2021 | NA | NA | B.1.617.2 |
| WGCV-05115 | female | Pattani | 13/10/2021 | NA | NA | AY.39 |
| WGCV-05116 | female | Pattani | 18/10/2021 | NA | NA | B.1.617.2 |
| WGCV-05117 | female | Narathiwat | 19/10/2021 | NA | NA | B.1.617.2 |
| WGCV-05118 | male | Narathiwat | 19/10/2021 | NA | NA | B.1.617.2 |
| WGCV-05119 | male | Narathiwat | 20/10/2021 | NA | NA | B.1.617.2 |
| WGCV-05120 | female | Narathiwat | 21/10/2021 | NA | NA | B.1.617.2 |
| WGCV-05121 | female | Narathiwat | 13/10/2021 | NA | NA | AY.30 |
| WGCV-05126 | female | Narathiwat | 19/10/2021 | NA | NA | B.1.617.2 |
| WGCV-05127 | female | Narathiwat | 16/10/2021 | NA | NA | B.1.1.7 |
| WGCV-05128 | male | Narathiwat | 21/10/2021 | NA | NA | B.1.617.2 |
| WGCV-05129 | female | Narathiwat | 14/10/2021 | NA | NA | B.1.617.2 |
| WGCV-05134 | male | Narathiwat | 19/10/2021 | NA | NA | B.1.617.2 |
| WGCV-05135 | male | Narathiwat | 20/10/2021 | NA | NA | B.1.617.2 |
| WGCV-05136 | male | Narathiwat | 21/10/2021 | NA | NA | B.1.617.2 |
| WGCV-05137 | female | Narathiwat | 14/10/2021 | NA | NA | B.1.617.2 |
| WGCV-05142 | male | Narathiwat | 19/10/2021 | NA | NA | B.1.617.2 |
| WGCV-05143 | female | Narathiwat | 20/10/2021 | NA | NA | B.1.617.2 |
| WGCV-05144 | female | Narathiwat | 20/10/2021 | NA | NA | B.1.617.2 |
| WGCV-05145 | female | Narathiwat | 14/10/2021 | NA | NA | AY.30 |
| WGCV-05150 | female | Narathiwat | 19/10/2021 | NA | NA | B.1.617.2 |
| WGCV-05151 | female | Narathiwat | 20/10/2021 | NA | NA | AY.30 |
| WGCV-05152 | female | Narathiwat | 3/10/2021 | NA | NA | B.1.617.2 |
| WGCV-05153 | male | Narathiwat | 14/10/2021 | NA | NA | B.1.617.2 |
| WGCV-05158 | male | Narathiwat | 19/10/2021 | NA | NA | B.1.617.2 |
| WGCV-05159 | female | Narathiwat | 21/10/2021 | NA | NA | B.1.617.2 |
| WGCV-05160 | female | Narathiwat | 6/10/2021 | NA | NA | B.1.617.2 |
| WGCV-05161 | male | Narathiwat | 14/10/2021 | NA | NA | B.1.617.2 |
| WGCV-05166 | male | Narathiwat | 19/10/2021 | NA | NA | B.1.617.2 |
| WGCV-05167 | male | Narathiwat | 21/10/2021 | NA | NA | B.1.617.2 |
| WGCV-05168 | female | Narathiwat | 10/10/2021 | NA | NA | AY.39 |
| WGCV-05169 | female | Narathiwat | 16/10/2021 | NA | NA | AY.39 |
| WGCV-05174 | male | Narathiwat | 19/10/2021 | NA | NA | B.1.617.2 |
| WGCV-05175 | female | Narathiwat | 21/10/2021 | NA | NA | B.1.617.2 |
| WGCV-05176 | male | Narathiwat | 11/10/2021 | NA | NA | B.1.617.2 |
| WGCV-05177 | female | Narathiwat | 17/10/2021 | NA | NA | AY.39 |
| WGCV-05182 | male | Narathiwat | 19/10/2021 | NA | NA | B.1.617.2 |
| WGCV-05184 | female | Narathiwat | 12/10/2021 | NA | NA | B.1.617.2 |
| WGCV-05185 | female | Narathiwat | 18/10/2021 | NA | NA | AY.39 |
| WGCV-05190 | female | Narathiwat | 19/10/2021 | NA | NA | B.1.617.2 |
| WGCV-05191 | female | Narathiwat | 16/10/2021 | NA | NA | B.1.617.2 |
| WGCV-05192 | female | Narathiwat | 13/10/2021 | NA | NA | B.1.617.2 |
| WGCV-05193 | female | Narathiwat | 18/10/2021 | NA | NA | B.1.617.2 |
| WGCV-05198 | male | Narathiwat | 19/10/2021 | NA | NA | B.1.617.2 |
| WGCV-05199 | female | Narathiwat | 18/10/2021 | NA | NA | B.1.617.2 |
| WGCV-05200 | male | Narathiwat | 13/10/2021 | NA | NA | B.1.617.2 |
| WGCV-05201 | female | Narathiwat | 18/10/2021 | NA | NA | AY.30 |
| WGCV-05205 | female | Narathiwat | 19/10/2021 | NA | NA | B.1.617.2 |
| WGCV-05206 | female | Narathiwat | 20/10/2021 | NA | NA | B.1.617.2 |
| WGCV-05207 | female | Narathiwat | 13/10/2021 | NA | NA | B.1.617.2 |
| WGCV-05208 | male | Narathiwat | 18/10/2021 | NA | NA | B.1.617.2 |

**Supplementary Table S2** Metadata of AY.85 sequences from the FSP area.

| **Code** | **Sex** | **Age** | **AY.85 variant** | **Infection outcome** | **Province** | **Collection date** |
| --- | --- | --- | --- | --- | --- | --- |
| COVPSU-00380 | female | 55 | S: F306 | Alive | Narathiwat | 19/10/2021 |
| COVPSU-00382 | female | 69 | S: F306 | Alive | Narathiwat | 19/10/2021 |
| COVPSU-00383 | female | 17 | S: L306 | Alive | Narathiwat | 19/10/2021 |
| COVPSU-00384 | male | 56 | S: L306 | Alive | Narathiwat | 20/10/2021 |
| COVPSU-00385 | male | 15 | S: F306 | Alive | Narathiwat | 20/10/2021 |
| COVPSU-00386 | female | 51 | S: L306 | Alive | Narathiwat | 20/10/2021 |
| COVPSU-00387 | female | 29 | S: F306 | Alive | Narathiwat | 20/10/2021 |
| COVPSU-00388 | female | 43 | S: F306 | Alive | Narathiwat | 20/10/2021 |
| COVPSU-00390 | male | 56 | S: F306 | Alive | Narathiwat | 20/10/2021 |
| COVPSU-00391 | male | 24 | S: L306 | Alive | Narathiwat | 20/10/2021 |
| COVPSU-00392 | female | 85 | S: L306 | Alive | Narathiwat | 20/10/2021 |
| COVPSU-00393 | male | 39 | S: L306 | Alive | Narathiwat | 9/10/2021 |
| COVPSU-00394 | male | 55 | S: F306 | Alive | Narathiwat | 20/10/2021 |
| COVPSU-00395 | male | 19 | S: F306 | Alive | Narathiwat | 21/10/2021 |
| COVPSU-00397 | female | 19 | S: F306 | Alive | Narathiwat | 20/10/2021 |
| COVPSU-00398 | male | 15 | S: F306 | Alive | Narathiwat | 21/10/2021 |
| COVPSU-00401 | female | 34 | S: L306 | Alive | Narathiwat | 4/10/2021 |
| COVPSU-00402 | male | 42 | S: F306 | Alive | Narathiwat | 22/10/2021 |
| COVPSU-00403 | male | 43 | S: F306 | Alive | Narathiwat | 24/10/2021 |
| COVPSU-00404 | female | 32 | S: F306 | Alive | Narathiwat | 24/10/2021 |
| COVPSU-00405 | male | 40 | S: F306 | Alive | Narathiwat | 24/10/2021 |
| COVPSU-00406 | female | 62 | S: F306 | Alive | Narathiwat | 24/10/2021 |
| COVPSU-00407 | female | 26 | S: F306 | Alive | Narathiwat | 25/10/2021 |
| COVPSU-00408 | male | 60 | S: F306 | Alive | Narathiwat | 25/10/2021 |
| COVPSU-00410 | female | 73 | S: F306 | Alive | Narathiwat | 25/10/2021 |
| COVPSU-00411 | female | 57 | S: F306 | Alive | Narathiwat | 25/10/2021 |
| COVPSU-00413 | female | 51 | S: F306 | Alive | Narathiwat | 25/10/2021 |
| COVPSU-00414 | female | 34 | S: F306 | Alive | Narathiwat | 25/10/2021 |
| COVPSU-00415 | female | 49 | S: L306 | Alive | Narathiwat | 25/10/2021 |
| COVPSU-00416 | female | 23 | S: F306 | Alive | Narathiwat | 25/10/2021 |
| COVPSU-00417 | female | 12 | S: L306 | Alive | Narathiwat | 25/10/2021 |
| COVPSU-00418 | male | 60 | S: L306 | Alive | Narathiwat | 25/10/2021 |
| COVPSU-00419 | female | 72 | S: F306 | Alive | Narathiwat | 25/10/2021 |
| COVPSU-00420 | female | 39 | S: F306 | Alive | Narathiwat | 26/10/2021 |
| COVPSU-00421 | male | 41 | S: F306 | Alive | Yala | 26/10/2021 |
| COVPSU-00422 | female | 34 | S: F306 | Alive | Narathiwat | 26/10/2021 |
| COVPSU-00424 | female | 60 | S: F306 | Alive | Narathiwat | 26/10/2021 |
| COVPSU-00425 | male | 59 | S: L306 | Alive | Narathiwat | 26/10/2021 |
| COVPSU-00426 | male | 42 | S: F306 | Alive | Narathiwat | 26/10/2021 |
| COVPSU-00427 | male | 41 | S: F306 | Alive | Narathiwat | 26/10/2021 |
| COVPSU-00428 | female | 30 | S: F306 | Alive | Narathiwat | 26/10/2021 |
| COVPSU-00429 | female | 64 | S: L306 | Alive | Narathiwat | 28/10/2021 |
| COVPSU-00430 | female | 26 | S: F306 | Alive | Narathiwat | 28/10/2021 |
| COVPSU-00432 | female | 30 | S: F306 | Alive | Narathiwat | 28/10/2021 |
| COVPSU-00433 | male | 49 | S: F306 | Alive | Narathiwat | 28/10/2021 |
| COVPSU-00434 | female | 40 | S: L306 | Alive | Narathiwat | 28/10/2021 |
| COVPSU-00435 | female | 60 | S: F306 | Alive | Narathiwat | 28/10/2021 |
| COVPSU-00437 | male | 58 | S: F306 | Alive | Narathiwat | 29/10/2021 |
| COVPSU-00438 | female | 36 | S: F306 | Alive | Narathiwat | 29/10/2021 |
| COVPSU-00439 | female | 79 | S: F306 | Alive | Narathiwat | 29/10/2021 |
| COVPSU-00440 | male | 23 | S: F306 | Alive | Narathiwat | 29/10/2021 |
| COVPSU-00442 | female | 71 | S: F306 | Alive | Narathiwat | 29/10/2021 |
| COVPSU-00443 | female | 61 | S: F306 | Alive | Narathiwat | 29/10/2021 |
| COVPSU-00444 | female | 13 | S: F306 | Alive | Narathiwat | 29/10/2021 |
| COVPSU-00445 | female | 42 | S: L306 | Alive | Narathiwat | 29/10/2021 |
| COVPSU-00447 | male | 46 | S: F306 | Alive | Narathiwat | 29/10/2021 |
| COVPSU-00448 | male | 39 | S: F306 | Alive | Narathiwat | 29/10/2021 |
| COVPSU-00449 | male | 90 | S: F306 | Alive | Narathiwat | 29/10/2021 |
| COVPSU-00450 | female | 60 | S: L306 | Alive | Narathiwat | 31/10/2021 |
| COVPSU-00452 | female | 35 | S: F306 | Alive | Narathiwat | 1/11/2021 |
| COVPSU-00453 | female | 68 | S: F306 | Alive | Narathiwat | 1/11/2021 |
| COVPSU-00454 | female | 28 | S: F306 | Alive | Narathiwat | 1/11/2021 |
| COVPSU-00455 | male | 17 | S: F306 | Alive | Phatthalung | 1/11/2021 |
| COVPSU-00456 | female | 36 | S: F306 | Alive | Narathiwat | 20/10/2021 |
| COVPSU-00457 | male | 67 | S: F306 | Alive | Narathiwat | 1/11/2021 |
| COVPSU-00459 | male | 37 | S: F306 | Alive | Phatthalung | 1/11/2021 |
| COVPSU-00460 | female | 54 | S: L306 | Alive | Narathiwat | 1/11/2021 |
| COVPSU-00461 | male | 58 | S: F306 | Alive | Narathiwat | 1/11/2021 |
| COVPSU-00462 | male | 63 | S: F306 | Alive | Narathiwat | 1/11/2021 |
| COVPSU-00463 | male | 20 | S: L306 | Alive | Narathiwat | 1/11/2021 |
| COVPSU-00464 | male | 32 | S: F306 | Alive | Narathiwat | 1/11/2021 |
| COVPSU-00465 | female | 38 | S: F306 | Alive | Phatthalung | 1/11/2021 |
| COVPSU-00468 | male | 72 | S: F306 | Alive | Narathiwat | 1/11/2021 |
| COVPSU-00469 | female | 54 | S: F306 | Alive | Narathiwat | 1/11/2021 |
| COVPSU-00470 | female | 34 | S: F306 | Alive | Narathiwat | 3/11/2021 |
| COVPSU-00471 | female | 18 | S: F306 | Alive | Narathiwat | 3/11/2021 |
| COVPSU-00476 | male | 19 | S: F306 | Alive | Narathiwat | 5/11/2021 |
| COVPSU-00477 | female | 48 | S: F306 | Alive | Narathiwat | 5/11/2021 |
| COVPSU-00478 | male | 30 | S: F306 | Alive | Narathiwat | 3/11/2021 |
| COVPSU-00480 | female | 87 | S: F306 | Alive | Narathiwat | 8/11/2021 |
| COVPSU-00482 | male | 21 | S: F306 | Alive | Narathiwat | 8/11/2021 |
| COVPSU-00483 | female | 47 | S: L306 | Alive | Narathiwat | 8/11/2021 |
| COVPSU-00484 | female | 64 | S: F306 | Alive | Narathiwat | 8/11/2021 |
| COVPSU-00485 | male | 63 | S: F306 | Alive | Narathiwat | 8/11/2021 |
| COVPSU-00486 | male | 56 | S: F306 | Alive | Narathiwat | 8/11/2021 |
| COVPSU-00487 | male | 30 | S: F306 | Alive | Narathiwat | 8/11/2021 |
| COVPSU-00489 | female | 36 | S: F306 | Alive | Narathiwat | 8/11/2021 |
| COVPSU-00490 | female | 54 | S: L306 | Alive | Narathiwat | 8/11/2021 |
| COVPSU-00491 | male | 55 | S: F306 | Alive | Narathiwat | 8/11/2021 |
| COVPSU-00492 | male | 28 | S: F306 | Alive | Narathiwat | 8/11/2021 |
| COVPSU-00493 | male | 21 | S: F306 | Alive | Narathiwat | 8/11/2021 |
| COVPSU-00494 | male | 21 | S: F306 | Alive | Narathiwat | 8/11/2021 |
| COVPSU-00495 | female | 33 | S: F306 | Alive | Narathiwat | 6/11/2021 |
| COVPSU-00496 | female | 25 | S: F306 | Alive | Narathiwat | 9/11/2021 |
| COVPSU-00497 | male | 23 | S: F306 | Alive | Narathiwat | 9/11/2021 |
| COVPSU-00498 | male | NA | S: F306 | Alive | Narathiwat | 9/11/2021 |
| COVPSU-00499 | female | 47 | S: F306 | Alive | Yala | 19/10/2021 |
| COVPSU-00500 | female | 37 | S: L306 | Alive | Yala | 18/10/2021 |
| COVPSU-00501 | female | 27 | S: L306 | Alive | Yala | 23/10/2021 |
| COVPSU-00509 | male | 29 | S: L306 | Alive | Yala | 21/10/2021 |
| COVPSU-00510 | female | 45 | S: F306 | Alive | Yala | 24/10/2021 |
| COVPSU-00511 | female | 23 | S: L306 | Alive | Yala | 24/10/2021 |
| COVPSU-00514 | female | 44 | S: F306 | Alive | Yala | 24/10/2021 |
| COVPSU-00515 | female | 62 | S: F306 | Alive | Yala | 25/10/2021 |
| COVPSU-00516 | female | 37 | S: F306 | Alive | Yala | 25/10/2021 |
| COVPSU-00517 | male | 37 | S: L306 | Death | Yala | 14/10/2021 |
| COVPSU-00518 | female | 56 | S: F306 | Alive | Narathiwat | 27/10/2021 |
| COVPSU-00519 | male | 38 | S: F306 | Alive | Narathiwat | 25/10/2021 |
| COVPSU-00520 | female | 18 | S: L306 | Alive | Pattani | 27/10/2021 |
| COVPSU-00522 | female | 53 | S: F306 | Alive | Pattani | 26/10/2021 |
| COVPSU-00523 | male | NA | S: F306 | Alive | Pattani | 27/10/2021 |
| COVPSU-00525 | male | 48 | S: F306 | Alive | Songkhla | 26/10/2021 |
| COVPSU-00527 | female | 62 | S: F306 | Alive | Songkhla | 26/10/2021 |
| COVPSU-00528 | female | 43 | S: F306 | Alive | Songkhla | 26/10/2021 |
| COVPSU-00529 | female | 29 | S: F306 | Alive | Songkhla | 26/10/2021 |
| COVPSU-00530 | female | 45 | S: L306 | Alive | Songkhla | 23/10/2021 |
| COVPSU-00532 | female | 89 | S: L306 | Death | Yala | 27/10/2021 |
| COVPSU-00533 | female | 44 | S: L306 | Alive | Yala | 24/10/2021 |
| COVPSU-00534 | female | 33 | S: F306 | Alive | Narathiwat | 27/10/2021 |
| COVPSU-00535 | female | 40 | S: F306 | Alive | Yala | 31/10/2021 |
| COVPSU-00536 | female | 60 | S: L306 | Alive | Yala | 31/10/2021 |
| COVPSU-00537 | female | 60 | S: L306 | Alive | Narathiwat | 29/10/2021 |
| COVPSU-00538 | female | 52 | S: L306 | Alive | Narathiwat | 30/10/2021 |
| COVPSU-00539 | female | NA | S: F306 | Alive | Narathiwat | 29/10/2021 |
| COVPSU-00540 | male | NA | S: F306 | Alive | Narathiwat | 29/10/2021 |
| COVPSU-00541 | female | 92 | S: L306 | Death | Yala | 26/10/2021 |
| COVPSU-00543 | male | 80 | S: L306 | Alive | Yala | 28/10/2021 |
| COVPSU-00544 | male | 59 | S: F306 | Alive | Yala | 29/10/2021 |
| COVPSU-00545 | female | 25 | S: F306 | Alive | Yala | 31/10/2021 |
| COVPSU-00547 | female | 101 | S: L306 | Death | Narathiwat | 17/10/2021 |
| COVPSU-00548 | female | 100 | S: F306 | Alive | Narathiwat | 19/10/2021 |
| COVPSU-00551 | male | 70 | S: L306 | Death | Narathiwat | 24/10/2021 |
| COVPSU-00553 | female | 85 | S: F306 | Alive | Narathiwat | 26/10/2021 |
| COVPSU-00555 | male | 53 | S: L306 | Death | Narathiwat | 28/10/2021 |
| COVPSU-00557 | male | 27 | S: F306 | Alive | Narathiwat | 31/10/2021 |
| COVPSU-00559 | female | 58 | S: L306 | Alive | Songkhla | 2/11/2021 |
| COVPSU-00564 | female | 85 | S: F306 | Alive | Songkhla | 2/11/2021 |
| COVPSU-00572 | male | 30 | S: F306 | Alive | Songkhla | 2/11/2021 |
| COVPSU-00573 | female | NA | S: L306 | Alive | Songkhla | 2/11/2021 |
| COVPSU-00574 | female | NA | S: F306 | Alive | Songkhla | 2/11/2021 |
| COVPSU-00575 | female | 27 | S: L306 | Alive | Songkhla | 28/10/2021 |
| COVPSU-00578 | female | 5 | S: F306 | Alive | Songkhla | 3/11/2021 |
| COVPSU-00580 | male | 21 | S: L306 | Alive | Songkhla | 3/11/2021 |
| COVPSU-00581 | male | 48 | S: F306 | Alive | Narathiwat | 4/11/2021 |
| COVPSU-00582 | male | 49 | S: L306 | Alive | Narathiwat | 4/11/2021 |
| COVPSU-00583 | female | 29 | S: F306 | Alive | Narathiwat | 4/11/2021 |
| COVPSU-00584 | female | NA | S: L306 | Alive | Pattani | 3/11/2021 |
| COVPSU-00585 | female | NA | S: L306 | Alive | Pattani | 3/11/2021 |
| COVPSU-00586 | female | NA | S: F306 | Alive | Pattani | 3/11/2021 |
| COVPSU-00588 | female | 46 | S: L306 | Alive | Pattani | 1/11/2021 |
| COVPSU-00590 | female | 53 | S: F306 | Alive | Pattani | 3/11/2021 |
| COVPSU-00592 | female | 78 | S: L306 | Death | Yala | 31/10/2021 |
| COVPSU-00593 | male | 89 | S: L306 | Death | Yala | 17/10/2021 |
| COVPSU-00594 | female | 44 | S: L306 | Death | Yala | 4/11/2021 |
| COVPSU-00595 | female | 77 | S: L306 | Alive | Narathiwat | 2/11/2021 |
| COVPSU-00597 | female | 61 | S: L306 | Alive | Narathiwat | 4/11/2021 |
| COVPSU-00598 | male | 62 | S: F306 | Alive | Narathiwat | 4/11/2021 |
| COVPSU-00600 | male | 26 | S: F306 | Alive | Songkhla | 7/11/2021 |
| COVPSU-00601 | female | 15 | S: F306 | Alive | Songkhla | 7/11/2021 |
| COVPSU-00602 | female | 45 | S: L306 | Alive | Narathiwat | 4/11/2021 |
| COVPSU-00603 | male | 31 | S: F306 | Alive | Narathiwat | 6/11/2021 |
| COVPSU-00604 | female | 19 | S: F306 | Alive | Narathiwat | 6/11/2021 |
| COVPSU-00605 | female | 53 | S: F306 | Alive | Narathiwat | 6/11/2021 |
| COVPSU-00607 | female | 22 | S: L306 | Alive | Yala | 5/11/2021 |
| COVPSU-00608 | female | 42 | S: L306 | Alive | Yala | 5/11/2021 |
| COVPSU-00609 | female | 38 | S: L306 | Alive | Yala | 5/11/2021 |
| COVPSU-00610 | female | 33 | S: F306 | Alive | Yala | 5/11/2021 |
| COVPSU-00611 | female | 36 | S: L306 | Alive | Narathiwat | 5/11/2021 |
| COVPSU-00614 | male | 30 | S: F306 | Alive | Yala | 5/11/2021 |
| COVPSU-00615 | male | 28 | S: F306 | Alive | Yala | 5/11/2021 |
| COVPSU-00616 | female | NA | S: L306 | Alive | Yala | 5/11/2021 |
| COVPSU-00617 | female | 29 | S: L306 | Alive | Yala | 5/11/2021 |
| COVPSU-00618 | male | NA | S: F306 | Alive | Pattani | 10/11/2021 |
| COVPSU-00619 | male | 36 | S: L306 | Alive | Narathiwat | 9/11/2021 |
| COVPSU-00624 | female | 25 | S: L306 | Alive | Songkhla | 7/11/2021 |
| COVPSU-00625 | male | NA | S: F306 | Alive | Pattani | 2/11/2021 |
| COVPSU-00626 | female | 34 | S: F306 | Alive | Pattani | 12/11/2021 |
| COVPSU-00627 | female | 23 | S: F306 | Alive | Pattani | 12/11/2021 |
| COVPSU-00628 | female | 36 | S: F306 | Alive | Pattani | 12/11/2021 |
| COVPSU-00629 | male | 60 | S: F306 | Alive | Pattani | 12/11/2021 |
| COVPSU-00630 | male | 55 | S: L306 | Alive | Pattani | 12/11/2021 |
| COVPSU-00632 | female | 25 | S: L306 | Alive | Songkhla | 8/11/2021 |
| COVPSU-00633 | female | 29 | S: F306 | Alive | Narathiwat | 10/11/2021 |
| COVPSU-00634 | female | 58 | S: L306 | Alive | Narathiwat | 9/11/2021 |
| COVPSU-00635 | female | 32 | S: L306 | Alive | Yala | 14/11/2021 |
| COVPSU-00636 | female | 26 | S: F306 | Alive | Yala | 14/11/2021 |
| COVPSU-00637 | male | 54 | S: L306 | Alive | Yala | 14/11/2021 |
| COVPSU-00638 | male | NA | S: F306 | Alive | Songkhla | 15/11/2021 |
| COVPSU-00639 | female | NA | S: F306 | Alive | Songkhla | 15/11/2021 |
| COVPSU-00640 | male | NA | S: F306 | Alive | Songkhla | 15/11/2021 |
| COVPSU-00641 | male | NA | S: F306 | Alive | Songkhla | 15/11/2021 |
| COVPSU-00642 | male | NA | S: F306 | Alive | Songkhla | 15/11/2021 |
| COVPSU-00643 | male | NA | S: F306 | Alive | Songkhla | 15/11/2021 |
| COVPSU-00644 | male | NA | S: F306 | Alive | Songkhla | 15/11/2021 |
| COVPSU-00645 | male | NA | S: F306 | Alive | Songkhla | 15/11/2021 |
| COVPSU-00646 | female | 59 | S: L306 | Alive | Narathiwat | 10/11/2021 |
| COVPSU-00647 | female | 72 | S: F306 | Alive | Narathiwat | 12/11/2021 |
| COVPSU-00648 | male | 56 | S: L306 | Alive | Narathiwat | 12/11/2021 |
| COVPSU-00649 | female | 83 | S: L306 | Alive | Narathiwat | 13/11/2021 |
| COVPSU-00650 | female | 37 | S: L306 | Alive | Narathiwat | 13/11/2021 |
| COVPSU-00651 | male | 10 | S: F306 | Alive | Narathiwat | 14/11/2021 |
| COVPSU-00652 | female | 40 | S: L306 | Alive | Narathiwat | 9/11/2021 |
| COVPSU-00653 | female | 11 | S: L306 | Alive | Narathiwat | 12/11/2021 |
| COVPSU-00654 | male | 59 | S: L306 | Alive | Narathiwat | 10/11/2021 |
| COVPSU-00655 | male | 30 | S: F306 | Alive | Narathiwat | 10/11/2021 |
| COVPSU-00657 | male | 63 | S: L306 | Death | Narathiwat | 7/11/2021 |
| COVPSU-00658 | female | 41 | S: F306 | Alive | Narathiwat | 11/11/2021 |
| COVPSU-00659 | male | 57 | S: F306 | Alive | Narathiwat | 11/11/2021 |
| COVPSU-00661 | male | 70 | S: L306 | Death | Narathiwat | 13/11/2021 |
| COVPSU-00665 | male | 63 | S: F306 | Alive | Narathiwat | 12/11/2021 |
| COVPSU-00666 | male | 66 | S: F306 | Alive | Narathiwat | 15/11/2021 |
| COVPSU-00667 | male | 34 | S: F306 | Alive | Narathiwat | 10/11/2021 |
| COVPSU-00668 | female | 50 | S: F306 | Alive | Narathiwat | 10/11/2021 |
| COVPSU-00669 | female | 71 | S: L306 | Alive | Narathiwat | 10/11/2021 |
| COVPSU-00670 | male | 27 | S: F306 | Alive | Narathiwat | 10/11/2021 |
| COVPSU-00671 | female | 3 | S: F306 | Alive | Narathiwat | 10/11/2021 |
| COVPSU-00674 | female | 67 | S: L306 | Alive | Narathiwat | 11/11/2021 |
| COVPSU-00675 | male | 51 | S: F306 | Alive | Narathiwat | 11/11/2021 |
| COVPSU-00676 | female | 22 | S: F306 | Alive | Narathiwat | 11/11/2021 |
| COVPSU-00677 | female | 18 | S: L306 | Alive | Narathiwat | 12/11/2021 |
| COVPSU-00678 | male | 39 | S: F306 | Alive | Narathiwat | 12/11/2021 |
| COVPSU-00680 | female | 26 | S: F306 | Alive | Narathiwat | 12/11/2021 |
| COVPSU-00681 | male | 54 | S: F306 | Alive | Narathiwat | 12/11/2021 |
| COVPSU-00682 | female | 46 | S: F306 | Alive | Narathiwat | 12/11/2021 |
| COVPSU-00683 | female | 31 | S: F306 | Alive | Narathiwat | 12/11/2021 |
| COVPSU-00684 | female | 60 | S: F306 | Alive | Narathiwat | 12/11/2021 |
| COVPSU-00685 | male | 80 | S: F306 | Alive | Narathiwat | 12/11/2021 |
| COVPSU-00686 | female | 25 | S: F306 | Alive | Narathiwat | 14/11/2021 |
| COVPSU-00689 | female | NA | S: L306 | Alive | Songkhla | 18/11/2021 |
| COVPSU-00692 | female | 83 | S: F306 | Alive | Songkhla | 18/11/2021 |
| COVPSU-00693 | female | 25 | S: F306 | Alive | Yala | 17/11/2021 |
| COVPSU-00694 | female | NA | S: L306 | Alive | Yala | 17/11/2021 |
| COVPSU-00695 | female | NA | S: L306 | Alive | Yala | 17/11/2021 |
| COVPSU-00696 | female | NA | S: F306 | Alive | Yala | 17/11/2021 |
| COVPSU-00698 | male | NA | S: L306 | Alive | Yala | 17/11/2021 |
| COVPSU-00699 | male | 43 | S: L306 | Alive | Pattani | 17/11/2021 |
| COVPSU-00700 | male | 35 | S: F306 | Alive | Pattani | 17/11/2021 |
| COVPSU-00701 | male | 21 | S: F306 | Alive | Pattani | 17/11/2021 |
| COVPSU-00702 | male | 21 | S: F306 | Alive | Pattani | 17/11/2021 |
| COVPSU-00704 | male | 44 | S: L306 | Alive | Pattani | 17/11/2021 |
| COVPSU-00705 | male | 28 | S: L306 | Alive | Pattani | 17/11/2021 |
| COVPSU-00706 | female | 7 | S: L306 | Alive | Pattani | 17/11/2021 |
| COVPSU-00707 | female | 24 | S: F306 | Alive | Yala | 7/11/2021 |
| COVPSU-00708 | male | 65 | S: F306 | Alive | Yala | 15/11/2021 |
| COVPSU-00709 | male | 62 | S: F306 | Alive | Yala | 15/11/2021 |
| COVPSU-00711 | male | 53 | S: F306 | Alive | Yala | 15/11/2021 |
| COVPSU-00712 | male | 51 | S: F306 | Alive | Yala | 15/11/2021 |
| COVPSU-00713 | male | 25 | S: F306 | Alive | Yala | 15/11/2021 |
| COVPSU-00714 | female | 26 | S: F306 | Alive | Yala | 15/11/2021 |
| COVPSU-00715 | male | 44 | S: F306 | Alive | Yala | 15/11/2021 |
| COVPSU-00716 | male | 56 | S: F306 | Alive | Yala | 15/11/2021 |
| COVPSU-00717 | male | 58 | S: F306 | Alive | Yala | 15/11/2021 |
| COVPSU-00718 | male | 27 | S: F306 | Alive | Yala | 15/11/2021 |
| COVPSU-00719 | male | 57 | S: F306 | Alive | Yala | 15/11/2021 |
| COVPSU-00720 | female | 38 | S: F306 | Alive | Yala | 15/11/2021 |
| COVPSU-00721 | male | 57 | S: F306 | Alive | Yala | 15/11/2021 |
| COVPSU-00722 | male | 47 | S: F306 | Alive | Yala | 15/11/2021 |
| COVPSU-00723 | male | 26 | S: F306 | Alive | Yala | 15/11/2021 |
| COVPSU-00724 | male | 35 | S: F306 | Alive | Yala | 15/11/2021 |
| COVPSU-00725 | male | 41 | S: F306 | Alive | Yala | 15/11/2021 |
| COVPSU-00726 | male | 51 | S: F306 | Alive | Yala | 15/11/2021 |
| COVPSU-00727 | male | 40 | S: F306 | Alive | Yala | 15/11/2021 |
| COVPSU-00728 | male | 34 | S: F306 | Alive | Yala | 15/11/2021 |
| COVPSU-00729 | female | 21 | S: F306 | Alive | Yala | 15/11/2021 |
| COVPSU-00730 | female | 34 | S: F306 | Alive | Yala | 15/11/2021 |
| COVPSU-00731 | female | 31 | S: F306 | Alive | Yala | 15/11/2021 |
| COVPSU-00732 | male | NA | S: F306 | Alive | Songkhla | 8/11/2021 |
| COVPSU-00733 | male | NA | S: F306 | Alive | Songkhla | 5/11/2021 |
| COVPSU-00734 | male | NA | S: F306 | Alive | Songkhla | 5/11/2021 |
| COVPSU-00735 | female | NA | S: L306 | Alive | Songkhla | 15/11/2021 |
| COVPSU-00736 | female | NA | S: L306 | Alive | Songkhla | 15/11/2021 |
| COVPSU-00737 | female | NA | S: L306 | Alive | Songkhla | 13/11/2021 |
| COVPSU-00738 | female | NA | S: F306 | Alive | Songkhla | 12/11/2021 |
| COVPSU-00740 | male | 54 | S: F306 | Alive | Pattani | 15/11/2021 |
| COVPSU-00741 | female | 65 | S: F306 | Alive | Pattani | 15/11/2021 |
| COVPSU-00742 | male | 55 | S: F306 | Alive | Pattani | 15/11/2021 |
| COVPSU-00743 | male | 67 | S: F306 | Alive | Pattani | 15/11/2021 |
| COVPSU-00744 | female | 86 | S: L306 | Alive | Yala | 7/11/2021 |
| COVPSU-00745 | male | 61 | S: L306 | Alive | Yala | 8/11/2021 |
| COVPSU-00746 | female | 34 | S: L306 | Alive | Pattani | 15/11/2021 |
| COVPSU-00747 | female | 37 | S: F306 | Alive | Pattani | 15/11/2021 |
| COVPSU-00748 | female | 36 | S: L306 | Alive | Pattani | 15/11/2021 |
| COVPSU-00750 | female | 24 | S: F306 | Alive | Pattani | 15/11/2021 |
| COVPSU-00751 | female | 66 | S: L306 | Alive | Pattani | 15/11/2021 |
| COVPSU-00752 | female | 30 | S: F306 | Alive | Pattani | 15/11/2021 |
| COVPSU-00753 | male | 33 | S: L306 | Alive | Pattani | 15/11/2021 |
| COVPSU-00754 | male | 53 | S: F306 | Alive | Pattani | 15/11/2021 |
| COVPSU-00757 | female | 41 | S: L306 | Alive | Yala | 15/11/2021 |
| COVPSU-00758 | male | 13 | S: F306 | Alive | Yala | 21/11/2021 |
| COVPSU-00759 | female | 71 | S: F306 | Alive | Yala | 21/11/2021 |
| COVPSU-00760 | female | 57 | S: L306 | Alive | Yala | 21/11/2021 |
| COVPSU-00761 | male | 57 | S: F306 | Alive | Yala | 17/11/2021 |
| COVPSU-00762 | female | 35 | S: L306 | Alive | Yala | 17/11/2021 |
| COVPSU-00763 | female | 61 | S: F306 | Alive | Yala | 18/11/2021 |
| COVPSU-00764 | female | 58 | S: F306 | Alive | Yala | 18/11/2021 |
| COVPSU-00765 | male | 13 | S: L306 | Alive | Narathiwat | 16/11/2021 |
| COVPSU-00766 | female | 55 | S: F306 | Alive | Narathiwat | 16/11/2021 |
| COVPSU-00767 | female | 58 | S: F306 | Alive | Narathiwat | 16/11/2021 |
| COVPSU-00768 | male | 55 | S: L306 | Alive | Narathiwat | 16/11/2021 |
| COVPSU-00769 | male | 41 | S: L306 | Alive | Narathiwat | 16/11/2021 |
| COVPSU-00772 | female | 54 | S: L306 | Alive | Narathiwat | 18/11/2021 |
| COVPSU-00774 | female | 35 | S: L306 | Alive | Narathiwat | 19/11/2021 |
| COVPSU-00775 | female | 82 | S: F306 | Alive | Narathiwat | 20/11/2021 |
| COVPSU-00776 | female | 51 | S: L306 | Alive | Narathiwat | 20/11/2021 |
| COVPSU-00777 | female | NA | S: F306 | Alive | Narathiwat | 21/11/2021 |
| COVPSU-00778 | female | 19 | S: L306 | Alive | Narathiwat | 21/11/2021 |
| COVPSU-00780 | male | 62 | S: L306 | Alive | Narathiwat | 16/11/2021 |
| COVPSU-00781 | male | NA | S: L306 | Alive | Narathiwat | 16/11/2021 |
| COVPSU-00782 | male | 52 | S: F306 | Alive | Narathiwat | 19/11/2021 |
| COVPSU-00783 | female | 29 | S: L306 | Alive | Narathiwat | 15/11/2021 |
| COVPSU-00785 | female | 38 | S: F306 | Alive | Narathiwat | 17/11/2021 |
| COVPSU-00786 | female | 41 | S: L306 | Alive | Narathiwat | 18/11/2021 |
| COVPSU-00787 | female | 5 | S: F306 | Alive | Narathiwat | 18/11/2021 |
| COVPSU-00788 | female | 100 | S: L306 | Alive | Narathiwat | 18/11/2021 |
| COVPSU-00789 | female | 36 | S: F306 | Alive | Narathiwat | 19/11/2021 |
| COVPSU-00791 | female | 59 | S: F306 | Alive | Narathiwat | 15/11/2021 |
| COVPSU-00792 | female | 30 | S: F306 | Alive | Narathiwat | 15/11/2021 |
| COVPSU-00794 | male | 3 | S: F306 | Alive | Narathiwat | 15/11/2021 |
| COVPSU-00795 | female | 11 | S: L306 | Alive | Narathiwat | 15/11/2021 |
| COVPSU-00796 | female | 44 | S: F306 | Alive | Narathiwat | 16/11/2021 |
| COVPSU-00797 | female | 33 | S: L306 | Alive | Narathiwat | 17/11/2021 |
| COVPSU-00798 | female | 34 | S: L306 | Alive | Narathiwat | 17/11/2021 |
| COVPSU-00800 | female | 63 | S: L306 | Alive | Narathiwat | 17/11/2021 |
| COVPSU-00801 | female | 41 | S: L306 | Alive | Narathiwat | 17/11/2021 |
| COVPSU-00807 | female | 33 | S: L306 | Alive | Songkhla | 17/11/2021 |
| COVPSU-00808 | female | 27 | S: F306 | Alive | Songkhla | 17/11/2021 |
| COVPSU-00809 | female | 34 | S: L306 | Alive | Songkhla | 18/11/2021 |
| COVPSU-00810 | female | 61 | S: F306 | Alive | Songkhla | 12/11/2021 |
| COVPSU-00811 | female | 82 | S: F306 | Alive | Songkhla | 21/11/2021 |
| COVPSU-00815 | male | 21 | S: F306 | Alive | Songkhla | 22/11/2021 |
| COVPSU-00816 | male | 23 | S: F306 | Alive | Songkhla | 22/11/2021 |
| COVPSU-00821 | male | 44 | S: F306 | Alive | Pattani | 18/11/2021 |
| COVPSU-00822 | female | 67 | S: F306 | Alive | Pattani | 18/11/2021 |
| COVPSU-00823 | female | 34 | S: F306 | Alive | Pattani | 18/11/2021 |
| COVPSU-00824 | female | 36 | S: F306 | Alive | Pattani | 19/11/2021 |
| COVPSU-00825 | male | 33 | S: F306 | Alive | Pattani | 19/11/2021 |
| COVPSU-00826 | female | 66 | S: F306 | Alive | Pattani | 21/11/2021 |
| COVPSU-00827 | female | 35 | S: F306 | Alive | Pattani | 21/11/2021 |
| COVPSU-00829 | male | NA | S: L306 | Alive | Songkhla | 27/11/2021 |
| COVPSU-00832 | male | NA | S: F306 | Alive | Songkhla | 27/11/2021 |
| COVPSU-00834 | male | NA | S: F306 | Alive | Songkhla | 27/11/2021 |
| COVPSU-00835 | male | 39 | S: F306 | Alive | Songkhla | 27/11/2021 |
| COVPSU-00836 | male | 32 | S: L306 | Alive | Songkhla | 27/11/2021 |
| COVPSU-00839 | female | 7 | S: F306 | Alive | Songkhla | 28/11/2021 |
| COVPSU-00840 | male | 12 | S: L306 | Alive | Songkhla | 28/11/2021 |
| COVPSU-00841 | male | 23 | S: F306 | Alive | Songkhla | 27/11/2021 |
| COVPSU-00842 | female | 26 | S: F306 | Alive | Songkhla | 27/11/2021 |
| COVPSU-00845 | female | 27 | S: L306 | Alive | Songkhla | 25/11/2021 |
| COVPSU-00846 | male | NA | S: L306 | Alive | Songkhla | 25/11/2021 |
| COVPSU-00847 | male | 62 | S: F306 | Alive | Songkhla | 27/11/2021 |
| COVPSU-00848 | female | 15 | S: F306 | Alive | Songkhla | 26/11/2021 |
| COVPSU-00849 | female | NA | S: F306 | Alive | Songkhla | 26/11/2021 |
| COVPSU-00851 | female | 34 | S: F306 | Alive | Yala | 26/11/2021 |
| COVPSU-00852 | male | 21 | S: L306 | Alive | Yala | 25/11/2021 |
| COVPSU-00853 | female | 33 | S: L306 | Alive | Yala | 25/11/2021 |
| COVPSU-00854 | female | 29 | S: L306 | Alive | Yala | 25/11/2021 |
| COVPSU-00855 | female | 43 | S: F306 | Alive | Yala | 27/11/2021 |
| COVPSU-00856 | female | 57 | S: L306 | Alive | Narathiwat | 22/11/2021 |
| COVPSU-00857 | male | 2 | S: F306 | Alive | Narathiwat | 22/11/2021 |
| COVPSU-00858 | female | 37 | S: F306 | Alive | Narathiwat | 22/11/2021 |
| COVPSU-00859 | female | 32 | S: F306 | Alive | Narathiwat | 22/11/2021 |
| COVPSU-00860 | female | 34 | S: F306 | Alive | Narathiwat | 22/11/2021 |
| COVPSU-00863 | female | 55 | S: F306 | Alive | Narathiwat | 22/11/2021 |
| COVPSU-00864 | female | 22 | S: L306 | Alive | Narathiwat | 23/11/2021 |
| COVPSU-00865 | female | 22 | S: L306 | Alive | Narathiwat | 23/11/2021 |
| COVPSU-00866 | male | 71 | S: L306 | Alive | Narathiwat | 23/11/2021 |
| COVPSU-00867 | female | 35 | S: L306 | Alive | Narathiwat | 24/11/2021 |
| COVPSU-00868 | male | NA | S: F306 | Alive | Narathiwat | 25/11/2021 |
| COVPSU-00869 | male | 43 | S: L306 | Alive | Narathiwat | 25/11/2021 |
| COVPSU-00870 | male | 23 | S: F306 | Alive | Narathiwat | 26/11/2021 |
| COVPSU-00871 | male | 2 | S: F306 | Alive | Narathiwat | 26/11/2021 |
| COVPSU-00873 | female | 28 | S: L306 | Alive | Narathiwat | 26/11/2021 |
| COVPSU-00874 | female | 26 | S: L306 | Alive | Narathiwat | 26/11/2021 |
| COVPSU-00875 | female | 65 | S: L306 | Alive | Narathiwat | 22/11/2021 |
| COVPSU-00876 | male | 1 | S: F306 | Alive | Narathiwat | 22/11/2021 |
| COVPSU-00877 | male | 67 | S: F306 | Alive | Narathiwat | 22/11/2021 |
| COVPSU-00878 | male | 75 | S: F306 | Alive | Narathiwat | 22/11/2021 |
| COVPSU-00879 | male | 3 | S: F306 | Alive | Narathiwat | 22/11/2021 |
| COVPSU-00880 | male | 46 | S: L306 | Alive | Narathiwat | 23/11/2021 |
| COVPSU-00881 | female | 52 | S: L306 | Alive | Narathiwat | 24/11/2021 |
| COVPSU-00884 | female | 85 | S: L306 | Alive | Narathiwat | 26/11/2021 |
| COVPSU-00885 | male | 11 | S: F306 | Alive | Narathiwat | 27/11/2021 |
| COVPSU-00886 | female | 37 | S: F306 | Alive | Narathiwat | 27/11/2021 |
| COVPSU-00887 | female | 34 | S: L306 | Alive | Narathiwat | 27/11/2021 |
| COVPSU-00888 | male | 47 | S: F306 | Alive | Narathiwat | 22/11/2021 |
| COVPSU-00889 | female | 63 | S: L306 | Alive | Narathiwat | 22/11/2021 |
| COVPSU-00890 | female | 40 | S: F306 | Alive | Narathiwat | 23/11/2021 |
| COVPSU-00891 | male | 46 | S: F306 | Alive | Narathiwat | 23/11/2021 |
| COVPSU-00892 | female | 54 | S: F306 | Alive | Narathiwat | 23/11/2021 |
| COVPSU-00894 | female | 76 | S: L306 | Death | Narathiwat | 27/11/2021 |
| COVPSU-00895 | female | 54 | S: L306 | Alive | Songkhla | 26/11/2021 |
| COVPSU-00897 | male | 5 | S: F306 | Alive | Songkhla | 27/11/2021 |
| COVPSU-00898 | female | 41 | S: F306 | Alive | Songkhla | 29/11/2021 |
| COVPSU-00899 | female | 41 | S: F306 | Alive | Songkhla | 29/11/2021 |
| COVPSU-00900 | female | 26 | S: F306 | Alive | Songkhla | 30/11/2021 |
| COVPSU-00901 | male | 24 | S: F306 | Alive | Songkhla | 30/11/2021 |
| COVPSU-00902 | female | 64 | S: L306 | Alive | Yala | 1/12/2021 |
| COVPSU-00903 | female | 36 | S: L306 | Alive | Yala | 1/12/2021 |
| COVPSU-00904 | female | 30 | S: L306 | Alive | Yala | 1/12/2021 |
| COVPSU-00905 | male | NA | S: L306 | Alive | Yala | 1/12/2021 |
| COVPSU-00906 | female | 35 | S: L306 | Alive | Yala | 1/12/2021 |
| COVPSU-00907 | male | 12 | S: L306 | Alive | Yala | 1/12/2021 |
| COVPSU-00908 | female | 70 | S: F306 | Alive | Yala | 1/12/2021 |
| COVPSU-00909 | female | 55 | S: F306 | Alive | Yala | 1/12/2021 |
| COVPSU-00910 | female | NA | S: F306 | Alive | Yala | 1/12/2021 |
| COVPSU-00911 | female | NA | S: F306 | Alive | Yala | 1/12/2021 |
| COVPSU-00912 | male | NA | S: F306 | Alive | Yala | 1/12/2021 |
| COVPSU-00913 | female | 37 | S: F306 | Alive | Songkhla | 1/12/2021 |
| COVPSU-00914 | male | 41 | S: F306 | Alive | Songkhla | 30/11/2021 |
| COVPSU-00915 | male | 21 | S: F306 | Alive | Songkhla | 30/11/2021 |
| COVPSU-00916 | male | NA | S: L306 | Alive | Songkhla | 25/11/2021 |
| COVPSU-00918 | male | NA | S: L306 | Alive | Songkhla | 27/11/2021 |
| COVPSU-00921 | male | NA | S: L306 | Alive | Songkhla | 30/11/2021 |
| COVPSU-00922 | female | NA | S: F306 | Alive | Songkhla | 30/11/2021 |
| COVPSU-00923 | female | NA | S: F306 | Alive | Songkhla | 30/11/2021 |
| COVPSU-00924 | male | NA | S: L306 | Alive | Songkhla | 30/11/2021 |
| COVPSU-00925 | female | NA | S: F306 | Alive | Songkhla | 30/11/2021 |
| COVPSU-00926 | female | NA | S: L306 | Alive | Songkhla | 30/11/2021 |
| COVPSU-00927 | female | NA | S: F306 | Alive | Songkhla | 30/11/2021 |
| COVPSU-00928 | female | NA | S: F306 | Alive | Songkhla | 30/11/2021 |
| COVPSU-00929 | female | NA | S: L306 | Alive | Songkhla | 30/11/2021 |
| COVPSU-00930 | female | NA | S: L306 | Alive | Songkhla | 30/11/2021 |
| COVPSU-00932 | male | 9 | S: F306 | Alive | Narathiwat | 22/11/2021 |
| COVPSU-00933 | female | 22 | S: L306 | Alive | Narathiwat | 22/11/2021 |
| COVPSU-00934 | female | 58 | S: F306 | Alive | Narathiwat | 23/11/2021 |
| COVPSU-00935 | female | 25 | S: L306 | Alive | Narathiwat | 24/11/2021 |
| COVPSU-00936 | female | 58 | S: L306 | Alive | Narathiwat | 25/11/2021 |
| COVPSU-00937 | male | 23 | S: L306 | Alive | Narathiwat | 25/11/2021 |
| COVPSU-00938 | female | 27 | S: L306 | Alive | Narathiwat | 25/11/2021 |
| COVPSU-00939 | male | 36 | S: L306 | Alive | Narathiwat | 27/11/2021 |
| COVPSU-00944 | female | 35 | S: L306 | Alive | Yala | 29/11/2021 |
| COVPSU-00945 | female | 87 | S: F306 | Death | Yala | 19/11/2021 |
| COVPSU-00946 | male | 60 | S: F306 | Alive | Songkhla | 2/12/2021 |
| COVPSU-00947 | female | 29 | S: L306 | Alive | Songkhla | 2/12/2021 |
| COVPSU-00948 | male | 9 | S: L306 | Alive | Songkhla | 2/12/2021 |
| COVPSU-00949 | male | 27 | S: F306 | Alive | Songkhla | 2/12/2021 |
| COVPSU-00950 | female | 59 | S: F306 | Alive | Songkhla | 2/12/2021 |
| COVPSU-00952 | male | NA | S: F306 | Alive | Narathiwat | 8/11/2021 |
| COVPSU-00953 | female | NA | S: F306 | Alive | Narathiwat | 8/11/2021 |
| COVPSU-00955 | female | NA | S: F306 | Alive | Narathiwat | 12/11/2021 |
| COVPSU-00956 | male | NA | S: F306 | Alive | Narathiwat | 12/11/2021 |
| COVPSU-00959 | female | 5 | S: F306 | Alive | Songkhla | 3/12/2021 |
| COVPSU-00960 | male | 55 | S: L306 | Alive | Pattani | 25/11/2021 |
| COVPSU-00961 | male | 37 | S: F306 | Alive | Pattani | 26/11/2021 |
| COVPSU-00962 | male | 43 | S: L306 | Alive | Pattani | 28/11/2021 |
| COVPSU-00964 | female | 38 | S: F306 | Alive | Pattani | 30/11/2021 |
| COVPSU-00965 | male | 4 | S: F306 | Alive | Pattani | 30/11/2021 |
| COVPSU-00966 | male | 64 | S: F306 | Alive | Pattani | 1/12/2021 |
| COVPSU-00967 | female | 1 | S: L306 | Alive | Songkhla | 3/12/2021 |
| COVPSU-00969 | female | 40 | S: L306 | Alive | Songkhla | 3/12/2021 |
| COVPSU-00970 | female | NA | S: F306 | Alive | Songkhla | 3/12/2021 |
| COVPSU-00971 | female | NA | S: F306 | Alive | Songkhla | 3/12/2021 |
| COVPSU-00972 | male | 19 | S: F306 | Alive | Songkhla | 3/12/2021 |
| COVPSU-00973 | male | 39 | S: L306 | Alive | Songkhla | 3/12/2021 |
| COVPSU-00974 | female | 62 | S: L306 | Alive | Yala | 4/12/2021 |
| COVPSU-00975 | male | 34 | S: L306 | Alive | Yala | 5/12/2021 |
| COVPSU-00976 | male | 69 | S: L306 | Alive | Yala | 5/12/2021 |
| COVPSU-00983 | male | 28 | S: L306 | Alive | Songkhla | 3/12/2021 |
| COVPSU-00984 | male | 37 | S: L306 | Alive | Songkhla | 5/12/2021 |
| COVPSU-00985 | female | 26 | S: L306 | Alive | Songkhla | 5/12/2021 |
| COVPSU-00986 | male | 27 | S: F306 | Alive | Songkhla | 2/12/2021 |
| COVPSU-00987 | male | 28 | S: F306 | Alive | Songkhla | 1/12/2021 |
| COVPSU-00988 | male | 34 | S: L306 | Alive | Songkhla | 4/12/2021 |
| COVPSU-00989 | male | 46 | S: L306 | Alive | Songkhla | 4/12/2021 |
| COVPSU-00992 | male | 35 | S: L306 | Alive | Songkhla | 5/12/2021 |
| COVPSU-00995 | female | 28 | S: F306 | Alive | Songkhla | 1/12/2021 |
| COVPSU-00996 | female | 72 | S: F306 | Alive | Songkhla | 26/11/2021 |
| COVPSU-00998 | female | 74 | S: L306 | Death | Narathiwat | 29/11/2021 |
| COVPSU-01002 | female | 30 | S: L306 | Alive | Narathiwat | 2/12/2021 |
| COVPSU-01004 | female | 32 | S: F306 | Alive | Narathiwat | 30/11/2021 |
| COVPSU-01005 | male | 28 | S: F306 | Alive | Narathiwat | 30/11/2021 |
| COVPSU-01006 | female | 33 | S: L306 | Alive | Narathiwat | 3/12/2021 |
| COVPSU-01007 | male | 30 | S: F306 | Alive | Narathiwat | 2/12/2021 |
| COVPSU-01008 | female | 76 | S: L306 | Death | Narathiwat | 18/11/2021 |
| COVPSU-01009 | female | 35 | S: L306 | Alive | Narathiwat | 1/12/2021 |
| COVPSU-01010 | male | 56 | S: L306 | Alive | Narathiwat | 2/12/2021 |
| COVPSU-01011 | female | 42 | S: L306 | Alive | Narathiwat | 2/12/2021 |
| COVPSU-01012 | female | 56 | S: L306 | Alive | Yala | 2/12/2021 |
| COVPSU-01013 | male | 4 | S: F306 | Alive | Yala | 2/12/2021 |
| COVPSU-01014 | female | 27 | S: L306 | Alive | Yala | 3/12/2021 |
| COVPSU-01015 | female | 69 | S: F306 | Alive | Narathiwat | 29/11/2021 |
| COVPSU-01016 | female | 13 | S: L306 | Alive | Narathiwat | 29/11/2021 |
| COVPSU-01018 | male | 49 | S: F306 | Alive | Narathiwat | 1/12/2021 |
| COVPSU-01019 | female | 37 | S: L306 | Alive | Narathiwat | 3/12/2021 |
| COVPSU-01020 | female | 18 | S: F306 | Alive | Narathiwat | 3/12/2021 |
| COVPSU-01021 | male | 51 | S: F306 | Alive | Narathiwat | 3/12/2021 |
| COVPSU-01022 | female | 39 | S: F306 | Alive | Narathiwat | 4/12/2021 |
| COVPSU-01026 | female | 40 | S: F306 | Alive | Narathiwat | 2/12/2021 |
| COVPSU-01027 | male | 76 | S: F306 | Alive | Narathiwat | 3/12/2021 |
| COVPSU-01028 | male | 37 | S: F306 | Alive | Songkhla | 30/11/2021 |
| COVPSU-01029 | female | 24 | S: L306 | Alive | Songkhla | 2/12/2021 |
| COVPSU-01030 | male | 1 | S: L306 | Alive | Songkhla | 4/12/2021 |
| COVPSU-01031 | male | 50 | S: F306 | Alive | Songkhla | 6/12/2021 |
| COVPSU-01033 | female | 38 | S: L306 | Alive | Songkhla | 1/12/2021 |
| COVPSU-01034 | male | 1 | S: L306 | Alive | Songkhla | 3/12/2021 |
| COVPSU-01035 | female | 63 | S: F306 | Alive | Songkhla | 7/12/2021 |
| COVPSU-01036 | male | 6 | S: L306 | Alive | Songkhla | 8/12/2021 |
| COVPSU-01037 | male | 40 | S: F306 | Alive | Songkhla | 8/12/2021 |
| COVPSU-01043 | male | 11 | S: F306 | Alive | Songkhla | 9/12/2021 |
| COVPSU-01045 | male | 38 | S: F306 | Alive | Phatthalung | 8/12/2021 |
| COVPSU-01046 | female | 84 | S: F306 | Alive | Phatthalung | 8/12/2021 |
| COVPSU-01047 | female | 51 | S: F306 | Alive | Phatthalung | 8/12/2021 |
| COVPSU-01048 | male | 72 | S: L306 | Alive | Phatthalung | 9/12/2021 |
| COVPSU-01049 | female | 74 | S: F306 | Alive | Phatthalung | 9/12/2021 |
| COVPSU-01050 | male | 57 | S: F306 | Alive | Phatthalung | 9/12/2021 |
| COVPSU-01052 | male | 62 | S: F306 | Alive | Songkhla | 10/12/2021 |
| COVPSU-01053 | male | 67 | S: F306 | Alive | Songkhla | 10/12/2021 |
| COVPSU-01056 | female | 59 | S: L306 | Alive | Songkhla | 2/12/2021 |
| COVPSU-01057 | female | 65 | S: L306 | Alive | Songkhla | 2/12/2021 |
| COVPSU-01058 | male | 32 | S: L306 | Alive | Songkhla | 2/12/2021 |
| COVPSU-01059 | female | 10 | S: L306 | Alive | Songkhla | 2/12/2021 |
| COVPSU-01060 | female | 26 | S: F306 | Alive | Yala | 8/12/2021 |
| COVPSU-01061 | female | 8 | S: F306 | Alive | Yala | 9/12/2021 |
| COVPSU-01062 | female | 60 | S: F306 | Alive | Yala | 8/12/2021 |
| COVPSU-01063 | male | NA | S: F306 | Alive | Yala | 9/12/2021 |
| COVPSU-01064 | female | 41 | S: L306 | Alive | Yala | 11/12/2021 |
| COVPSU-01065 | male | 9 | S: L306 | Alive | Yala | 11/12/2021 |
| COVPSU-01066 | female | 70 | S: L306 | Alive | Yala | 12/12/2021 |
| COVPSU-01067 | female | 45 | S: L306 | Alive | Yala | 12/12/2021 |
| COVPSU-01068 | female | 71 | S: L306 | Death | Yala | 16/11/2021 |
| COVPSU-01069 | female | 80 | S: F306 | Death | Yala | 5/12/2021 |
| COVPSU-01072 | male | 22 | S: F306 | Alive | Narathiwat | 10/12/2021 |
| COVPSU-01073 | female | 71 | S: L306 | Alive | Narathiwat | 30/11/2021 |
| COVPSU-01075 | female | 67 | S: F306 | Alive | Narathiwat | 8/12/2021 |
| COVPSU-01077 | female | 25 | S: L306 | Alive | Narathiwat | 9/12/2021 |
| COVPSU-01078 | male | 20 | S: F306 | Alive | Narathiwat | 11/12/2021 |
| COVPSU-01080 | male | 18 | S: F306 | Alive | Narathiwat | 6/12/2021 |
| COVPSU-01081 | female | 23 | S: F306 | Alive | Narathiwat | 7/12/2021 |
| COVPSU-01082 | male | NA | S: F306 | Alive | Narathiwat | 8/12/2021 |
| COVPSU-01083 | male | 24 | S: F306 | Alive | Narathiwat | 9/12/2021 |
| COVPSU-01084 | male | 81 | S: F306 | Alive | Narathiwat | 9/12/2021 |
| COVPSU-01085 | male | 1 | S: F306 | Alive | Narathiwat | 9/12/2021 |
| COVPSU-01086 | male | NA | S: L306 | Alive | Narathiwat | 9/12/2021 |
| COVPSU-01087 | female | 100 | S: L306 | Alive | Narathiwat | 8/12/2021 |
| COVPSU-01089 | male | 50 | S: F306 | Alive | Narathiwat | 8/12/2021 |
| COVPSU-01091 | female | 15 | S: L306 | Alive | Narathiwat | 8/12/2021 |
| COVPSU-01092 | female | 51 | S: F306 | Alive | Narathiwat | 10/12/2021 |
| COVPSU-01093 | female | 74 | S: F306 | Alive | Narathiwat | 10/12/2021 |
| COVPSU-01094 | male | 15 | S: F306 | Alive | Narathiwat | 10/12/2021 |
| COVPSU-01095 | female | 31 | S: F306 | Alive | Narathiwat | 10/12/2021 |
| COVPSU-01096 | female | 73 | S: L306 | Alive | Narathiwat | 10/12/2021 |
| COVPSU-01098 | female | NA | S: F306 | Alive | Songkhla | 15/12/2021 |
| COVPSU-01101 | male | NA | S: L306 | Alive | Songkhla | 15/12/2021 |
| COVPSU-01104 | male | NA | S: F306 | Alive | Songkhla | 15/12/2021 |
| COVPSU-01105 | female | NA | S: F306 | Alive | Songkhla | 15/12/2021 |
| COVPSU-01107 | female | NA | S: L306 | Alive | Songkhla | 16/12/2021 |
| COVPSU-01108 | female | NA | S: F306 | Alive | Songkhla | 16/12/2021 |
| COVPSU-01110 | male | NA | S: L306 | Alive | Songkhla | 16/12/2021 |
| COVPSU-01112 | female | NA | S: F306 | Alive | Songkhla | 16/12/2021 |
| COVPSU-01113 | female | NA | S: F306 | Alive | Songkhla | 16/12/2021 |
| COVPSU-01115 | female | 47 | S: L306 | Alive | Songkhla | 13/12/2021 |
| COVPSU-01116 | female | 52 | S: F306 | Alive | Songkhla | 14/12/2021 |
| COVPSU-01117 | male | 47 | S: L306 | Alive | Songkhla | 14/12/2021 |
| COVPSU-01118 | female | NA | S: F306 | Alive | Songkhla | 15/12/2021 |
| COVPSU-01119 | male | 54 | S: F306 | Alive | Songkhla | 9/12/2021 |
| COVPSU-01120 | female | 20 | S: L306 | Alive | Songkhla | 12/12/2021 |
| COVPSU-01121 | male | 42 | S: L306 | Alive | Songkhla | 16/12/2021 |
| COVPSU-01126 | female | 53 | S: F306 | Alive | Songkhla | 20/12/2021 |
| COVPSU-01127 | female | 35 | S: F306 | Alive | Songkhla | 12/12/2021 |
| COVPSU-01128 | female | 3 | S: F306 | Alive | Songkhla | 24/12/2021 |
| COVPSU-01129 | female | 38 | S: F306 | Alive | Songkhla | 27/12/2021 |
| COVPSU-01130 | male | 63 | S: L306 | Alive | Songkhla | 24/12/2021 |
| COVPSU-01131 | female | 57 | S: F306 | Alive | Pattani | 2/12/2021 |
| COVPSU-01132 | male | 30 | S: F306 | Alive | Pattani | 3/12/2021 |
| COVPSU-01134 | female | 44 | S: F306 | Alive | Pattani | 5/12/2021 |
| COVPSU-01135 | female | 17 | S: F306 | Alive | Pattani | 6/12/2021 |
| COVPSU-01136 | female | 52 | S: F306 | Alive | Pattani | 9/12/2021 |
| COVPSU-01137 | female | 47 | S: F306 | Alive | Pattani | 10/12/2021 |
| COVPSU-01138 | female | 49 | S: F306 | Alive | Pattani | 12/12/2021 |
| COVPSU-01139 | female | 52 | S: F306 | Alive | Pattani | 13/12/2021 |
| COVPSU-01140 | male | 16 | S: F306 | Alive | Pattani | 15/12/2021 |
| COVPSU-01141 | male | NA | S: L306 | Alive | Songkhla | 13/12/2021 |
| COVPSU-01143 | male | NA | S: F306 | Alive | Songkhla | 15/12/2021 |
| COVPSU-01145 | female | NA | S: F306 | Alive | Songkhla | 15/12/2021 |
| COVPSU-01146 | male | NA | S: F306 | Alive | Songkhla | 15/12/2021 |
| COVPSU-01147 | male | NA | S: L306 | Alive | Songkhla | 16/12/2021 |
| COVPSU-01148 | female | NA | S: L306 | Alive | Songkhla | 15/12/2021 |
| COVPSU-01149 | male | NA | S: F306 | Alive | Songkhla | 15/12/2021 |
| COVPSU-01153 | male | 57 | S: F306 | Alive | Phatthalung | 11/12/2021 |
| COVPSU-01154 | male | 14 | S: F306 | Alive | Phatthalung | 12/12/2021 |
| COVPSU-01155 | female | 26 | S: F306 | Alive | Phatthalung | 12/12/2021 |
| COVPSU-01156 | male | 54 | S: F306 | Alive | Phatthalung | 12/12/2021 |
| COVPSU-01158 | female | 75 | S: F306 | Alive | Phatthalung | 13/12/2021 |
| COVPSU-01159 | female | 38 | S: F306 | Alive | Pattani | 13/12/2021 |
| COVPSU-01161 | female | 38 | S: F306 | Alive | Phatthalung | 14/12/2021 |
| COVPSU-01162 | male | 16 | S: L306 | Alive | Phatthalung | 14/12/2021 |
| COVPSU-01163 | female | 24 | S: L306 | Alive | Pattani | 14/12/2021 |
| COVPSU-01165 | female | 82 | S: L306 | Alive | Pattani | 15/12/2021 |
| COVPSU-01166 | male | 16 | S: L306 | Alive | Pattani | 15/12/2021 |
| COVPSU-01169 | male | 64 | S: L306 | Alive | Pattani | 16/12/2021 |
| COVPSU-01170 | female | 13 | S: L306 | Alive | Phatthalung | 16/12/2021 |
| COVPSU-01171 | male | 55 | S: F306 | Alive | Phatthalung | 16/12/2021 |
| COVPSU-01172 | female | 27 | S: L306 | Alive | Phatthalung | 16/12/2021 |
| COVPSU-01173 | male | 16 | S: L306 | Alive | Pattani | 17/12/2021 |
| COVPSU-01176 | female | 66 | S: L306 | Alive | Phatthalung | 17/12/2021 |
| COVPSU-01178 | female | 59 | S: F306 | Alive | Songkhla | 22/1/2022 |
| COVPSU-01179 | male | 53 | S: L306 | Alive | Songkhla | 17/12/2021 |
| COVPSU-01180 | male | 75 | S: F306 | Alive | Songkhla | 18/12/2021 |
| COVPSU-01191 | female | 67 | S: F306 | Alive | Pattani | 16/12/2021 |
| COVPSU-01192 | female | 43 | S: L306 | Alive | Pattani | 16/12/2021 |
| COVPSU-01193 | female | 2 | S: L306 | Alive | Pattani | 16/12/2021 |
| COVPSU-01194 | female | 48 | S: L306 | Alive | Pattani | 18/12/2021 |
| COVPSU-01196 | female | 43 | S: L306 | Alive | Pattani | 18/12/2021 |
| COVPSU-01197 | female | 22 | S: F306 | Alive | Pattani | 17/12/2021 |
| COVPSU-01198 | female | 19 | S: L306 | Alive | Pattani | 17/12/2021 |
| COVPSU-01199 | male | 71 | S: F306 | Alive | Pattani | 17/12/2021 |
| COVPSU-01201 | female | 24 | S: L306 | Alive | Pattani | 19/12/2021 |
| COVPSU-01202 | male | 55 | S: L306 | Alive | Pattani | 19/12/2021 |
| COVPSU-01203 | male | 51 | S: F306 | Alive | Pattani | 19/12/2021 |
| COVPSU-01204 | male | 26 | S: F306 | Alive | Yala | 17/12/2021 |
| COVPSU-01205 | female | 10 | S: F306 | Alive | Yala | 17/12/2021 |
| COVPSU-01206 | male | NA | S: F306 | Alive | Songkhla | 20/12/2021 |
| COVPSU-01207 | female | 27 | S: F306 | Alive | Yala | 16/12/2021 |
| COVPSU-01208 | male | 48 | S: F306 | Alive | Yala | 16/12/2021 |
| COVPSU-01209 | female | 14 | S: F306 | Alive | Yala | 17/12/2021 |
| COVPSU-01210 | male | 48 | S: F306 | Alive | Narathiwat | 14/12/2021 |
| COVPSU-01215 | female | 63 | S: F306 | Alive | Narathiwat | 20/12/2021 |
| COVPSU-01221 | female | 21 | S: F306 | Alive | Narathiwat | 13/12/2021 |
| COVPSU-01222 | male | 87 | S: L306 | Alive | Narathiwat | 12/12/2021 |
| COVPSU-01223 | male | 24 | S: F306 | Alive | Narathiwat | 15/12/2021 |
| COVPSU-01224 | male | 50 | S: F306 | Alive | Narathiwat | 19/12/2021 |
| COVPSU-01225 | female | 31 | S: L306 | Alive | Songkhla | 20/12/2021 |
| COVPSU-01226 | male | NA | S: F306 | Alive | Songkhla | 17/12/2021 |
| COVPSU-01228 | female | NA | S: F306 | Alive | Songkhla | 14/12/2021 |
| COVPSU-01232 | male | 39 | S: L306 | Alive | Songkhla | 17/12/2021 |
| COVPSU-01233 | male | NA | S: L306 | Alive | Songkhla | 20/12/2021 |
| COVPSU-01234 | male | 24 | S: F306 | Alive | Songkhla | 23/12/2021 |
| COVPSU-01235 | female | 46 | S: L306 | Alive | Songkhla | 16/12/2021 |
| COVPSU-01236 | male | 39 | S: F306 | Alive | Songkhla | 20/12/2021 |
| COVPSU-01238 | male | 43 | S: F306 | Alive | Pattani | 16/12/2021 |
| COVPSU-01239 | male | 71 | S: F306 | Alive | Pattani | 19/12/2021 |
| COVPSU-01240 | female | 41 | S: F306 | Alive | Pattani | 20/12/2021 |
| COVPSU-01241 | female | NA | S: F306 | Alive | Songkhla | 21/12/2021 |
| COVPSU-01242 | male | 32 | S: L306 | Alive | Songkhla | 21/12/2021 |
| COVPSU-01244 | female | 29 | S: F306 | Alive | Songkhla | 22/12/2021 |
| COVPSU-01245 | female | NA | S: L306 | Alive | Songkhla | 22/12/2021 |
| COVPSU-01246 | male | 69 | S: F306 | Alive | Songkhla | 22/12/2021 |
| COVPSU-01247 | female | 72 | S: F306 | Alive | Songkhla | 22/12/2021 |
| COVPSU-01248 | female | 49 | S: F306 | Alive | Songkhla | 22/12/2021 |
| COVPSU-01250 | female | 13 | S: L306 | Alive | Songkhla | 24/12/2021 |
| COVPSU-01251 | female | 68 | S: F306 | Alive | Songkhla | 24/12/2021 |
| COVPSU-01252 | female | 58 | S: F306 | Alive | Songkhla | 24/12/2021 |
| COVPSU-01253 | male | 49 | S: F306 | Alive | Songkhla | 24/12/2021 |
| COVPSU-01254 | female | 11 | S: F306 | Alive | Songkhla | 17/12/2021 |
| COVPSU-01255 | female | NA | S: F306 | Alive | Songkhla | 17/12/2021 |
| COVPSU-01256 | female | 55 | S: L306 | Alive | Songkhla | 18/12/2021 |
| COVPSU-01257 | female | NA | S: F306 | Alive | Songkhla | 21/12/2021 |
| COVPSU-01258 | female | 32 | S: F306 | Alive | Songkhla | 21/12/2021 |
| COVPSU-01259 | female | 35 | S: F306 | Alive | Songkhla | 21/12/2021 |
| COVPSU-01260 | female | 77 | S: F306 | Alive | Yala | 10/12/2021 |
| COVPSU-01261 | female | 57 | S: L306 | Alive | Songkhla | 17/12/2021 |
| COVPSU-01263 | female | 49 | S: F306 | Alive | Phatthalung | 19/12/2021 |
| COVPSU-01264 | male | 20 | S: L306 | Alive | Songkhla | 20/12/2021 |
| COVPSU-01265 | male | 31 | S: L306 | Alive | Songkhla | 20/12/2021 |
| COVPSU-01266 | male | 18 | S: L306 | Alive | Songkhla | 20/12/2021 |
| COVPSU-01269 | female | 60 | S: F306 | Alive | Phatthalung | 21/12/2021 |
| COVPSU-01270 | female | 53 | S: F306 | Alive | Pattani | 21/12/2021 |
| COVPSU-01272 | male | 57 | S: F306 | Alive | Phatthalung | 21/12/2021 |
| COVPSU-01273 | female | 25 | S: L306 | Alive | Phatthalung | 21/12/2021 |
| COVPSU-01276 | female | 30 | S: F306 | Alive | Songkhla | 21/12/2021 |
| COVPSU-01278 | male | 16 | S: L306 | Alive | Pattani | 23/12/2021 |
| COVPSU-01280 | female | 3 | S: L306 | Alive | Phatthalung | 23/12/2021 |
| COVPSU-01281 | female | 86 | S: L306 | Alive | Phatthalung | 23/12/2021 |
| COVPSU-01282 | female | 20 | S: F306 | Alive | Phatthalung | 24/12/2021 |
| COVPSU-01284 | female | 20 | S: F306 | Alive | Phatthalung | 24/12/2021 |
| COVPSU-01286 | female | 32 | S: L306 | Alive | Songkhla | 23/12/2021 |
| COVPSU-01287 | female | 63 | S: L306 | Alive | Songkhla | 25/12/2021 |
| COVPSU-01290 | male | NA | S: F306 | Alive | Songkhla | 27/12/2021 |
| COVPSU-01291 | male | NA | S: F306 | Alive | Songkhla | 27/12/2021 |
| COVPSU-01292 | male | NA | S: F306 | Alive | Songkhla | 27/12/2021 |
| COVPSU-01293 | male | NA | S: F306 | Alive | Songkhla | 27/12/2021 |
| COVPSU-01294 | female | NA | S: F306 | Alive | Songkhla | 27/12/2021 |
| COVPSU-01295 | male | NA | S: F306 | Alive | Songkhla | 27/12/2021 |
| COVPSU-01297 | female | 27 | S: F306 | Alive | Songkhla | 27/12/2021 |
| COVPSU-01298 | female | 52 | S: L306 | Alive | Pattani | 27/12/2021 |
| COVPSU-01299 | female | 31 | S: F306 | Alive | Songkhla | 25/12/2021 |
| COVPSU-01300 | male | 49 | S: L306 | Alive | Narathiwat | 22/12/2021 |
| COVPSU-01301 | male | 19 | S: F306 | Alive | Narathiwat | 28/12/2021 |
| COVPSU-01302 | female | 35 | S: L306 | Alive | Narathiwat | 21/12/2021 |
| COVPSU-01304 | male | 32 | S: F306 | Alive | Narathiwat | 26/12/2021 |
| COVPSU-01307 | male | 21 | S: F306 | Alive | Narathiwat | 21/12/2021 |
| COVPSU-01308 | male | 22 | S: F306 | Alive | Narathiwat | 21/12/2021 |
| COVPSU-01309 | male | 40 | S: F306 | Alive | Narathiwat | 21/12/2021 |
| COVPSU-01310 | female | 34 | S: F306 | Alive | Narathiwat | 21/12/2021 |
| COVPSU-01311 | female | 41 | S: L306 | Alive | Narathiwat | 23/12/2021 |
| COVPSU-01312 | male | 72 | S: F306 | Alive | Narathiwat | 24/12/2021 |
| COVPSU-01313 | male | 7 | S: L306 | Alive | Narathiwat | 24/12/2021 |
| COVPSU-01314 | female | 5 | S: L306 | Alive | Narathiwat | 24/12/2021 |
| COVPSU-01315 | male | 39 | S: F306 | Alive | Narathiwat | 25/12/2021 |
| COVPSU-01316 | male | 28 | S: F306 | Alive | Narathiwat | 24/12/2021 |
| COVPSU-01317 | female | 33 | S: F306 | Alive | Songkhla | 28/12/2021 |
| COVPSU-01318 | male | 89 | S: L306 | Death | Yala | 14/11/2021 |
| COVPSU-01319 | female | NA | S: F306 | Alive | Songkhla | 30/12/2021 |
| COVPSU-01320 | male | 83 | S: F306 | Alive | Songkhla | 29/12/2021 |
| COVPSU-01321 | male | 36 | S: F306 | Alive | Songkhla | 29/12/2021 |
| COVPSU-01322 | female | 43 | S: L306 | Alive | Songkhla | 27/12/2021 |
| COVPSU-01323 | female | 44 | S: F306 | Alive | Songkhla | 28/12/2021 |
| COVPSU-01324 | female | 41 | S: L306 | Alive | Songkhla | 28/12/2021 |
| COVPSU-01325 | female | 7 | S: F306 | Alive | Songkhla | 29/12/2021 |
| COVPSU-01326 | female | 41 | S: F306 | Alive | Songkhla | 30/12/2021 |
| COVPSU-01327 | male | 36 | S: F306 | Alive | Songkhla | 29/12/2021 |
| COVPSU-01330 | female | 84 | S: L306 | Alive | Phatthalung | 25/12/2021 |
| COVPSU-01331 | female | 28 | S: F306 | Alive | Phatthalung | 25/12/2021 |
| COVPSU-01332 | female | 89 | S: L306 | Alive | Phatthalung | 26/12/2021 |
| COVPSU-01333 | male | 25 | S: F306 | Alive | Phatthalung | 26/12/2021 |
| COVPSU-01334 | female | 34 | S: F306 | Alive | Phatthalung | 26/12/2021 |
| COVPSU-01338 | male | 33 | S: F306 | Alive | Pattani | 27/12/2021 |
| COVPSU-01340 | female | 74 | S: F306 | Alive | Phatthalung | 27/12/2021 |
| COVPSU-01343 | male | 43 | S: F306 | Alive | Pattani | 28/12/2021 |
| COVPSU-01344 | female | 56 | S: F306 | Alive | Phatthalung | 29/12/2021 |
| COVPSU-01345 | male | 42 | S: F306 | Alive | Phatthalung | 29/12/2021 |
| COVPSU-01346 | female | 49 | S: L306 | Alive | Phatthalung | 29/12/2021 |
| COVPSU-01347 | female | 37 | S: L306 | Alive | Pattani | 30/12/2021 |
| COVPSU-01348 | female | 28 | S: F306 | Alive | Phatthalung | 30/12/2021 |
| COVPSU-01350 | male | 24 | S: F306 | Alive | Songkhla | 2/1/2022 |
| COVPSU-01352 | male | 87 | S: L306 | Alive | Songkhla | 2/1/2022 |
| COVPSU-01353 | male | NA | S: L306 | Alive | Songkhla | 2/1/2022 |
| COVPSU-01354 | female | NA | S: L306 | Alive | Songkhla | 2/1/2022 |
| COVPSU-01355 | female | 36 | S: L306 | Alive | Songkhla | 29/12/2021 |
| COVPSU-01356 | female | NA | S: F306 | Alive | Songkhla | 2/1/2022 |
| COVPSU-01357 | female | NA | S: F306 | Alive | Songkhla | 31/12/2021 |
| COVPSU-01358 | male | NA | S: L306 | Alive | Yala | 16/12/2021 |
| COVPSU-01359 | male | NA | S: F306 | Alive | Yala | 18/12/2021 |
| COVPSU-01360 | male | NA | S: F306 | Alive | Yala | 19/12/2021 |
| COVPSU-01361 | male | NA | S: L306 | Alive | Yala | 20/12/2021 |
| COVPSU-01362 | male | NA | S: L306 | Alive | Yala | 20/12/2021 |
| COVPSU-01364 | female | NA | S: L306 | Alive | Yala | 22/12/2021 |
| COVPSU-01365 | male | NA | S: L306 | Alive | Yala | 24/12/2021 |
| COVPSU-01366 | male | NA | S: F306 | Alive | Yala | 24/12/2021 |
| COVPSU-01367 | female | NA | S: F306 | Alive | Yala | 27/12/2021 |
| COVPSU-01368 | female | NA | S: L306 | Alive | Yala | 27/12/2021 |
| COVPSU-01369 | female | NA | S: L306 | Alive | Yala | 28/12/2021 |
| COVPSU-01370 | female | NA | S: F306 | Alive | Yala | 29/12/2021 |
| COVPSU-01371 | female | NA | S: F306 | Alive | Songkhla | 31/12/2021 |
| COVPSU-01372 | female | NA | S: F306 | Alive | Songkhla | 1/1/2022 |
| COVPSU-01373 | female | NA | S: F306 | Alive | Songkhla | 1/1/2022 |
| COVPSU-01374 | male | NA | S: F306 | Alive | Songkhla | 2/1/2022 |
| COVPSU-01375 | male | 24 | S: F306 | Alive | Narathiwat | 31/12/2021 |
| COVPSU-01376 | male | 48 | S: L306 | Alive | Narathiwat | 29/12/2021 |
| COVPSU-01377 | female | 31 | S: L306 | Alive | Narathiwat | 29/12/2021 |
| COVPSU-01378 | female | 27 | S: F306 | Alive | Narathiwat | 31/12/2021 |
| COVPSU-01379 | female | 82 | S: F306 | Alive | Narathiwat | 31/12/2021 |
| COVPSU-01380 | male | 31 | S: L306 | Alive | Narathiwat | 3/1/2022 |
| COVPSU-01381 | female | 45 | S: F306 | Alive | Narathiwat | 27/12/2021 |
| COVPSU-01383 | female | 11 | S: F306 | Alive | Narathiwat | 31/12/2021 |
| COVPSU-01384 | female | NA | S: F306 | Alive | Songkhla | 5/1/2022 |
| COVPSU-01385 | female | NA | S: L306 | Alive | Songkhla | 5/1/2022 |
| COVPSU-01387 | female | NA | S: L306 | Alive | Yala | 4/1/2022 |
| COVPSU-01388 | male | NA | S: L306 | Alive | Yala | 4/1/2022 |
| COVPSU-01389 | male | NA | S: F306 | Alive | Songkhla | 4/1/2022 |
| COVPSU-01390 | male | NA | S: F306 | Alive | Songkhla | 5/1/2022 |
| COVPSU-01391 | male | NA | S: L306 | Alive | Songkhla | 4/1/2022 |
| COVPSU-01392 | female | 20 | S: F306 | Alive | Songkhla | 23/1/2022 |
| COVPSU-01402 | female | 34 | S: F306 | Alive | Songkhla | 3/1/2022 |
| COVPSU-01404 | male | 12 | S: F306 | Alive | Songkhla | 5/1/2022 |
| COVPSU-01414 | female | NA | S: F306 | Alive | Songkhla | 7/1/2022 |
| COVPSU-01424 | female | NA | S: L306 | Alive | Songkhla | 4/1/2022 |
| COVPSU-01427 | female | NA | S: F306 | Alive | Songkhla | 4/1/2022 |
| COVPSU-01432 | female | NA | S: F306 | Alive | Songkhla | 30/12/2021 |
| COVPSU-01436 | male | NA | S: L306 | Alive | Songkhla | 6/1/2022 |
| COVPSU-01443 | male | 62 | S: F306 | Alive | Pattani | 24/12/2021 |
| COVPSU-01444 | female | 49 | S: F306 | Alive | Pattani | 25/12/2021 |
| COVPSU-01445 | male | 11 | S: L306 | Alive | Pattani | 28/12/2021 |
| COVPSU-01453 | female | 48 | S: L306 | Alive | Songkhla | 6/1/2022 |
| COVPSU-01455 | female | NA | S: F306 | Alive | Songkhla | 8/1/2022 |
| COVPSU-01457 | female | NA | S: L306 | Alive | Songkhla | 8/1/2022 |
| COVPSU-01458 | female | NA | S: L306 | Alive | Songkhla | 8/1/2022 |
| COVPSU-01470 | female | NA | S: L306 | Alive | Songkhla | 4/1/2022 |
| COVPSU-01475 | female | 9 | S: F306 | Alive | Songkhla | 2/1/2022 |
| COVPSU-01476 | female | 19 | S: F306 | Alive | Phatthalung | 2/1/2022 |
| COVPSU-01477 | male | 56 | S: F306 | Alive | Phatthalung | 2/1/2022 |
| COVPSU-01479 | female | 80 | S: F306 | Alive | Phatthalung | 4/1/2022 |
| COVPSU-01490 | male | 15 | S: L306 | Alive | Pattani | 6/1/2022 |
| COVPSU-01491 | female | 20 | S: F306 | Alive | Pattani | 5/1/2022 |
| COVPSU-01492 | female | 28 | S: F306 | Alive | Phatthalung | 6/1/2022 |
| COVPSU-01494 | female | 18 | S: F306 | Alive | Phatthalung | 7/1/2022 |
| COVPSU-01496 | female | 1 | S: L306 | Alive | Songkhla | 1/1/2022 |
| COVPSU-01503 | female | 34 | S: F306 | Alive | Songkhla | 4/1/2022 |
| COVPSU-01506 | female | NA | S: F306 | Alive | Songkhla | 8/1/2022 |
| COVPSU-01892 | female | NA | S: F306 | Alive | Songkhla | 11/1/2022 |
| COVPSU-01899 | female | NA | S: F306 | Alive | Songkhla | 10/1/2022 |
| COVPSU-01903 | male | 38 | S: F306 | Alive | Songkhla | 14/1/2022 |
| COVPSU-01904 | female | 42 | S: F306 | Alive | Songkhla | 11/1/2022 |
| COVPSU-01910 | female | 68 | S: F306 | Alive | Songkhla | 11/1/2022 |
| COVPSU-01914 | male | 12 | S: F306 | Alive | Songkhla | 12/1/2022 |
| COVPSU-01943 | male | 80 | S: F306 | Alive | Phatthalung | 13/1/2022 |
| COVPSU-01944 | female | 44 | S: F306 | Alive | Phatthalung | 13/1/2022 |
| COVPSU-01946 | male | 80 | S: F306 | Alive | Phatthalung | 14/1/2022 |
| COVPSU-01947 | male | 36 | S: F306 | Alive | Phatthalung | 14/1/2022 |
| COVPSU-01950 | female | NA | S: F306 | Alive | Songkhla | 13/1/2022 |
| COVPSU-01976 | female | 41 | S: L306 | Alive | Yala | 10/1/2022 |
| COVPSU-01979 | male | 25 | S: L306 | Alive | Yala | 10/1/2022 |
| COVPSU-01984 | male | 8 | S: F306 | Alive | Yala | 10/1/2022 |
| COVPSU-01985 | male | 59 | S: F306 | Alive | Songkhla | 16/1/2022 |
| COVPSU-01991 | male | 49 | S: F306 | Alive | Songkhla | 6/1/2022 |
| COVPSU-02005 | male | NA | S: F306 | Alive | Songkhla | 19/1/2022 |
| COVPSU-02025 | male | 45 | S: F306 | Alive | Yala | 19/1/2022 |
| COVPSU-02034 | female | 35 | S: L306 | Alive | Songkhla | 16/1/2022 |
| COVPSU-02052 | female | 19 | S: F306 | Alive | Songkhla | 18/1/2022 |
| COVPSU-02054 | female | 18 | S: F306 | Alive | Songkhla | 18/1/2022 |
| COVPSU-02055 | female | 40 | S: F306 | Alive | Songkhla | 18/1/2022 |
| COVPSU-02063 | female | 20 | S: F306 | Alive | Phatthalung | 15/1/2022 |
| COVPSU-02064 | female | 45 | S: F306 | Alive | Phatthalung | 15/1/2022 |
| COVPSU-02066 | female | 56 | S: L306 | Alive | Phatthalung | 15/1/2022 |
| COVPSU-02067 | male | 58 | S: L306 | Alive | Phatthalung | 16/1/2022 |
| COVPSU-02068 | female | 11 | S: L306 | Alive | Phatthalung | 16/1/2022 |
| COVPSU-02071 | female | 33 | S: F306 | Alive | Phatthalung | 17/1/2022 |
| COVPSU-02072 | male | 37 | S: F306 | Alive | Phatthalung | 17/1/2022 |
| COVPSU-02075 | male | 68 | S: F306 | Alive | Phatthalung | 18/1/2022 |
| COVPSU-02078 | female | 77 | S: L306 | Alive | Phatthalung | 19/1/2022 |
| COVPSU-02084 | female | 35 | S: L306 | Alive | Phatthalung | 20/1/2022 |
| COVPSU-02086 | male | 40 | S: F306 | Alive | Phatthalung | 21/1/2022 |
| COVPSU-02105 | female | 22 | S: F306 | Alive | Yala | 19/1/2022 |
| COVPSU-02115 | female | NA | S: F306 | Alive | Songkhla | 14/1/2022 |
| COVPSU-02120 | female | 32 | S: F306 | Alive | Yala | 19/1/2022 |
| COVPSU-02121 | female | 34 | S: F306 | Alive | Yala | 23/1/2022 |
| COVPSU-02128 | female | 58 | S: F306 | Death | Songkhla | 22/1/2022 |
| COVPSU-02146 | female | 53 | S: F306 | Alive | Yala | 24/1/2022 |
| COVPSU-02150 | male | 16 | S: F306 | Alive | Pattani | 22/1/2022 |
| COVPSU-02151 | male | NA | S: L306 | Alive | Pattani | 22/1/2022 |
| COVPSU-02152 | female | 30 | S: L306 | Alive | Phatthalung | 22/1/2022 |
| COVPSU-02157 | female | 25 | S: F306 | Alive | Phatthalung | 23/1/2022 |
| COVPSU-02159 | male | 44 | S: L306 | Alive | Phatthalung | 23/1/2022 |
| COVPSU-02160 | male | 41 | S: F306 | Alive | Phatthalung | 23/1/2022 |
| COVPSU-02164 | female | 11 | S: F306 | Alive | Phatthalung | 24/1/2022 |
| COVPSU-02165 | female | 64 | S: F306 | Alive | Phatthalung | 24/1/2022 |
| COVPSU-02171 | female | 52 | S: F306 | Alive | Phatthalung | 26/1/2022 |
| COVPSU-02178 | female | 69 | S: F306 | Alive | Phatthalung | 28/1/2022 |
| COVPSU-02189 | female | 10 | S: F306 | Alive | Songkhla | 30/1/2022 |
| COVPSU-02190 | female | 10 | S: F306 | Alive | Songkhla | 30/1/2022 |
| COVPSU-02193 | female | 91 | S: F306 | Alive | Songkhla | 26/1/2022 |
| COVPSU-02201 | male | 60 | S: L306 | Alive | Pattani | 27/1/2022 |
| COVPSU-02204 | male | 34 | S: F306 | Alive | Songkhla | 3/2/2022 |
| COVPSU-02226 | male | 42 | S: F306 | Alive | Songkhla | 5/2/2022 |
| COVPSU-02235 | male | 69 | S: F306 | Alive | Yala | 22/1/2022 |
| COVPSU-02248 | male | 73 | S: L306 | Alive | Narathiwat | 7/2/2022 |
| COVPSU-02255 | female | 86 | S: F306 | Alive | Songkhla | 10/2/2022 |
| COVPSU-02257 | female | 69 | S: F306 | Alive | Songkhla | 10/2/2022 |
| CSEQ114 | male | 63 | S: F306 | Alive | Songkhla | 24/6/2021 |
| CSEQ133 | female | 59 | S: F306 | Alive | Songkhla | 25/6/2021 |

**Supplementary Table S3.** HADDOCK 2.4 settings and options used in molecular docking analyses.

| **Docking parameters** | **Values** |
| --- | --- |
| **1. Distance restraints** | |
| 1.1 Remove non-polar hydrogens | Supported |
| 1.2 Force constant for center of mass contact restraints | 1.0 |
| 1.3 Force constant for surface contact restraints | 1.0 |
| 1.4 Randomly exclude a fraction of the ambiguous restraints (AIRs) | Supported |
| 1.5 Number of partitions for random exclusion (%excluded=100/number of partitions) | 2.0000 |
| 1.6 Radius of gyration | 17.78 |
| **2. Sampling parameters** | |
| 2.1 Number of structures for rigid body docking | 1000 |
| 2.2 Number of trials for rigid body minimization | 5 |
| 2.3 Sample 180 degrees rotated solutions during rigid body EM | Supported |
| 2.4 Number of structures for semi-flexible refinement | 200 |
| 2.5 Perform final refinement | Supported |
| 2.6 Number of structures for the final refinement | 200 |
| 2.7 Number of structures to analyze | 200 |
| 2.8 Cutoff for defining neighboring flexible regions | 5.0 |
| **3. Clustering parameters** | |
| 3.1 Clustering method (RMSD or Fraction of Common Contacts (FCC)) | FCC |
| 3.2 RMSD Cutoff for clustering | 0.60 |
| 3.3 Minimum cluster size | 4 |
| **4. Energy and interaction parameters** | |
| 4.1 Nonbonded parameters | OPLSX |
| 4.2 Include electrostatic during rigid body docking (it0) | Supported |
| 4.3 Include electrostatic during rigid body docking (it1) | Supported |
| 4.4 Use constant (cdie) or distance-dependent (rdie) dielectric in it0 | rdie |
| 4.5 Use constant (cdie) or distance-dependent (rdie) dielectric in it1 | rdie |
| 4.6 Epsilon constant for the electrostatic energy term in it0 | 10.0 |
| 4.7 Epsilon constant for the electrostatic energy term in it1 | 1.0 |
| 4.8 Scaling of intermolecular interactions for rigid body EM | 1.0 |
| 4.9 Rigid body dynamic, initial value | 0.001 |
| 4.10 SA with flexible side-chains (cool2), initial value | 0.001 |
| 4.11 SA with flexible backbone and side-chains (cool3), initial value | 0.05 |
| 4.12 Rigid body dynamic, final value | 0.001 |
| 4.13 SA with flexible side-chains (cool2), final value | 1.0 |
| 4.14 SA with flexible backbone and side-chains (cool3), final value | 1.0 |
| **5. Advanced sampling parameters** | |
| 5.1 Perform cross-docking | Supported |
| 5.2 Randomize starting orientations | Supported |
| 5.3 Perform initial rigid body minimization | Supported |
| 5.4 Allow translation in rigid body minimization | Supported |
| 5.5 Initial seed for random number generator | 917 |
| 5.6 Temperature for rigid body high temperature TAD | 2000 |
| 5.7 Initial temperature for rigid body first TAD cooling step | 2000 |
| 5.8 Final temperature after first cooling step | 500 |
| 5.9 Initial temperature for second TAD cooling step with flexible side-chain at the interface | 1000 |
| 5.10 Final temperature after second cooling step | 50 |
| 5.11 Initial temperature for third TAD cooling step with fully flexible interface | 1000 |
| 5.12 Final temperature after third cooling step | 50 |
| 5.13 Time step | 0.002 |
| 5.14 Factor for timestep in TAD | 8 |
| 5.15 Number of EM steps for translational minimization | 1000 |
| 5.16 Number of MD steps for rigid body high temperature TAD | 500 |
| 5.17 Number of MD steps during first rigid body cooling stage | 500 |
| 5.18 Number of MD steps during second cooling stage with flexible side-chains at interface | 1000 |
| 5.19 Number of MD steps during third cooling stage with fully flexible interface | 1000 |
| 5.20 Number of steps for heating phase (100, 200, 300K) | 100 |
| 5.21 Number of steps for 300K phase | 1250 |
| 5.22 Number of steps for cooling phase (300, 200, 100K) | 500 |
| **6. Solvated docking parameters** | |
| 6.1 Method | db |
| 6.2 Database method | Kyte-Doolittle |
| 6.3 Initial cutoff for restraints solvating method | 5.0 |
| 6.4 Cutoff for restraints solvating method | 5.0 |
| 6.5 Scale factor for restraints solvating method | 25.0 |
| 6.6 Fraction of water to keep in ntrial loop | 0.50 |
| 6.7 Fraction of water to keep in ntrial loop for DNA phosphates | 0.75 |
| 6.7 Water-surface-cutoff | 8.0 |
| 6.8 Use translation in loop miniwater | Supported |
| 6.9 How many different solvation shells to generate | 1 |
| **7. Analysis parameters** | |
| 7.1 Full or limited analysis of results | Cluster |
| 7.2 Cutoff distance (proton-acceptor) to define a hydrogen bond | 2.5 |
| 7.3 Cutoff distance (carbon-carbon) to define a hydrophobic contact | 3.9 |
| 7.4 COVID-19 related | Supported |

# Supplementary Figures


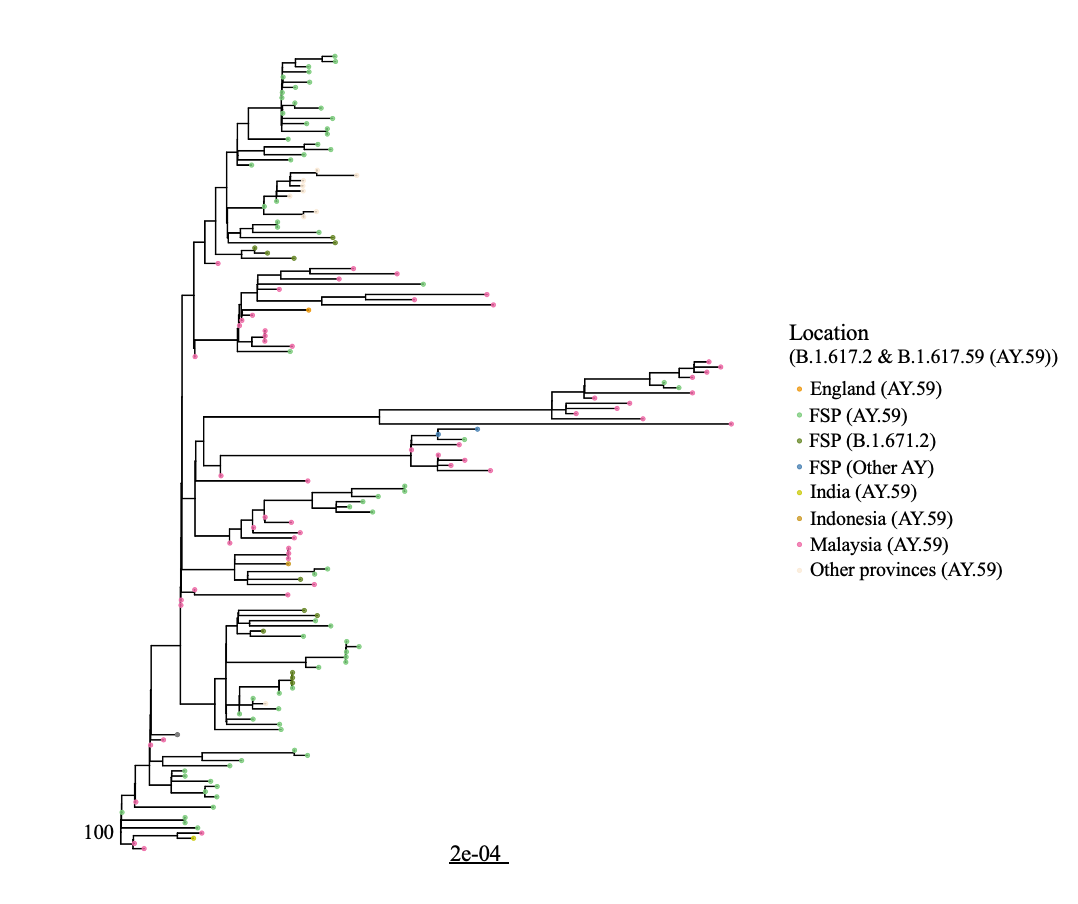


**Supplementary Figure S1** Phylogeographic analysis of the variant B.1.617.2 & B.1.617.2.59 (AY.59). Tip colours indicate sampling location and variants.


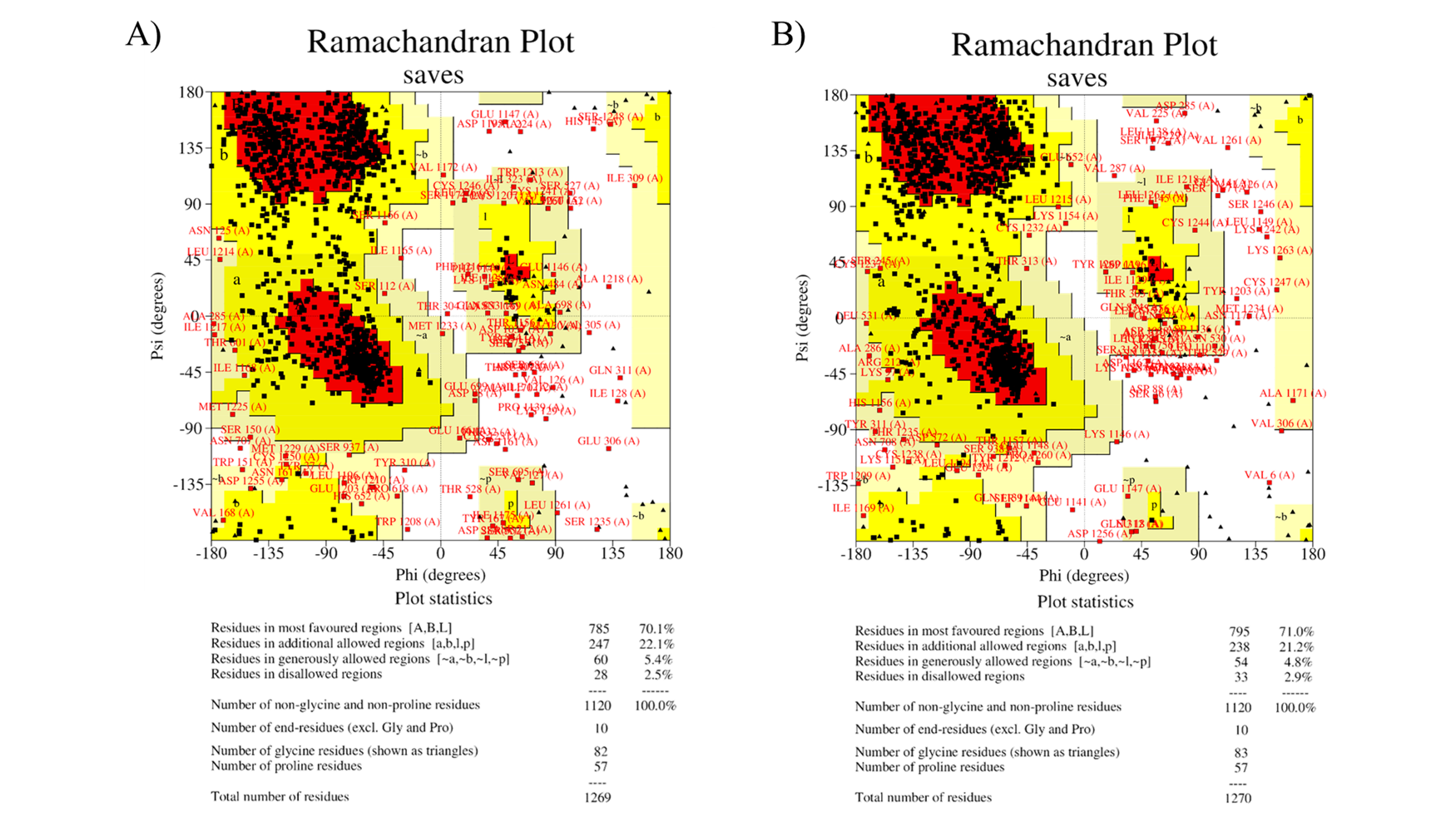


**Supplementary** **Figure S2** Ramachandran plots of the predicted tertiary structures of the spike proteins of AY.85-S: F306 (**A**), and AY.85-S: L306 (**B**) generated by PROCHECK (https://saves.mbi.ucla.edu/).


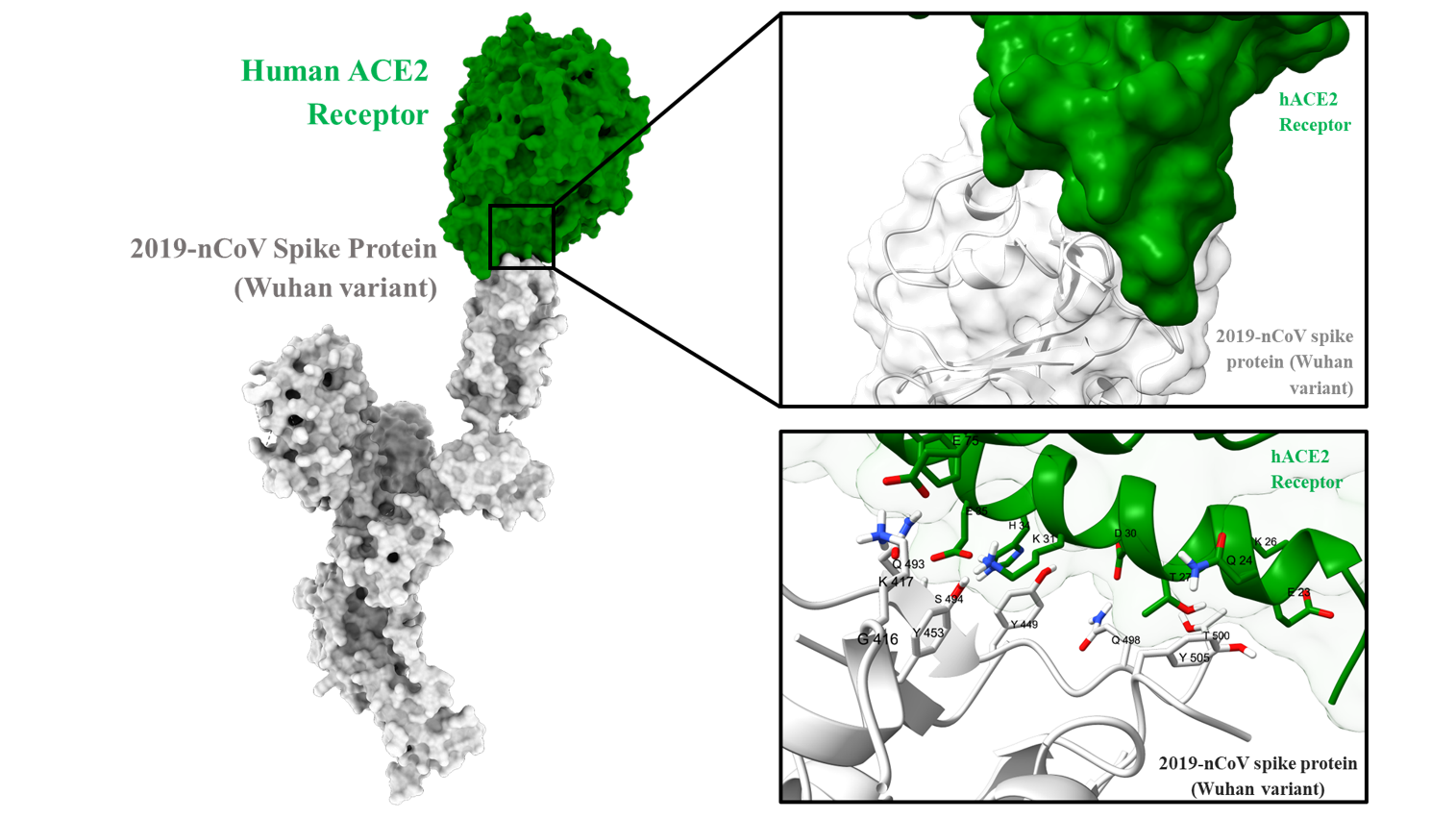


**Supplementary Figure S3.** Protein-protein docking analysis and binding interaction between the spike protein of the reference Wuhan-Hu-1 variant (gray) and the hACE2 receptor (green) as predicted by HADDOCK 2.4 (https://wenmr.science.uu.nl/haddock2.4/).


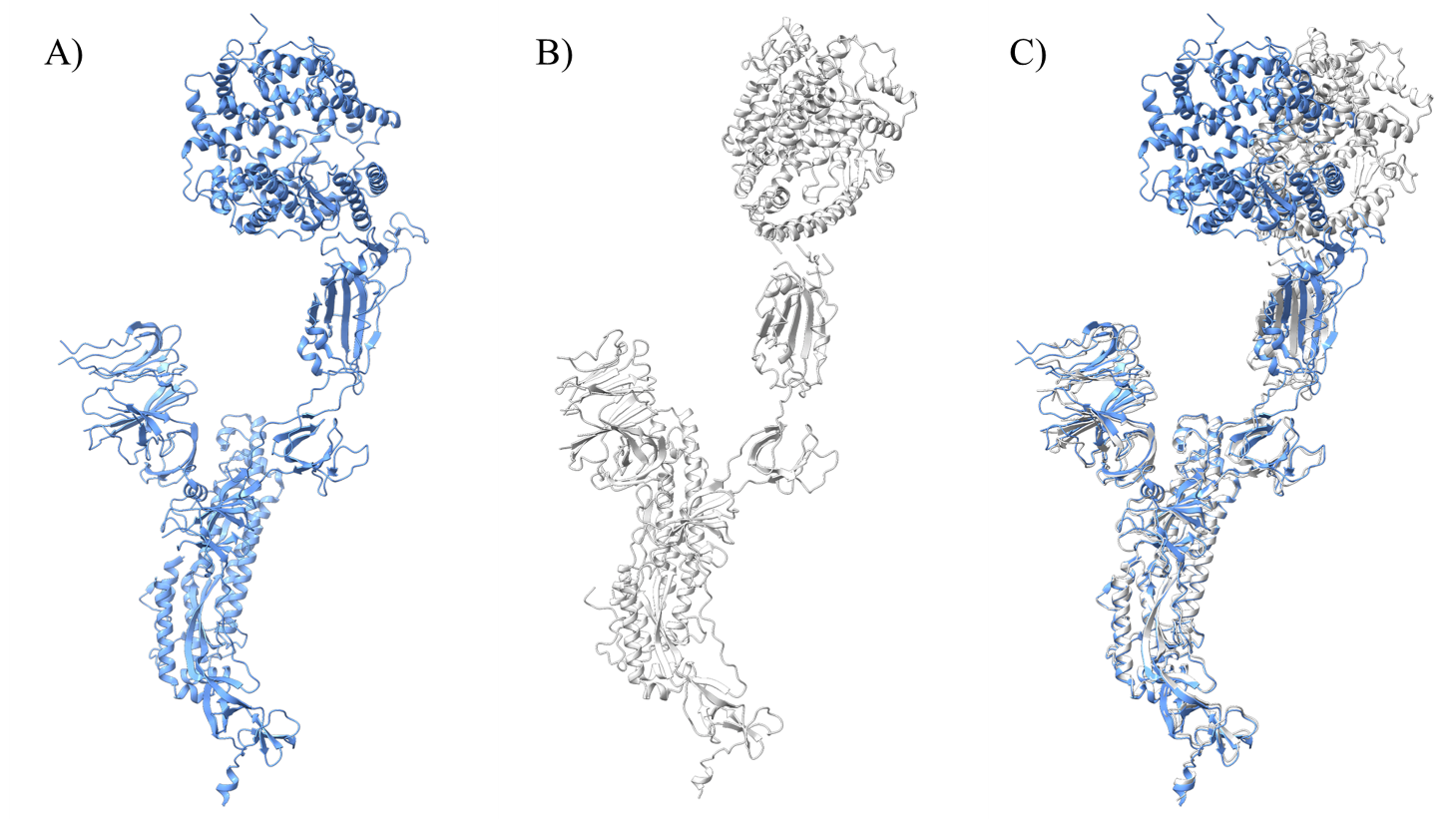


**Supplementary Figure S4** (**A**) Experimentally derived electron microscope-based protein complex structure of the spike protein of the reference Wuhan-Hu-1 variant and the hACE2 receptor. (**B**) The protein complex structure predicted by using HADDOCK 2.4 (https://wenmr.science.uu.nl/haddock2.4/). (**C**) Structural alignment of the two complexes

# Supplementary Note: Molecular docking analysis of the Wuhan-Hu-1 spike protein and the human ACE2 protein

The docking of the Wuhan-Hu-1 spike protein (PDB ID: 6VSB ^1^, resolution = 3.46 Å) to the human ACE2 receptor (PDB ID: 7W99^2^, resolution = 3.40 Å) was predicted using HADDOCK 2.4 (<https://wenmr.science.uu.nl/haddock2.4/>) ^3^ under default settings (**Supplementary Table S3**). In the analysis, we selected amino acid residues that were close to the known ACE2 receptor’s interaction interface ^4^ as “active residues”. The result is shown in **Supplementary Fig S3**, visualized by using UCSF ChimeraX (<https://www.cgl.ucsf.edu/chimerax/>).

To assess the quality of the predicted docking complex, we aligned the resultant model to the one derived experimentally (PDB ID: 7DX5 ^5^, **Supplementary Fig S4**), and computed the Root Mean Square Deviation (RMSD) using the *matchmaker* (or *mmaker*) command in ChimeraX ^6^. The following parameters were used in the analysis: Chain paring = bb; Alignment algorithm = Needleman-Wunsch; Similarity matrix = BLOSUM-62; SS fraction = 0.3; Gap open (HH/SS/other) = 18/18/6; Gap extend = 1; SS matrix = (H:H = 6, H:S = -9, H:O = -6, S:S = 6, S:O = -6, and O:O = 4); Iteration cutoff = 2. The quality of the predicted model was evaluated based on the RMSD obtained under the critical assessment of predicted interactions (CAPRI) criteria: incorrect if RMSD >10.0 Å; acceptable if RMSD = 5.0–10.0 Å; medium if RMSD = 1.0 – 5.0 Å; and high quality if RMSD <1.0 Å ^7^. The result was again visualized by using UCSF ChimeraX (<https://www.cgl.ucsf.edu/chimerax/>). Overall, we found that the RMSD between 712 pruned atoms pairs was 1.052 Å, and was 2.834 Å across all 941 pairs. These results were acceptable under the CAPRI criteria, validating this approach to molecular docking analysis as a suitable method.

## References

1 Wrapp, D. *et al.* Cryo-EM structure of the 2019-nCoV spike in the prefusion conformation. *Science* **367**, 1260-1263 (2020). <https://doi.org/10.1126/science.abb2507>

2 Wang, Y. *et al.* Structural basis for SARS-CoV-2 Delta variant recognition of ACE2 receptor and broadly neutralizing antibodies. *Nature Communications* **13**, 871 (2022). <https://doi.org/10.1038/s41467-022-28528-w>

3 van Zundert, G. C. P. *et al.* The HADDOCK2.2 Web Server: User-Friendly Integrative Modeling of Biomolecular Complexes. *J Mol Biol* **428**, 720-725 (2016). <https://doi.org/10.1016/j.jmb.2015.09.014>

4 Yi, C. *et al.* Key residues of the receptor binding motif in the spike protein of SARS-CoV-2 that interact with ACE2 and neutralizing antibodies. *Cellular & Molecular Immunology* **17**, 621-630 (2020). <https://doi.org/10.1038/s41423-020-0458-z>

5 Yan, R. *et al.* Structural basis for the different states of the spike protein of SARS-CoV-2 in complex with ACE2. *Cell Research* **31**, 717-719 (2021). <https://doi.org/10.1038/s41422-021-00490-0>

6 Pettersen, E. F. *et al.* UCSF ChimeraX: Structure visualization for researchers, educators, and developers. *Protein Sci* **30**, 70-82 (2021). <https://doi.org/10.1002/pro.3943>

7 Lensink, M. F., Méndez, R. & Wodak, S. J. Docking and scoring protein complexes: CAPRI 3rd Edition. *Proteins* **69**, 704-718 (2007). <https://doi.org/10.1002/prot.21804>
